# A Second Fungal Outbreak in Castañar Cave, Spain, Discloses the Fragility of Subsurface Ecosystems

**DOI:** 10.1007/s00248-024-02367-2

**Published:** 2024-03-20

**Authors:** Tamara Martin-Pozas, Alena Nováková, Valme Jurado, Soledad Cuezva, Angel Fernandez-Cortes, Cesareo Saiz-Jimenez, Sergio Sanchez-Moral

**Affiliations:** 1 Museo Nacional de Ciencias Naturales, MNCN-CSIC, 28006 Madrid, Spain; 2 Departamento de Biologia y Geologia, Universidad de Almeria, 04120 Almeria, Spain; 3 Laboratory of Fungal Genetics and Metabolism, Institute of Microbiology of the CAS, Prague, Czech Republic; 4 Instituto de Recursos Naturales y Agrobiologia, IRNAS-CSIC, 41012 Seville, Spain; 5 Spanish Geological Survey, IGME-CSIC, 28003 Madrid, Spain

**Keywords:** *Cephalotrichum microsporum*, *Neocosmospora solani*, Anthropogenic disturbances, Cave works, Ecological changes, Natural heritage

## Abstract

Castañar is a cave with strict visitor control measures since it was open to public visits in 2003. However, in recent years, the cave suffered two fungal outbreaks, the first in 2008 and controlled by cleaning the contaminated sediments and subsequent closure of the cave until 2014. The cave was reopened but limited to a maximum of 450 visitors/year. Despite these restrictions on visit, the cave experienced a second outbreak in 2021, originating from the installation of a steel grating walkway, aiming at protecting the ground sediments from the visitors’ footsteps. Here, we conducted an analysis using Next-Generation Sequencing and culture-dependent techniques to investigate the fungal communities related to the second outbreak and compare with those present before the cave suffered the outbreak. The results show that the most abundant fungi involved in the 2021 outbreak were already detected in 2020, and even in 2008 and 2009, although the main species that originating both outbreaks were different, likely due to the different carbon sources introduced into the cave.

**Supplementary Information:**

The online version contains supplementary material available at 10.1007/s00248-024-02367-2.

## Introduction

Caves and cultural heritage sites, known for their intricate ecosystems and invaluable historical relics, are currently facing an increasing threat: fungal outbreaks. Fungi, critical agents in organic matter decomposition, were observed with increasing frequency in these unique environments. Factors such as humidity fluctuations, inadequate ventilation, temperature variations, and human activities have created ideal conditions for fungal proliferation [1–3].

These outbreaks pose a substantial risk to the preservation of both natural mineral cave formations (speleothems) and culturally significant artifacts (rock art and engravings). Fungi have demonstrated the capacity to degrade organic materials and mineral substrates, leading to irreversible damage [4, 5]. Within caves and other subterranean environments (crypts, catacombs, tombs, etc.), fungal colonization disrupts the delicate equilibrium of ecosystems, impacting autochthonous species and colonizing sediments, speleothems, and walls [6–8].

A comprehensive understanding of the origin and consequences of fungal outbreaks in caves and cultural heritage sites is crucial to crafting effective preservation strategies. Conservationists, scientists, and heritage experts are diligently searching for mitigation methods, including enhanced environmental monitoring, preventive measures, and innovative restoration techniques. The protection of our shared natural and cultural heritage depends on our ability to tackle these fungal challenges accurately [1, 2, 9].

**Fig. 1:**
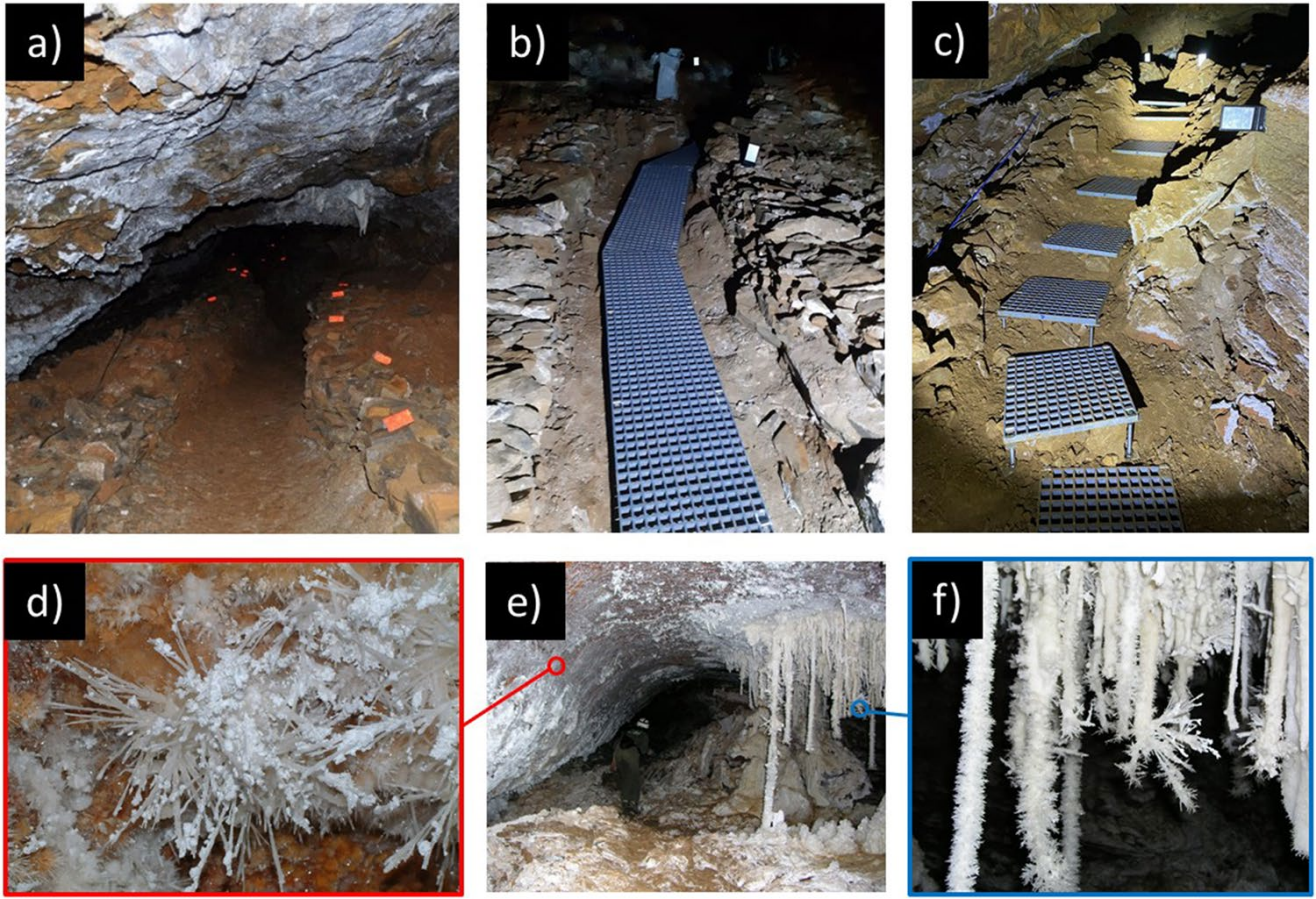
Steel grating walkway in Castañar Cave. **a** Before installation. **b**, **c** After installation. **e** View of *Sala del Jardin* with aragonite frostwork nucleating over the red clays (left wall) and a typical group of white speleothems including stalactites, stalagmites, and columns (near to the right wall). **d** Detailed view of radially aligned aragonite needles with thin, white coating of huntite thickening the individual aragonite needles. **f** Detailed view of aragonite anthodites covering stalactites

In this context, several notable show caves and subterranean sites have witnessed various microbial outbreaks in recent decades [10, 11]. The most notable fungal outbreaks include Lascaux and Castañar caves. These outbreaks, resulting from numerous factors, have posed serious threats to the conservation of these iconic sites. In particular, Lascaux and Altamira caves, famous worldwide for their exceptional Paleolithic art, experienced fungal and bacterial outbreaks after strong alterations of their environments due to extensive works for visitor facilities and wrong management [3, 12]. Castañar Cave is notable for its spectacular mineral morphologies (Fig. 1). This cave was classified as a natural monument due to its astonishing array of aragonite and calcite speleothems [13, 14]. In this case, the outbreak was caused by vomit from a visitor in 2008 [6]. These historical instances underscore the vulnerability of caves to microbial outbreaks and emphasize the need for meticulous management and preservation strategies.

In Lascaux, the appearance of the *Fusarium solani* and *Ochroconis lascauxensis* fungi put the conservation of the paintings and engravings at risk [3]. On the contrary, Castañar Cave experienced an outbreak of *Mucor circinelloides* and *F. solani* in 2008, which resulted in the closure of the cave [6]. Then, the strict visitor control measures implemented after the outbreak resulted in a significant reduction in visitation [15, 16].

A recent study of the Castañar Cave fungal community conducted 12 years after the initial outbreak in 2008 revealed the persistence of several fungi, including *Neocosmospora solani* (= *F. solani*), *Fusarium oxysporum*, and *Mortierella alpina* [17]. Unfortunately, a second outbreak occurred in Castañar Cave during the work on the installation of a steel grating walkway to protect the ground sediments and rocks from visitors’ footsteps. The work began on 19 July 2021, and the first observation of the second fungal outbreak dates back to 1 November 2021 (Fig. 1), while the work ended on 20 December 2021.

The primary objective of this study is to contrast the fungal outbreak observed in 2021 in Castañar Cave with the status of the fungal communities 1 year before the outbreak. For the purpose of this comparative analysis, data from the years 2008, 2009, 2020, and 2021 were studied. This article sheds light on the transformations within cave microbiomes as a result of the second fungal outbreak, emphasizing the critical need for vigilant conservation efforts and proactive measures in safeguarding our subterranean natural and cultural treasures.

## Methods

### Site and Sediment Sampling

Castañar Cave (SW Spain, 39°37′40″N, 5°24′59″W, 590 m.a.s.l.) is a natural cave formed by the dissolution of the dolomite beds interbedded within the shales and greywackes [13]. Castañar Cave is a low-energy cave characterized by high concentrations of ^222^Rn gas (33,000 Bq/m^3^, annual average) [15]. Data on geology, microscopy, and environmental parameters have been extensively reported elsewhere [13–15, 18].

On 16–17 September 2009, micromycete isolation was carried out from sediments at four sites inside the cave: *Sala de Entrada* (P1), *Galeria Principal*–vomit area (P2), *Sala Blanca* (P4), and *Sala del Jardin* (P6). Cave sediment samples were collected in high-density sterile polyethylene bags (additional description in Supplementary Table S1). It should be noted that data from 2009 (Supplementary Table S2) were obtained 5 months after the first outbreak, and the cave did not experience any further fungal outbreak before the 2020 sampling.

**Fig. 2:**
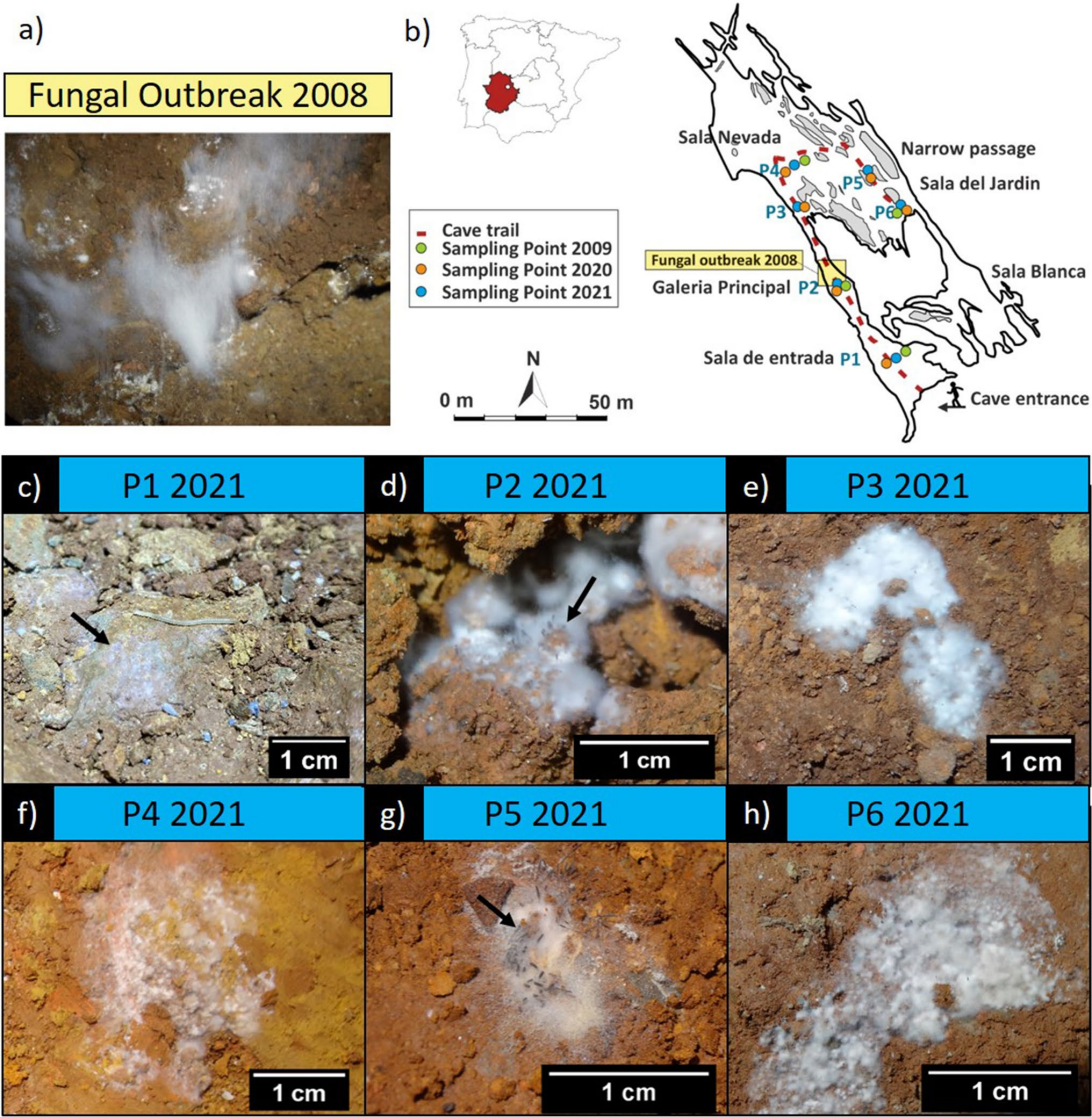
Spatial distribution of fungal sampling points. **a** Detail of a fungal outbreak on August 26, 2008, resulting from a vomit. **b** Map of Castañar Cave with the locations of sampling points in 2009, 2020, and 2021. **c**–**h** Details of fungal outbreaks at different locations on November 3, 2021, following installation works. Black arrow in (**c**) points to the fungal outbreak. Black arrows in (**d**) and (**g**) indicate synnemata of *Cephalotrichum microsporum*

On 3 December 2020, 12 sediment samples were collected in the cave for NGS. The sampling points were P1–P6 as described in Fig. 2 and Supplementary Table S1.

On 3 November 2021, a total of 19 sediment samples, representing fungal outbreaks and distributed in six primary zones, were sampled within Castañar Cave, as illustrated in Fig. 2 and Supplementary Table S1 (designated as P1–P6). It is noticeable that the *Galeria Principal*–vomit area (P2) and a connection from *Sala Nevada* to *Sala del Jardin*, through a narrow passage (P5)—were the regions most affected by the outbreak, as evidenced by a larger surface area colonized by fungal mycelia. Consequently, a larger number of samples were collected in these areas. For microscopy and culture purposes, sediment samples were sampled and preserved at 4 °C. Meanwhile, samples intended for Next-Generation Sequencing (NGS) were aseptically collected using sterile polypropylene tubes and spatulas. The NGS samples were suspended in DNA/RNA Shield™ and maintained at 4 °C during transport and then preserved at − 80 °C within the laboratory.

### Microscopy

Sediment samples were fixed with 4% formaldehyde in PBS and dehydrated through a series of graded ethanol. Hexamethyldisilazane (HMDS) was employed for drying. The processed sediments were mounted on scanning electron microscopy (SEM) sample holders and coated with a thin layer of gold carbon to enhance conductivity and imaging quality. SEM analysis was performed using the FEI INSPECT Scanning Electron Microscope (MNCN-CSIC, Spain).

### Culture Techniques

Several methods were used: the dilution plate method [19] and the cellulose and keratin bait technique for the follow-up isolation from cave sediment (this isolation technique was carried out in laboratory microcosms). Beer-wort agar (with rose Bengal and chloramphenicol added for bacterial suppression) was used as isolation medium for soil-borne fungi. All isolated microfungal strains were cultivated on malt extract agar (MEA); Czapek yeast autolysate agar (CYA), malt extract agar, Czapek-Dox agar (CZA), potato dextrose agar (PDA), while yeast extract sucrose agar (YES) was used as identification media [20, 21]. Microfungal identification was carried out according to macromorphological and micromorphological properties, and strains of the genus *Aspergillus* were identified using molecular methods, as described by Sklenář et al. [22] (Supplementary Table S3).

DNA was extracted from 7-day-old colonies using the Microbial DNA Isolation Kit (Mo-Bio Laboratories, Inc.). The PCR products, purification, and sequencing were provided by Macrogen Europe, The Netherlands. Sequences were inspected and assembled using the Bioedit sequence alignment editor v7.0.0 (https://bioedit.software.informer.com/7.0/). For phylogenetic analysis, we employed ITS1/NL4, which amplify the ITS region and two other primers to amplify a region of the benA gene, which encoded a subunit of β-tubulin, and a region of the caM gene, which encodes calmodulin. Both primers were used in recent taxonomic monographs for the identification and classification of fungi and can provide complementary phylogenetic information [23, 24]. Additionally, the choice of primers allowed us to compare the culture data from 2021 with those from 2009.

### Next-Generation Sequencing

Total DNA was extracted from environmental samples following the manufacturer’s guidelines, employing the QIAGEN Power Soil Kit. Of the 12 samples collected in 2020, only 10 contained enough DNA for NGS. The extracted DNA served as a template for the generation of PCR amplicons, using the internal transcribed spacer 2 (ITS2) of the nuclear ribosomal DNA as the DNA barcode for identification of molecular species. PCR amplicons, approximately 400 base pairs in length, were generated using the universal primers ITS86F and ITS4, as previously detailed in Martin-Pozas et al. [25]. Subsequently, the PCR amplicons underwent high-throughput sequencing, performed at AllGenetics Co. in A Coruña, Spain, utilizing an Illumina MiSeq sequencer.

To compare the fungi present during the second outbreak with the fungi existent before the outbreak, the raw fastq files obtained in 2021 were analyzed together with the raw reads obtained in 2020 at the same sampling points, simultaneously. The raw fastq were filtered and analyzed using QIIME2 version 2019.10 (https://qiime2.org/) [26]. Specifically, the qiime2-dada2 plugin was used to perform filtering, dereplication, and the removal of chimeric sequences [27]. Subsequently, taxonomic assignments were determined using the qiime2-feature-classifier classify-sklearn method against the UNITE fungal ITS database (version 9.0; http://unite.ut.ee/). The classifier was trained using naive Bayes on the complete reference sequences. All fungal nomenclature adhered to the conventions of the MycoBank Database (https://www.mycobank.org). The operational taxonomic units (OTUs) were further imported into the R environment version 3.6.0 and processed utilizing the phyloseq package. We calculated alpha diversity using the observed number of OTUs and the Shannon index and compared them with a Mann–Whitney test. Beta diversity was visualized using Principal Coordinates Analysis (PCoA) and Bray–Curtis ordination and analyzed using ANOSIM and PERMANOVA to test differences between fungal communities before and during the outbreak. Functional assignments for each OTU classified with QIIME2 were determined using the FUNGuild database [28].

## Results

### Fungal Identification by Scanning Electron Microscopy (SEM)

**Fig. 3:**
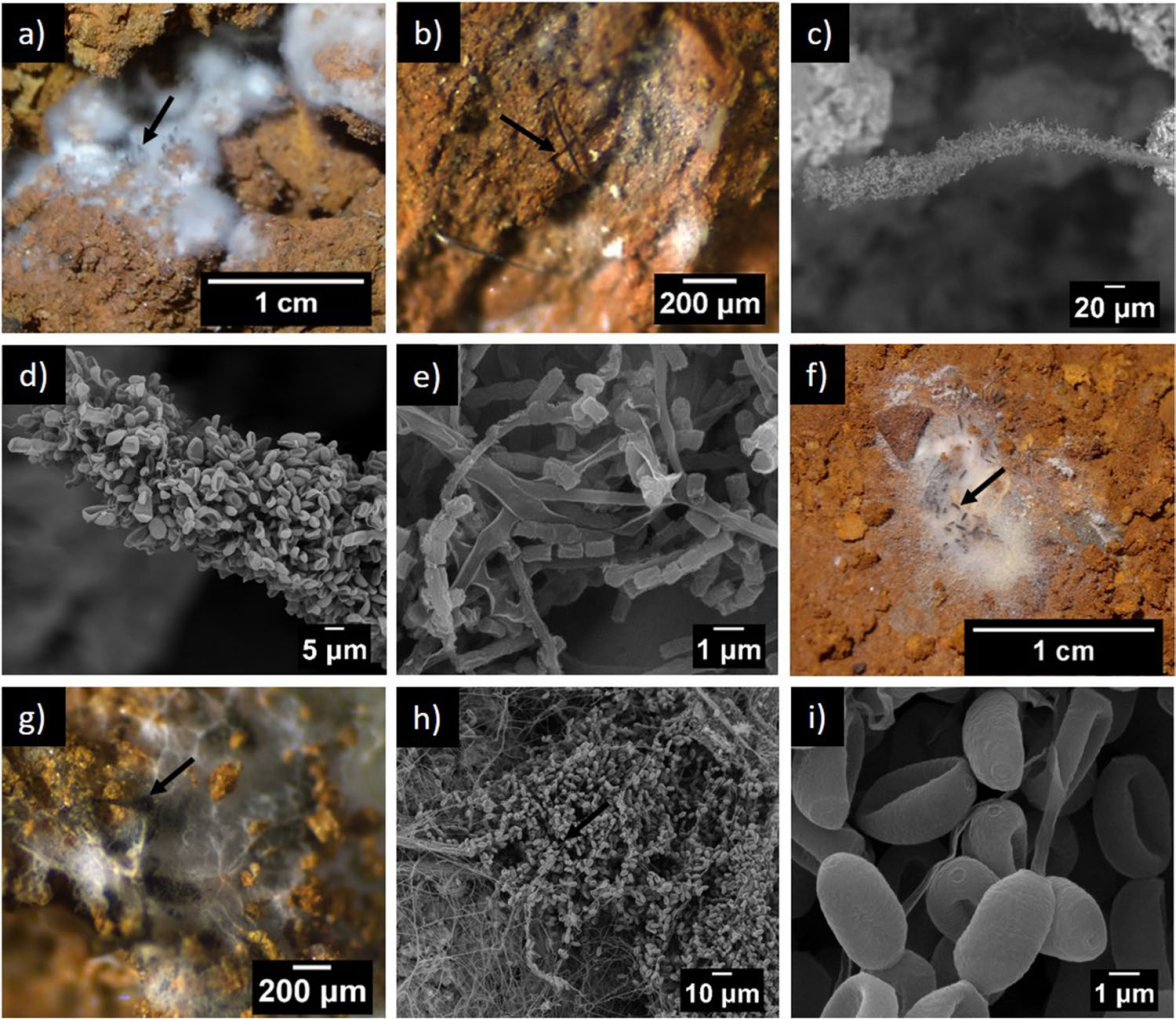
Fungi from P2 (**a**–**d**) and P5 (**f**–**i**) sampling points. **a** Visible synnemata of *Cephalotrichum microsporum* growing through white mycelial colony, **b** synnemata of *C. microsporum* on cave sediment, **c** detail of synnemata, **d** conidia on synnemata tip, **e** arthroconidia, **f**, **g** colony of *Neocosmospora solani* with growing synnemata *of C. microsporum*, **h** a mass of conidia of *C. microsporum*, **i** detail view on conidia of *C. microsporum*. Black arrows point the synnemata of *C. microsporum*

The occurrence and abundance of *Cephalotrichum microsporum* are confirmed in Fig. 2 and in the SEM micrographs obtained from the 2021 samples (Fig. 3). These micrographs mainly depicted the morphological characteristics of the fungus. In fact, the synnemata size is about 500 µm long and the conidia are oval, smooth, and 3.5 × 2 µm, in the ranges reported by Sandoval-Denis et al. [29]. *C. microsporum* appears in a few samples mixed with *N. solani* and other fungi.

### Fungal Identification by NGS

**Fig. 4:**
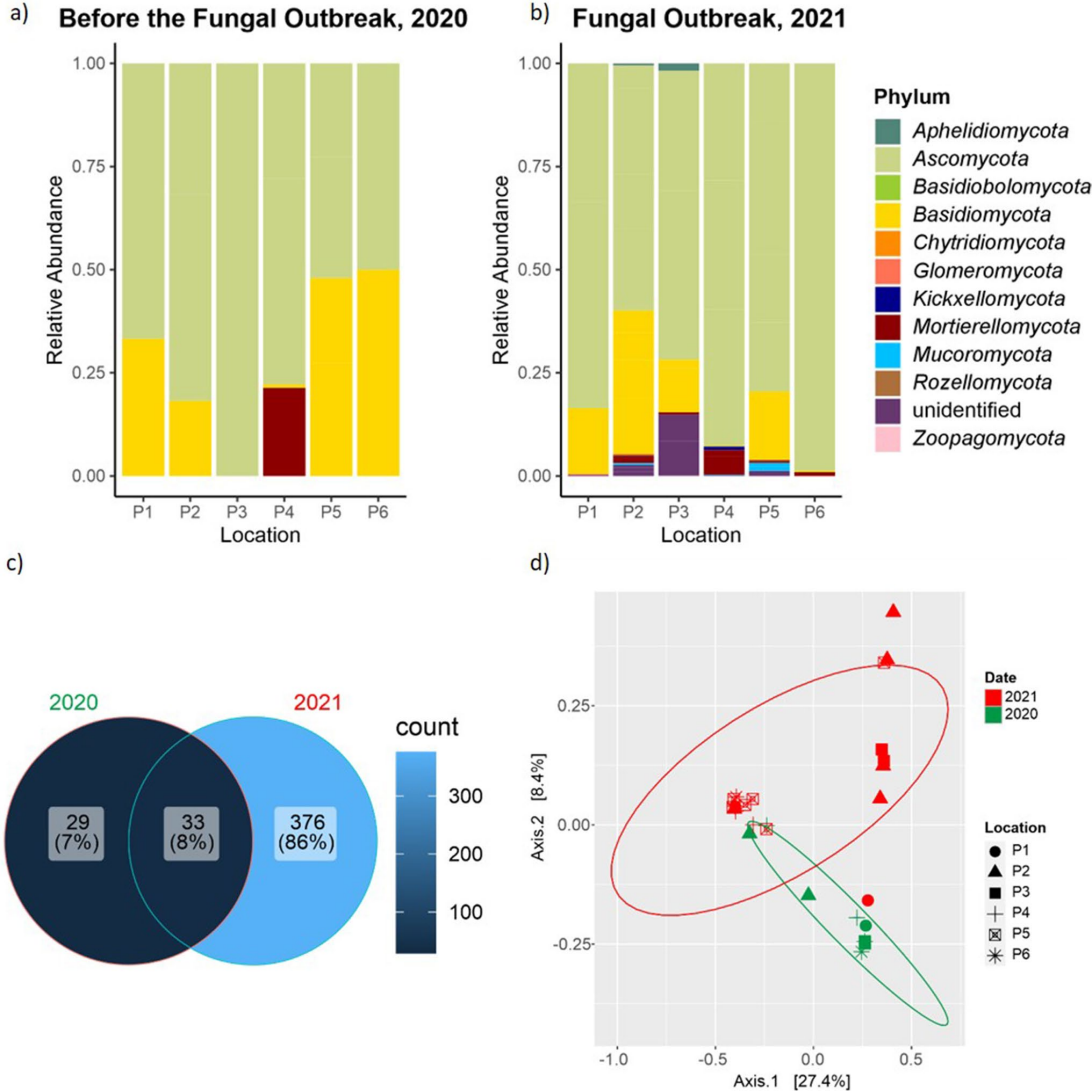
Barplot showing fungal abundance at phylum level in Castañar Cave. **a** Samples from the 2020 campaign, showing no signs of a fungal outbreak. **b** Samples from the 2021 campaign, corresponding to the fungal outbreak. **c** Venn diagram. **d** PCoA analysis

In 2020, in the sampled areas (P1–P6), only fungi belonging to three phyla *Ascomycota*, *Basidiomycota*, and *Mortierellomycota* were identified (Fig. 4a). Samples taken in December 2020 showed a predominance of the phyla *Ascomycota* (50–100%) over *Basidiomycota* (0–50%). The *Mortierellomycota* phylum was found in *Sala Blanca* with abundances greater than 1%.

In 2021, nine phyla were retrieved in all six locations (Fig. 4b), with a significant abundance of *Ascomycota* ranging from 60.78 to 98.79%, followed by *Basidiomycota* with abundances from 0.26 to 35.94%, *Mortierellomycota* from 0.05 to 6%, and *Mucoromycota* from 0 to 1.96%. Other phyla such as *Kickxellomycota*, *Aphelidiomycota*, *Glomeromycota*, *Chytridiomycota*, and *Zoopagomycota* presented abundances below 1% and were scattered distributed in the samples.

The Shannon alpha diversity index provides a comprehensive summary of the structure of the fungal community, considering both the quantity of OTUs and the distribution of their abundance across all samples (Supplementary Figure S1). The sample richness varied from 1 to 175 OTUs, and the sample Shannon index varied from 0 to 4. The highest alpha diversity values in 2020 were detected at the cave entrance (P1) and in 2021 in the samples from the main gallery samples (P2 and P3). The comparison of Shannon diversity between the 2020 and 2021 samples suggests that there may be a significant difference between both sampling dates with higher values during the 2021 fungal outbreak (Mann–Whitney *p* = 0.050). The Venn diagram analysis compares the presence and overlap of OTUs (Fig. 4c). The results showed that in samples from the years 2020 and 2021, 7% of the OTUs were exclusive to 2020, 86% were exclusive to 2021, and 8% were common to both years. The results of the beta diversity analysis indicate a clear distinction between the composition of the fungal community before and after the outbreak (PERMANOVA *p* = 0.017 and ANOSIM *p* = 0.001). The first two components of the PCoA explained about 40% of the variation in the data and the samples can be clustered by date sampling (Fig. 4d).

**Fig. 5:**
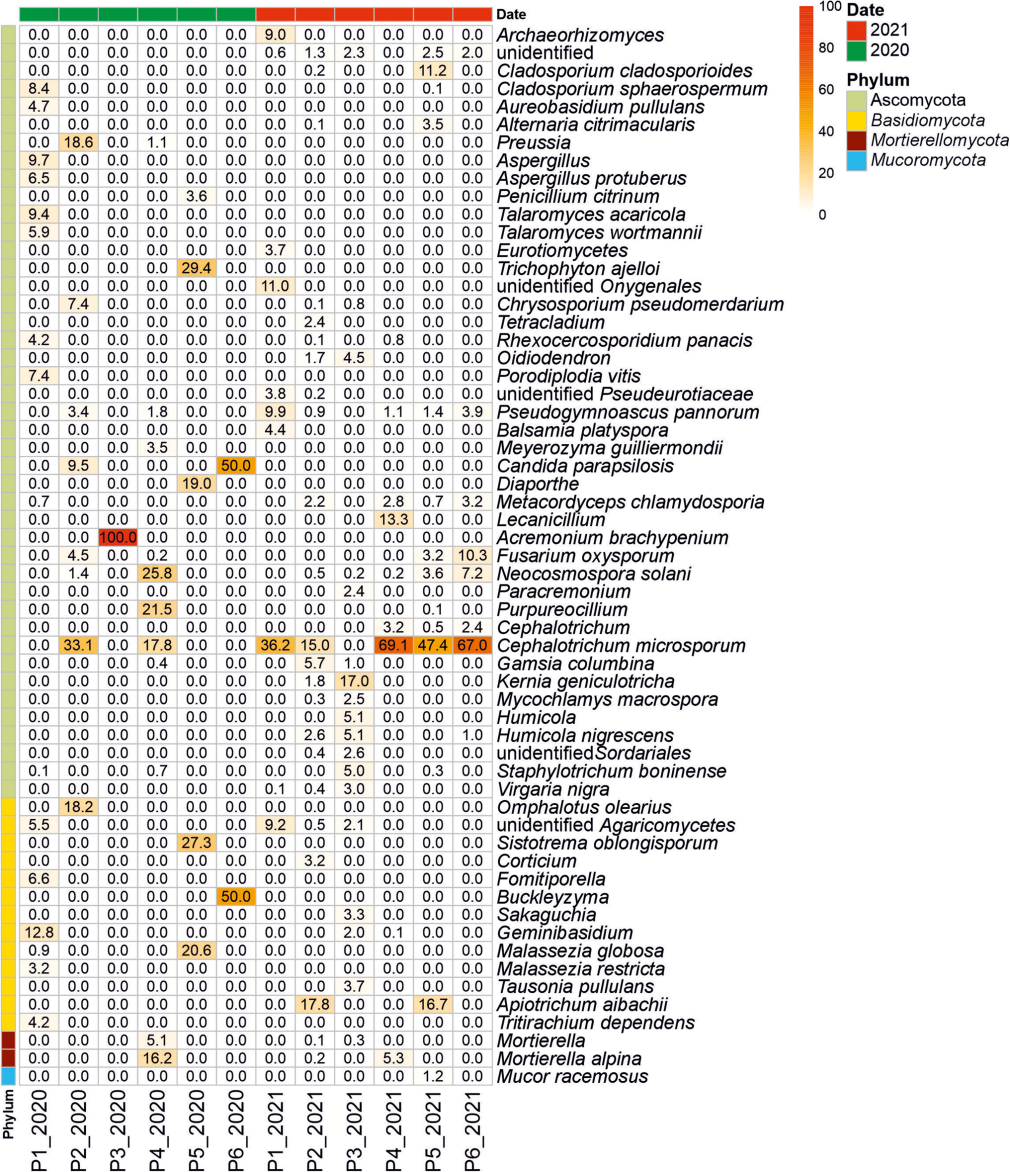
Fungal heatmap in Castañar Cave for the years 2020 (no fungal outbreak) and 2021 (fungal outbreak). The scale bars indicate the relative sequence abundance, with white squares representing the least abundant species and red squares representing the most abundant. The left bars and the legend on the right display the classification at the phylum level. Fungal heatmap displaying the classification of sequences at the smallest taxon level achievable

Figure 5 shows the most abundant fungi identified by NGS in Castañar Cave during the sampling campaigns in 2020 and 2021. In 2020 samplings, 39 taxa with relative abundances > 1% were retrieved in the different halls and galleries with significant abundances of *Ascomycota* (*Acremonium brachypenium*, *Candida parapsilosis*, unidentified *Diaporthe* and *Preussia*, *C. microsporum*, *N. solani*, unidentified *Purpureocillium*, *Trichophyton ajelloi*), some *Basidiomycota* (*Omphalotus olearius*, unidentified *Buckleyzyma*, *Sistotrema oblongisporum*, unidentified *Geminibasidium*, *Malassezia globosa*), and *Mortierellomycota* (*Mortierella alpina*).

In 2021, a significantly larger number of taxa were obtained compared to the 2020 sampling: approximately 64 taxa with relative abundances > 1% and 344 taxa with relative abundances < 1% (Supplementary Table S4).

NGS data from Castañar Cave sediments obtained 1 year before the second outbreak (2020) and 2 days after the second outbreak (2021) revealed that a few fungal species extended their distribution and reached generally higher relative abundances with respect to 2020. This was the case of *C. microsporum*, *Lecanicillium* sp., *F. oxysporum*, *N. solani*, *Pseudogymnoascus pannorum*, *Staphylotrichum boninense*, *Gamsia simplex*, and *M. alpina.*

In the 2021 outbreak, the most abundant fungus in Castañar Cave was *C. microsporum*, which was distributed in all the cave halls and passages with conspicuous hyphal mats (Figs. 2 and 3) and relative abundances between 15.0 and 69.1%, except in P3.

Other abundant fungi with relative abundances above 10% were *Kernia geniculotricha* (17.0% in P3, *Galeria Principal*), *Apiotrichum laibachii* (17.8% in P2, *Galeria Principal*-vomit and 16.7% in P5, a narrow passage, the connection from *Sala Nevada* to *Sala del Jardin*), *Lecanicillium* sp. (13.3% in P4, *Sala Nevada*), *Cladosporium cladosporioides* (11.2% in P5), unidentified *Onygenales* (11.0% in P1, *Sala de Entrada*), *F. oxysporum* (10.3% in P6, *Sala del Jardin*).

Relative abundances between 5 and 10% were allocated to *P. pannorum* (9.9% in P1), unidentified *Agaricomycetes* (9.2% in P1), *Archaeorhizomyces* sp. (9.0% in P1), *Fusarium* sp. (6.9% in P6), *Humicola nigrescens* (5.1% in P3), *S. boninense* (5.0% in P3), *Humicola* sp. (5.1% in P3), *G. simplex* (5.7% in P2), *Oidiodendron* sp. (4.5% in P3), and *M. alpina* (5.3 in P4).

### Fungal Identification by Culture

**Fig. 6:**
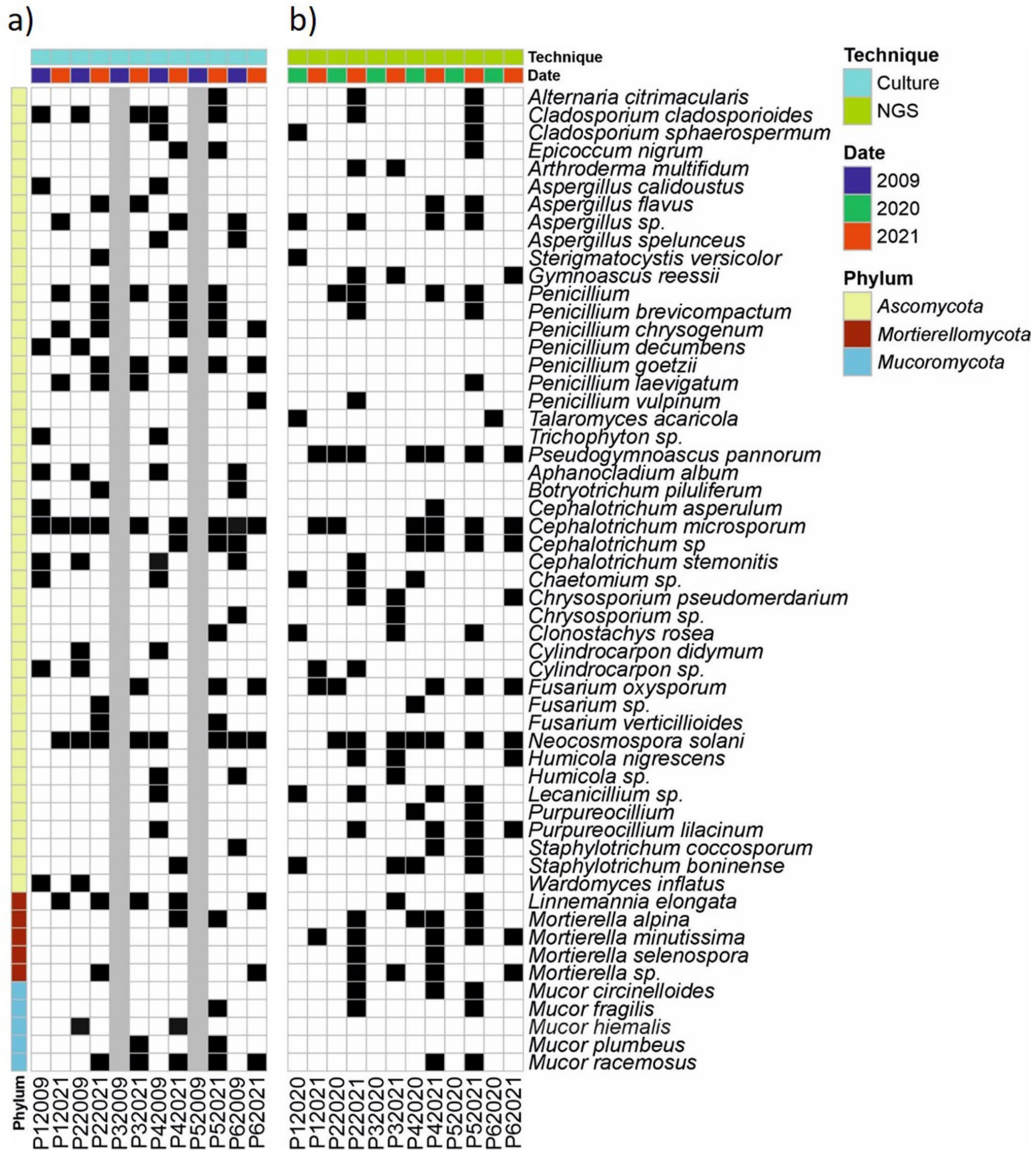
Presence-absence chart comparing samples from **a** culture collections from the 2009 and 2021 campaigns. **b** NGS samples from 2021. Presence: black, absence: white, NA: gray

In 2009, 36 fungal species were identified from culture isolates and 46 species in 2021. Data from fungi isolated in the sediment samplings from Castañar Cave carried out 5 months after the first outbreak (2009) and 2 days after the second outbreak (2021) revealed that some fungal species were persistent during the time (Fig. 6 and Supplementary Tables S1-2). This was the case for *C. microsporum*, *N. solani*, *C. cladosporioides*, and *Mucor hiemalis*. However, the abundance of these fungi was quite different. In general, all these fungi increased in 2021 compared to 2009. *C. cladosporioides* and *M. hiemalis* presented similar and low abundances in both samplings. Of interest was the high number of identified species of *Aspergillus* and *Penicillium*; however, in both years the species were different.

The genus *Cephalotrichum* was represented by the species *C. microsporum*, *C. stemonitis*, and *C. asperulum* in 2009 and by *C. microsporum* and *C. nanum* in 2021. The genus *Mucor* was represented by *Mucor hiemalis* f. *horticola* in 2009 and by *M. racemosus* f. *racemosus*, *M. hiemalis*, and *M. plumbeus* in 2021*.* In 2021, other *Fusarium* were identified, including *F. oxysporum*, *F. verticillioides*, and *F. brachygibbosum*. Other fungi of interest were members of the Mortierellaceae family, *M. alpina*, *Mortierella* sp., and *Linnemannia elongata* (= *Mortierella elongata*), only retrieved in 2021.

Of interest was the significant occurrence in 2009 of entomopathogenic fungi, namely, *Aphanocladium album*, *Amphichorda felina*, and *Purpureocillium lilacinum*, in most of the halls. These fungi were not present in 2021 although other entomopathogenic fungi such as *Fusarium verticillioides*, isolated from several samples from P5 (the connection from *Sala Nevada* to *Sala del Jardin*), and *Marquandomyces marquandii*, *Clonostachys rosea*, *Trichoderma harzianum*, and *Albifimbria verrucaria* were retrieved. This last fungus was especially abundant in the sample from P2.

### Functional Assignment by FUNGuild

**Fig. 7:**
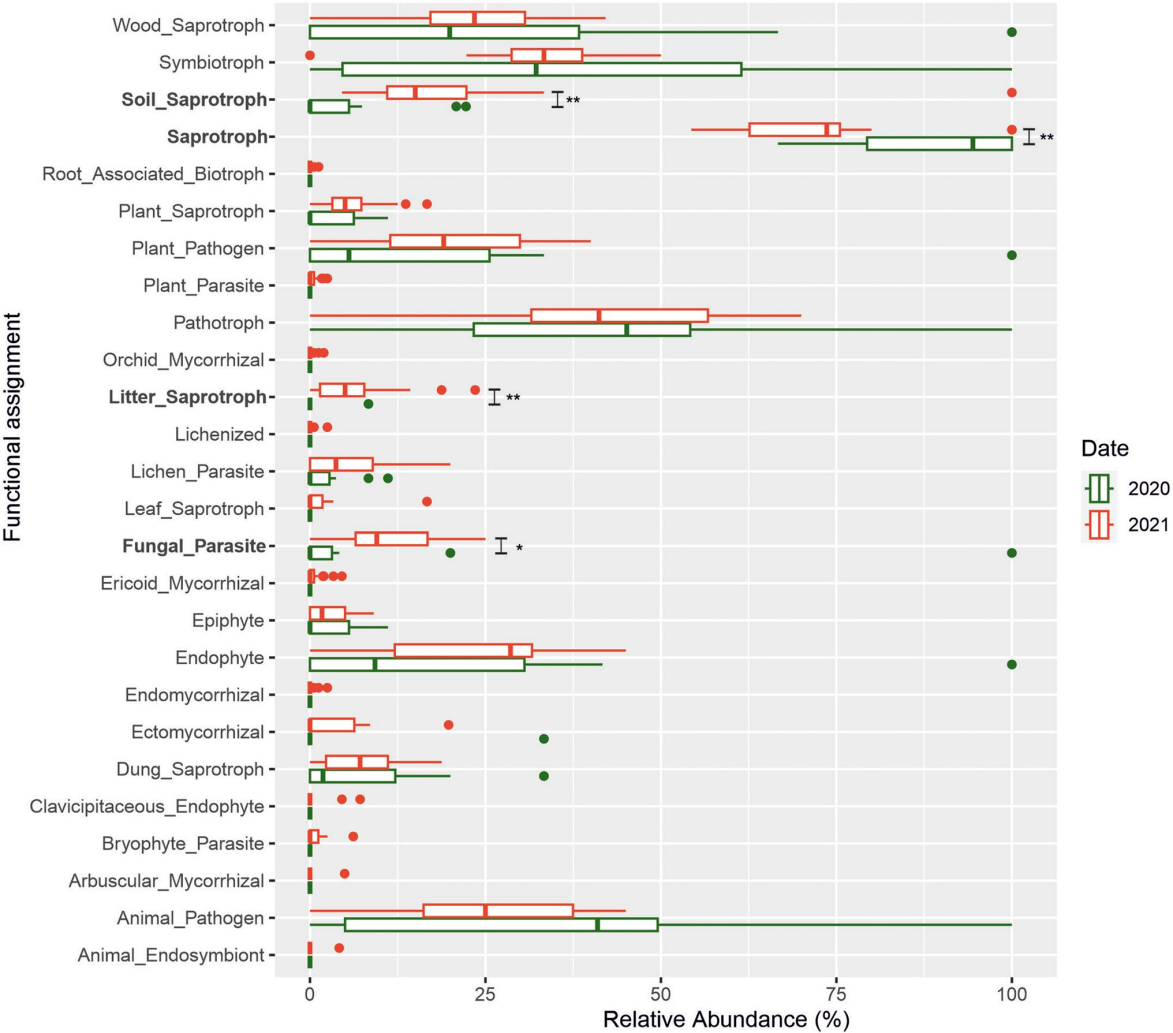
Boxplot showing the frequencies of assigned trophic functions before (2020) and after the outbreak (2021), according to FUNGuild analysis. *Significant difference between the 2020 and 2021 communities (*p* < 0.05, Mann–Whitney test)

The FUNGuild database was used to classify the ecological function of fungi detected in the sediments of Castañar Cave (Fig. 7, Supplementary Tables S5 and S6). There were 437 OTUs obtained from the NGS in 2020 and 2021, which were classified into six ecological function groups according to the trophic mode (Supplementary Table S6) and 25 ecological function groups according to the guild mode (Fig. 7). In general, the fungi detected by NGS in Castañar Cave were classified as undefined saprotrophs according to the trophic mode, representing 28.83% of the total OTUs, while the pathotroph-saprotroph-symbiotroph were the most common fungi detected by culture techniques, representing 24.32% of the total fungal isolates (Supplementary Table S5). The results by guild mode indicated that the most abundant OTUs were classified as undefined saprotroph (38.44%), followed by wood saprotroph (26.77%), plant pathogen (22.65%), and animal pathogen (18.76%). Fungal isolates were classified as plant pathogen (41.89%), endophyte (40.54%), undefined saprotroph (37.84%), and animal pathogen (35.14%). By comparing the NGS results from 2020 and 2021, it was observed that the percentage of undefined saprotrophs was significantly higher in 2020, while during the second outbreak, a higher frequency of soil saprotrophs, litter saprotrophs, and fungal parasites was detected. Although no significant differences were obtained, a higher percentage of plant saprotrophs, plant pathogens, lichen parasites, endophytes, and ectomycorrhizae is evident, as shown in Fig. 7.

## Discussion

### Fungal Outbreak History and Present-Day Situation

In August 2008, a visitor vomited in the *Galeria Principal*, about 50 m from the entrance, and 2 days later, a fungal outbreak was observed, composed of *M. circinelloides* and *F. solani* (= *N. solani*) [6]. Both fungi were noticed in the second fungal outbreak of 2021. *N. solani* was highly abundant and was detected using both NGS and culture approache*s*, while *M. circinelloides* appeared with abundances < 1% and was only detected in 2021 by NGS. *M. circinelloides* is considered an opportunistic human pathogen [30] and was classified as an animal pathogen-plant pathogen according to FUNGuild (Supplementary Table S5). In contrast, *Fusarium* species are among the economically significant plant pathogens and are more metabolically diverse, which may explain their persistence in the cave over the years [31].

The history of samplings in Castañar Cave shows that only three fungi retrieved in the first fungal outbreak in 2008 were also found in the following 2009 and 2020 campaigns, as well as during the second outbreak in 2021: *N. solani*, *F. oxysporum*, and *M. alpina* [6, 17].

In a previous report, we stated that *N. solani* was a permanent dweller of Castañar Cave because this fungus was present in all samplings since 2008 [17]. Some studies confirmed that this is a widespread fungus in nature and is considered a natural dweller in caves and subterranean environments [3]. The genus *Neocosmospora* includes saprotrophs, and two cases of outbreaks in caves related to anthropogenic impact in which this fungus is involved have been previously reported [3, 6].

*F. oxysporum* is commonly retrieved in caves worldwide, where it has been reported to be an entomopathogenic fungus [32]. This fungus has also been recorded in environments with high radiation such as Chernobyl, suggesting that it can persist in oligotrophic environments in high radiation conditions for long periods of time [33].

*Mortierella* spp. are considered important members of the soil core microbial community [34]. Ten species of *Mortierella* were retrieved in Castañar Cave, but only two of them (*M. alpina* and *Mortierella* sp.) with relative abundances > 1%. *M. alpina* is common in soils and cave sediments [35]. *M. alpina* and other *Mortierella* species were abundant in bat guano in caves [36]. In Castañar Cave, no bats were reported, but rodents and geckos and their feces were abundant.

Martin-Pozas et al. [17] stated that the abundance of *C. microsporum* represented a potential risk for the conservation of Castañar Cave and that this and other abundant taxa should be controlled. Unfortunately, this was confirmed in 2021 with a second outbreak of *C. microsporum* based on data from NGS and fungal isolates from cultures of sediments (Fig. 6). In fact, although no apparent colonization of the sediments by fungal mycelia was observed in 2020, in 2021 *C. microsporum* experienced a sudden explosive growth after an anthropogenic impact on the cave (Figs. 1, 2 and 3). Members of the genus *Cephalotrichum* have a saprophytic behavior and were often retrieved from animal dung, soils, and caves [35, 37]. In Castañar Cave, other species of *Cephalotrichum*, such as *C. stemonitis*, *C. asperulum*, and *C. nanum*, were identified at various sampling points using culture techniques. However, when NGS was used, high relative abundances were only detected for *C. microsporum* and unassigned *Cephalotrichum*. Jiang et al. [37] indicated that many cave isolates of the *Cephalotrichum* genus are oligotrophic and have the ability to grow in carbon free media. This could suggest that *C. microsporum* persists in Castañar Cave waiting for an abundant source of organic carbon source to boost and colonize the cave sediments under favorable ecological conditions.

NGS results revealed a higher fungal diversity and a difference in the composition of the fungal community after the second outbreak (Fig. 4). In fact, an enrichment in nine phyla in 2021 with respect to that of 2020 (only three phyla) was observed. The functional assignment of the fungi retrieved in Castañar Cave denotes a clear evidence of the impact of the continuous entry of workers into the cave throughout the 6 months of installation of the steel walkway. The increasing abundance of soil-, plant-, and litter-saprotrophs, lichen-parasites, endophytes, and ectomycorrhizes, in 2021 with respect to 2020, reinforces the exogenous input of plant debris from the top soil that caused the outbreak. This is consistent with the predominance of *Ascomycota* over *Basidiomycota*, which dominates in the litter decomposing community [38].

In addition to *C. microsporum*, *F. oxysporum*, and *N. solani*, other abundant *Ascomycota* appeared during different periods in Castañar Cave. This is consistent with other studies in which saprophytic *Ascomycota* dominated the fungal community in caves impacted by anthropogenic activities [11, 35].

In general, persistent *Ascomycota* in Castañar Cave can be broadly grouped in two ecological groups: entomopathogenic (e.g., *Lecanicillium* sp., *C. cladosporioides*, and *F. oxysporum*) and soil fungi (*Gamsia simplex*, *P. pannorum*, *Staphylotrichum boninense*, *C. microsporum*, *N. solani*).

In Castañar Cave,* C. cladosporioides* was isolated at three sampling points in 2009 and at five sampling points in 2021. This fungus was not detected in 2020 with NGS but was detected at two points of the cavity in 2021 (Fig. 6). *C. cladosporioides*, an entomopathogenic fungus [29], is commonly retrieved in caves worldwide [2, 11, 39].

*Lecanicillium* sp., *P. pannorum*, and *S. boninense* were detected by NGS in 2020 and 2021 samples (Fig. 6). The genus *Lecanicillium* is largely known to comprise insect pathogens [40], although members of this group also parasitize other arthropods, fungi, nematodes, and plants [41]. Species of this genus were retrieved in European caves, most of them with abundance of arthropods [39].

*P. pannorum*, a keratinolytic fungus, has a broad geographic distribution in caves all over the world and is common in sediments and cave air [35, 42]. *S. boninense* was probably isolated in 2009 using the bait technique from three sites, but was identified according to its morphology as *S. coccosporum* (Supplementary Table S2), the only known species at that time; *S. boninense* was described later [43]. Therefore, it seems feasible that *S. boninense* was present in the cave since the first outbreak.

Unlike *Ascomycota*, we found significant variability in the occurrence of *Basidiomycota* species in Castañar Cave during the sampling periods of 2020 and 2021. In both periods, only a few unidentified fungi of the class *Agaricales* were detected, as well as *S. oblongisporum*, *Geminibasidium*, and *Tritirachium dependens*. Furthermore, in all these cases, their abundance was low during the outbreak. Special mention merits* A. laibachii* (= *Trichosporon laibachii*), the most abundant basidiomycete fungus in the outbreak samples. *Apiotrichum* is a widespread genus of soil yeasts, found in all types of biotopes [44]. The 2020 samples showed a strong relative abundance of yeasts, namely, *Candida*, *Buckleyzyma*, and *Malassezia* species in the cave and *Solicoccozyma* species in the soil outside the cave. In 2021, these yeasts were replaced by *A. laibachii* and with lower relative abundances by *Sakaguchia* and *Tausonia* (Fig. 3, Supplementary Table S4). According to Yurkov [44], this can be explained by the fact that basidiomycetous yeasts are usually abundant in soils but these transient species are quickly eliminated (outcompeted) in their niches, likely in this case by the dominant *Cephalotrichum*. In fact, these three yeasts appeared in samples with lower relative abundances of *Cephalotrichum*.

Overall, the microbiological studies conducted in Castañar Cave showed that both fungal outbreaks were caused by human activities. The first was related to the opportunistic fungus *M. circinelloides*, which developed from a visitor’s vomit, and the most recent fungal outbreak was related to *C. microsporum* and other plant- and soil-saprotrophs due to organic matter introduced during the works for the installation of a steel grating walkway.

### Contrasting Cultivation and NGS Techniques

The inability of culture-dependent and independent methods to detect the same taxa has been widely reported [45]. In fact, culture-dependent techniques lead to an overestimation of the spore-forming microorganisms, and the apparent abundances can be explained by their rapid growth in the plates.

The culture collection provided a higher number of *Aspergillus* spp. and *Penicillium* spp., as well as a few more genera (Supplementary Tables S2 and S3), which were not evident in the NGS library (Supplementary Table S4). Five *Aspergillus* spp. and 12 *Penicillium* spp. were retrieved from cultures and five and seven from NGS, although with low relative abundances, but only *Aspergillus flavus* and *Penicillium brevicompactum* were shared in both cases, the rest of the species were different in both approaches. The high occurrence of isolates from these two genera is common in sediments collected in other Spanish caves, as well as in aerobiological studies [39, 42]. The case of *A. verrucaria* (= *Myrothecium verrucaria*) highly detected in *Galeria Principal* (Supplementary Table S3), but not retrieved by NGS is also notable. *A. verrucaria* is a pathogenic soil and plant fungus identified in caves [46].

The detectability of NGS was obviously higher than that of traditional culture methods. This was demonstrated in the 10 different species of *Mortierella* in the NGS against four isolated *Mortierella* spp. (including *L. elongata*). In Castañar Cave, NGS allowed the recovery of 243 identified fungal species, in addition to unidentified/unassigned species, compared to 33 species identified by culture (Supplementary Table S4).

Some abundant fungi such as *Kernia geniculotricha* were only identified by NGS in 2021. The genus *Kernia* comprises species commonly isolated from animal dung [47]. *Kernia geniculotricha*, the synonym of *K. nitida*, was isolated from rabbit dung [48]. Guarro Artigas [49] isolated some strains of *K. nitida* from the dung of sheep and goats. To our knowledge, *K. geniculotricha* has not been reported in cave environments. In the case of Castañar Cave, it could be related to goat dung, abundant in the top soil and likely introduced by the workers in their boots.

It should be noted that only a few fungi were shared in both culture-dependent and culture-independent approaches if we compare sequences with relative abundance > 1% with isolates (Fig. 6). However, both methods have proven equally effective in identifying the most prevalent fungi usually located in the cave: *C. microsporum*, *N. solani*, *F. oxysporum*, and *M. alpina*.

In summary, the results of both approaches showed that ascomycetes members were dominant in the sediments, but the culture-dependent approach resulted in a lower fungal diversity.

### Outbreak Control

A critical point in the conservation of show caves is the control of organic matter and inorganic materials introduced into the cave [9, 50]. In our previous report on the first fungal outbreak in Castañar Cave [6] we stated: “*Even with controlled visits delicate trophic relationships that exist in a cave can be disturbed. Thus, changes produced by management activities, accidental input of organic matter, introduction of fresh rocks and other inorganic substrata, *etc*. produce an imbalance in the cave, which is rapidly colonized by those members of the microbial community that are most active. These successful colonizers are able to cope with a variety of carbon sources, including rat bait and faeces*.” These comments have unfortunately come true and have been reflected in the second fungal outbreak, which evidenced the fragility of show caves and proved that any type of visits can compromise cave conservation.

The different cleaning chemicals and bioremediation approaches used in show caves have been previously discussed [9]. Since there is no ideal solution, it is better to rely on preventive conservation and early detection of microbial outbreaks. If necessary, the least harmful bioremediation methods should be adopted. The 2008 fungal outbreak was controlled by means of a careful mechanical removal of the sediments contaminated by fungal growth and a subsequent sterilization of the remaining sediments with commercial hydrogen peroxide. Hydrogen peroxide degrades organic matter and the originating products (water, oxygen, and CO_2_) are harmless to the cave environment [6]. This treatment was effective and no further fungal growth could be observed in the sediments during the following 13 years. A similar but stronger treatment was adopted for the 2021 outbreak. In this case, the sediments with fungal overgrowth were covered with plastic bags to avoid the dispersion of the spores, subsequently excavated at depth and mechanically removed. The removed area was soaked with abundant 30% v/v hydrogen peroxide. In addition, the surrounding area up to 1 m in diameter was sprayed with 30% v/v hydrogen peroxide.

After the 2008 fungal outbreak, preventive strategies designed for cave conservation, including frequent surveys, allowed for rapid warning and immediate action during the 2021 fungal outbreak. These measures proved crucial in adopting swift control and cleaning measures. No further fungal growth was observed in the following 2 years. In the medium and long term, frequent cave surveys can provide early warnings of potentially dangerous situations. In this case, surveys conducted by guides and cave staff (from the Environment Department of the Extremadura Autonomous Government), in collaboration with geomicrobiologists, allowed the identification of the outbreak’s origin and the proposal of the most appropriate treatments. For Castañar Cave, it is recommended to continue with the established protocol after the first outbreak. Furthermore, based on the results obtained in this study, it is strongly advised to minimize any construction work that involves the movement of materials and sediments within the cave.

## Conclusions

Castañar Cave experienced an anthropogenic impact as a consequence of the installation of a steel grating walkway on the floor to prevent visitors from walking in the sediments. This promoted that the ecological status in Castañar Cave changed in a 1-year lapse (2020–2021), since nutrients are a major determinant factor in caves and the input of exogenous anthropogenic carbon changed the composition of the fungal community.

The work started in July and ended in December 2021. The second fungal outbreak dated November 2021, clearly indicating that carbon input to sediments occurred during these works and was a key determinant of the boosting of fungal communities in Castañar Cave. Data show that the most abundant fungi in the outbreak were already inside the cave and increased the growth and colonization of sediments after a drastic ecological change.

The different nature of organic carbon in the 2008 (human vomit) and 2021 (organic matter introduced by workers) outbreaks of Castañar Cave justifies the diverse fungal communities. In 2008, the most abundant fungus was *M. circinelloides*, while in the 2021 outbreak was *C. microsporum*. This study demonstrates that the fungi that dwell in the cave, and are detected in the 2020 NGS approach, can be reactivated at any time and induced to a luxurious growth and outbreak with a slight change in the environmental conditions. Some other fungi were introduced into the cave during the walkway works.

Fungal outbreaks in Castañar and other caves demonstrate the fragility of these confined environments and their negative reaction to anthropogenic disturbances. Here, we demonstrate that not only tourist visits but any human action, including management activities aimed at protecting the cave, can provoke disturbances and ecological changes in the delicate trophic status of cave fungi.

These results suggest that special attention is needed in show caves to avoid any entry of organic material and to maintain the environmental conditions as close as possible to the natural state of the cave. Coordinated and collaborative work between cave managers and specialized research teams focusing on underground ecosystems is essential for the design and implementation of preventive conservation measures and their application to existing issues.

### Supplementary Information

Below is the link to the electronic supplementary material.

Supplementary file1 (PDF 1789 KB)

## Data Availability

The gene sequences and accompanying metadata were deposited in the Sequence Read Archive (SRA) of NCBI under the projects number PRJNA802044 and PRJNA1045599.
