# Supplementary material for: A Second Fungal Outbreak in Castañar Cave, Spain, Discloses the Fragility of Subsurface Ecosystems
Source: Microb Ecol. 2024 Mar 20;87:53. doi: 10.1007/s00248-024-02367-2 (PMC10954929; doi:10.1007/s00248-024-02367-2)
Supplement: Supplementary file 1 [file 248_2024_2367_MOESM1_ESM.pdf]

SUPPLEMENTARY INFORMATION

A SECOND FUNGAL OUTBREAK IN CASTAÑAR CAVE, SPAIN, DISCLOSES THE FRAGILITY OF SUBSURFACE ECOSYSTEMS

Tamara Martin-Pozas[a,b], Alena Nováková[c], Valme Jurado[d], Soledad Cuezva[e], Angel Fernandez-Cortes[b], Cesareo Saiz-Jimenez[d], Sergio Sanchez-Moral[a]

[a] Museo Nacional de Ciencias Naturales, MNCN-CSIC, 28006 Madrid, Spain

[b] Departamento de Biologia y Geologia, Universidad de Almeria, 04120 Almeria, Spain

[c] Laboratory of Fungal Genetics and Metabolism, Institute of Microbiology of the CAS, Prague, Czech Republic

[d] Instituto de Recursos Naturales y Agrobiologia, IRNAS-CSIC, 41012 Sevilla, Spain

[e] Spanish Geological Survey, IGME-CSIC, 28003 Madrid, Spain

Table S1. Sample description.

**Sample**	**Sampling point**	**Cave Location**	**Type of sample**	**Sampling Date (Method)**
F1-1	P1	*Sala de entrada*	Mycelial fungi on sediment	2021 (Culture and NGS)
F1-2	P1	*Sala de entrada*	Mycelial fungi on sediment	2021 (Culture and NGS)
F2-1	P2	*Galeria Principal* (Vomit area)	Mycelial fungi on sediment	2021 (Culture and NGS)
F2Fc-1	P2	*Galeria Principal* (Vomit area)	Mycelial fungi on sediment	2021 (Culture and NGS)
F2Fc-2	P2	*Galeria Principal* (Vomit area)	Mycelial fungi on sediment	2021 (Culture and NGS)
F3-1	P2	*Galeria Principal* (Vomit area)	Mycelial fungi on sediment	2021 (Culture and NGS)
F3-p	P2	*Galeria Principal* (Vomit area)	Mycelial fungi on sediment	2021 (Culture and NGS)
P3-1	P3	*Galeria Principal*	Mycelial fungi on sediment	2021 (Culture and NGS)
P3-2	P3	*Galeria Principal*	Mycelial fungi on sediment	2021 (Culture and NGS)
F4-1	P4	*Sala Nevada*	Mycelial fungi on sediment	2021 (Culture and NGS)
F4-2	P4	*Sala Nevada*	Mycelial fungi on sediment	2021 (Culture and NGS)
F4-3	P4	*Sala Nevada*	Mycelial fungi on sediment	2021 (Culture and NGS)
F5-1	P5	Narrow passage, a connection from *Sala Nevada* to *Sala del Jardin*	Mycelial fungi on sediment	2021 (Culture and NGS)
F5-2	P5	Narrow passage, a connection from *Sala Nevada* to *Sala del Jardin*	Mycelial fungi on sediment	2021 (Culture and NGS)
F5-CIBOR	P5	Narrow passage, a connection from *Sala Nevada* to *Sala del Jardin*	Mycelial fungi on sediment	2021 (Culture and NGS)
F51-1	P5	Narrow passage, a connection from *Sala Nevada* to *Sala del Jardin*	Mycelial fungi on sediment	2021 (Culture and NGS)
F52-1	P5	Narrow passage, a connection from *Sala Nevada* to *Sala del Jardin*	Mycelial fungi on sediment	2021 (Culture and NGS)
F52-2	P5	Narrow passage, a connection from *Sala Nevada* to *Sala del Jardin*	Mycelial fungi on sediment	2021 (Culture and NGS)
F6-1	P6	*Sala del Jardin*	Mycelial fungi on sediment	2021 (Culture and NGS)
G1	P1	*Sala de Entrada*	Sediment without visible colonization	2009 (Culture), 2020 (NGS)
C2	P2	*Galeria Principal* (Vomit area)	Sediment without visible colonization	2009 (Culture), 2020 (NGS)
CC2	P2	*Galeria Principal* (Vomit area)	Sediment without visible colonization	2009 (Culture), 2020 (NGS)
CC3	P3	*Galeria Principal*	Sediment without visible colonization	2020 (NGS)
C4	P4	*Sala Nevada*	Sediment without visible colonization	2009 (Culture), 2020 (NGS)
CC4	P4	*Sala Nevada*	Sediment without visible colonization	2009 (Culture), 2020 (NGS)
C5	P5	Narrow passage, a connection from *Sala Nevada* to *Sala del Jardin*	Sediment without visible colonization	2020 (NGS)
CC5	P5	Narrow passage, a connection from *Sala Nevada* to *Sala del Jardin*	Sediment without visible colonization	2020 (NGS)
C6	P6	*Sala del Jardin*	Sediment without visible colonization	2009 (Culture), 2020 (NGS)
CC6	P6	*Sala del Jardin*	Sediment without visible colonization	2009 (Culture), 2020 (NGS)

Table S2. Fungi isolated from the sediments of Castañar Cave in 2009.

**Sampling Point**		**Sediment/soil**	**Sediment – isolation by Keratin-bait technique**	**Sediment- isolation by Cellulose-bait technique**	**Cave ceillings (sediment)**
**P1**	Sala de entrada	*Aphanocladium album*<br>*Aspergillus calidoustus*<br>*Cadophora fastigiata*<br>*Cephalotrichum microsporum*<br>*Cephalotrichum stemonitis*<br>*Cladosporium cladosporioides* group<br>*Cylindrocarpon* sp.<br>*Hormiactis fimicola*<br>*Penicillium decumbens*<br>*Penicillium expansum*<br>*Penicillium glabrum*<br>*Penicillium simplicissimum*<br>*Wardomyces inflatus*	*Aspergillus calidoustus*<br>*Cephalotrichum asperulum*<br>*Cephalotrichum microsporum*<br>*Cephalotrichum stemonitis*<br>*Chaetomium* sp.<br>*Trichophyton* sp.	-	*Cladophialophora sp.*
**P2**	Galeria Principal (Vomit area)	*Amphichorda felina*<br>*Aphanocladium album*<br>*Cladosporium cladosporioides* group<br>*Cladosporium herbarum* group<br>*Cylindrocarpon* sp.<br>*Penicillium decumbens*<br>*Phoma* sp.<br>*Wardomyces inflatus*<br>undetermined species of *Basidiomycetes*	*Cephalotrichum stemonitis*<br>*Fusisporium didymum*	*Cephalotrichum microsporum*<br>*Purpureocillium lilacinum*	*Amphichorda felina*<br>*Aspergillus fumigatus*<br>*Fusisporium didymum*<br>*Mucor hiemalis* f. *horticola*<br>*Penicillium decumbens*
**P4**	Sala Nevada	*Arthroderma terrestre*<br>*Aspergillus aureolatus*<br>*Aspergillus calidoustus*<br>*Aspergillus fumigatus*<br>*Cephalotrichum stemonitis*<br>*Chrysosporium sp.*<br>*Cladosporium cladosporioides group*	*Aspergillus spelunceus*<br>*Botryotrichum piluliferum*<br>*Cladosporium sphaerospermum*<br>*Fusisporium didymum*<br>*Trichophyton* sp.	*Aphanocladium album*<br>*Neocosmospora solani*<br>*Humicola* sp.	-
**P6**	El Jardín	*Aphanocladium album*<br>*Aspergillus citocrescens*<br>*Chrysosporium sp.*<br>*Purpureocillium lilacinum*<br>*Spiniger meineckellus/Heterobasidion parviporum*	*Aspergillus spelunceus*<br>*Aspergillus* sp. (sect. *Nidulantes*)<br>*Neocosmospora solani*<br>*Humicola* sp.<br>*Penicillium cyclopium*<br>*Staphylotrichum coccosporum*	*Botryotrichum piluliferum*<br>*Cephalotrichum microsporum*<br>*Cephalotrichum stemonitis*<br>*Staphylotrichum coccosporum*	-

Table S3. Fungi isolated from the sediments of Castañar Cave in 2021. **Bold** – plentifully occurrence on Petri dishes

Sampling Point		Identification according to morphological properties	PCR fingerprinting
**P1**	*Sala de Entrada*	*Cephalotrichum microsporum*	*Aspergillus allahabadii*<br>*Aspergillus*<br>*Linnemannia elongata*<br>*Neocosmospora solani*<br>*Penicillium chrysogenum*<br>*Penicillium laevigatum*<br>*Penicillium*<br>*Talaromyces (wortmanii/rogersiae)*<br>*Trichoderma virens*
**P2**	*Galeria Principal* (Vomit area)	*Mucor racemosus f. racemosus*<br>*Fusarium sp.*<br>*Cephalotrichum microsporum*	***Albifimbria verrucaria***<br>*Aspergillus flavus*<br>*Aspergillus versicolor*<br>*Cephalotrichum nanum*<br>*Cladosporium cladosporioides*<br>*Cladosporium delicatulum*<br>*Cladosporium pseudocladosporioides*<br>*Neocosmospora solani*<br>*Fusarium verticillioides*<br>*Marquandomyces marquandii*<br>*Linnemannia elongata*<br>*Mortierella* sp.<br>*Penicillium brevicompactum*<br>*Penicillium chrysogenum*<br>*Penicillium goetzii*<br>*Penicillium laevigatum*<br>*Penicillium rubens*<br>*Penicillium*<br>*Trichocladium uniseriatum*
**P3**	*Galeria Principal*	*Cephalotrichum microsporum*<br>*Mucor plumbeus*<br>*Mucor racemosus* f. *racemosus*	*Aspergillus flavus*<br>*Cladosporium cladosporioides*<br>*Fusarium oxysporum*<br>*Neocosmospora solani*<br>*Linnemannia elongata*<br>*Penicillium goetzii*<br>*Penicillium laevigatum*<br>*Penicillium*
**P4**	*Sala Nevada*	*Cephalotrichum microsporum*	Aspergillus<br>*Cephalotrichum nanum*<br>*Cladosporium aggregatocicatricatum*<br>*Cladosporium cladosporioides*<br>*Cladosporium delicatulum*<br>*Epicoccum nigrum*<br>*Fusarium oxysporum*<br>*Neocosmospora solani*<br>*Fusarium verticillioides*<br>*Geotrichum candidum*<br>***Linnemannia elongata***<br>*Mortierella alpina*<br>*Mucor hiemalis*<br>*Penicillium chrysogenum*<br>*Penicillium goetzii*

			Penicillium
**P5**	Narrow passage	***Cephalotrichum microsporum***<br>*Mucor plumbeus*<br>*Mucor racemosus* f. *racemosus*	*Alternaria citrimacularis*<br>*Cladosporium cladosporioides*<br>*Clonostachys rosea*<br>*Epicoccum nigrum*<br>*Fusarium oxysporum*<br>***Neocosmospora solani***<br>*Fusarium verticillioides*<br>*Penicillium brevicompactum*<br>*Penicillium chrysogenum*<br>*Penicillium goetzii*<br>*Penicillium laevigatum*<br>*Penicillium sumatrense*<br>*Penicillium*
**P6**	*Sala del Jardin*	*Cephalotrichum microsporum*<br>*Mortierella* sp.<br>*Mucor racemosus f. racemosus*	*Cephalotrichum nanum*<br>*Cladosporium cladosporioides*<br>*Fusarium oxysporum*<br>*Fusarium brachygibbosum*<br>*Neocosmospora solani*<br>*Linnemannia elongata*<br>*Penicillium chrysogenum*<br>*Penicillium goetzii*<br>*Penicillium*<br>*Trichoderma harzianum*

Table S4. Fungi identified by NGS from the sediments of Castañar Cave in 2020 and 2021. Red – relative abundances <1%.

							2020						2021					
**OTU**	**Phylum**	**Class**	**Order**	**Family**	**Genus**	**Species**	**P1**	**P2**	**P3**	**P4**	**P5**	**P6**	**P1**	**P2**	**P3**	**P4**	**P5**	**P6**
OTU1	*Aphelidiomycota*	*Aphelidiomycetes*	*GS16*	unidentified	unidentified	unidentified	0.0	0.0	0.0	0.0	0.0	0.0	0.0	0.5	1.8	0.0	0.0	0.0
OTU2	*Ascomycota*	*Archaeorhizomycetes*	*Archaeorhizomycetales*	*Archaeorhizomycetaceae*	*Archaeorhizomyces*	unidentified	0.0	0.0	0.0	0.0	0.0	0.0	9.0	0.0	0.0	0.0	0.0	0.0
OTU3	*Ascomycota*	*Dothideomycetes*	*Capnodiales*	*Cladosporiaceae*	*Cladosporium*	*Cladosporium cladosporioides*	0.0	0.0	0.0	0.0	0.0	0.0	0.0	0.2	0.0	0.0	11.2	0.0
OTU4	*Ascomycota*	*Dothideomycetes*	*Capnodiales*	*Cladosporiaceae*	*Cladosporium*	*Cladosporium sphaerospermum*	8.4	0.0	0.0	0.0	0.0	0.0	0.0	0.0	0.0	0.0	0.1	0.0
OTU5	*Ascomycota*	*Dothideomycetes*	*Capnodiales*	*Mycosphaerellaceae*	*Zymoseptoria*	unassigned	0.0	0.0	0.0	0.0	0.0	0.0	0.0	0.0	0.0	0.0	0.0	0.0
OTU6	*Ascomycota*	*Dothideomycetes*	*Dothideales*	*Aureobasidiaceae*	*Aureobasidium*	*Aureobasidium pullulans*	4.7	0.0	0.0	0.0	0.0	0.0	0.0	0.0	0.0	0.0	0.0	0.0
OTU7	*Ascomycota*	*Dothideomycetes*	*Dothideales*	*Aureobasidiaceae*	*Kabatiella*	unidentified	0.0	0.0	0.0	0.7	0.0	0.0	0.0	0.0	0.5	0.0	0.0	0.0
OTU8	*Ascomycota*	*Dothideomycetes*	*Dothideales*	*Dothioraceae*	*Hormonema*	*Hormonema viticola*	0.0	0.0	0.0	0.0	0.0	0.0	0.0	0.0	0.0	0.0	0.1	0.0
OTU9	*Ascomycota*	*Dothideomycetes*	*Dothideomycetes ord Incertae sedis*	*Eremomycetaceae*	*Arthrographis*	unassigned	0.0	0.0	0.0	0.0	0.0	0.0	0.0	0.2	0.0	0.0	0.0	0.0
OTU10	*Ascomycota*	*Dothideomycetes*	*Dothideomycetes ord Incertae sedis*	*Eremomycetaceae*	*Arthrographis*	unidentified	0.0	0.0	0.0	0.0	0.0	0.0	0.0	0.1	0.0	0.0	0.0	0.0
OTU11	*Ascomycota*	*Dothideomycetes*	*Pleosporales*	*Cucurbitariaceae*	*Pyrenochaeta*	unidentified	0.0	0.0	0.0	0.0	0.0	0.0	0.0	0.0	0.0	0.0	0.0	0.0
OTU12	*Ascomycota*	*Dothideomycetes*	*Pleosporales*	*Didymellaceae*	*Epicoccum*	*Epicoccum nigrum*	0.0	0.0	0.0	0.0	0.0	0.0	0.0	0.0	0.0	0.0	0.2	0.0
OTU13	*Ascomycota*	*Dothideomycetes*	*Pleosporales*	*Didymellaceae*	unassigned	unassigned	0.0	0.0	0.0	0.0	0.0	0.0	0.0	0.0	0.0	0.0	0.4	0.0
OTU14	*Ascomycota*	*Dothideomycetes*	*Pleosporales*	*Didymosphaeriaceae*	*Paraphaeosphaeria*	*Paraphaeosphaeria angularis*	0.0	0.0	0.0	0.0	0.0	0.0	0.0	0.0	0.0	0.0	0.0	0.0
OTU15	*Ascomycota*	*Dothideomycetes*	*Pleosporales*	*Didymosphaeriaceae*	*Pseudopithomyces*	*Pseudopithomyces rosae*	0.0	0.0	0.0	0.0	0.0	0.0	0.0	0.0	0.0	0.0	0.7	0.0
OTU16	*Ascomycota*	*Dothideomycetes*	*Pleosporales*	*Didymosphaeriaceae*	unassigned	unassigned	0.0	0.0	0.0	0.0	0.0	0.0	0.0	0.1	0.0	0.0	0.0	0.0
OTU17	*Ascomycota*	*Dothideomycetes*	*Pleosporales*	*Massarinaceae*	*Massarina*	unidentified	0.0	0.0	0.0	0.0	0.0	0.0	0.0	0.0	0.0	0.0	0.0	0.0
OTU18	*Ascomycota*	*Dothideomycetes*	*Pleosporales*	*Morosphaeriaceae*	*Acrocalymma*	unidentified	0.0	0.0	0.0	0.0	0.0	0.0	0.4	0.0	0.0	0.0	0.0	0.0
OTU19	*Ascomycota*	*Dothideomycetes*	*Pleosporales*	*Phaeosphaeriaceae*	*Paraphoma*	unidentified	0.0	0.0	0.0	0.0	0.0	0.0	0.1	0.0	0.0	0.0	0.0	0.0
OTU20	*Ascomycota*	*Dothideomycetes*	*Pleosporales*	*Pleosporaceae*	*Alternaria*	*Alternaria infectoria*	0.0	0.0	0.0	0.0	0.0	0.0	0.0	0.0	0.0	0.0	0.2	0.0
OTU21	*Ascomycota*	*Dothideomycetes*	*Pleosporales*	*Pleosporaceae*	*Alternaria*	unassigned	0.0	0.0	0.0	0.0	0.0	0.0	0.0	0.1	0.0	0.0	3.5	0.0
OTU22	*Ascomycota*	*Dothideomycetes*	*Pleosporales*	*Pleosporaceae*	*Alternaria*	unidentified	0.0	0.0	0.0	0.2	0.0	0.0	0.0	0.0	0.0	0.0	0.0	0.0
OTU23	*Ascomycota*	*Dothideomycetes*	*Pleosporales*	*Pleosporaceae*	*Curvularia*	*Curvularia inaequalis*	0.0	0.0	0.0	0.0	0.0	0.0	0.0	0.0	0.0	0.0	0.0	0.0
OTU24	*Ascomycota*	*Dothideomycetes*	*Pleosporales*	*Pleosporaceae*	*Ulocladium*	*Ulocladium chartarum*	0.0	0.0	0.0	0.0	0.0	0.0	0.0	1.5	0.0	0.1	0.8	0.9
OTU25	*Ascomycota*	*Dothideomycetes*	*Pleosporales*	*Sporormiaceae*	*Preussia*	*Preussia flanaganii*	0.0	0.0	0.0	0.0	0.0	0.0	0.0	0.0	0.5	0.0	0.0	0.0
OTU26	*Ascomycota*	*Dothideomycetes*	*Pleosporales*	*Sporormiaceae*	*Preussia*	unidentified	0.0	18.6	0.0	1.1	0.0	0.0	0.0	0.0	0.0	0.0	0.0	0.0
OTU27	*Ascomycota*	*Dothideomycetes*	*Pleosporales*	*Teichosporaceae*	unidentified	unidentified	0.0	0.0	0.0	0.0	0.0	0.0	0.0	0.0	1.4	0.0	0.0	0.0
OTU28	*Ascomycota*	*Dothideomycetes*	*Pleosporales*	unassigned	unassigned	unassigned	0.0	0.0	0.0	0.0	0.0	0.0	0.0	0.0	0.0	0.0	0.0	0.0
OTU29	*Ascomycota*	*Dothideomycetes*	*Pleosporales*	unidentified	unidentified	unidentified	0.0	0.0	0.0	0.0	0.0	0.0	0.7	0.0	0.5	0.0	0.0	0.0
OTU30	*Ascomycota*	*Dothideomycetes*	unassigned	unassigned	unassigned	unassigned	0.0	0.0	0.0	0.0	0.0	0.0	0.0	0.0	0.0	0.0	0.0	0.0

OTU31	*Ascomycota*	*Dothideomycetes*	*Venturiales*	*Sympoventuriaceae*	*Ochroconis*	*Ochroconis longiphorum*	0.0	0.0	0.0	0.0	0.0	0.0	0.0	0.0	0.0	0.0	0.0	0.0
OTU32	*Ascomycota*	*Dothideomycetes*	*Venturiales*	*Sympoventuriaceae*	*Ochroconis*	*Ochroconis tshawytschae*	0.0	0.0	0.0	0.0	0.0	0.0	0.0	0.0	0.0	0.0	0.0	0.0
OTU33	*Ascomycota*	*Eurotiomycetes*	*Chaetothyriales*	*Cyphellophoraceae*	*Cyphellophora*	*Cyphellophora olivacea*	0.7	0.0	0.0	0.0	0.0	0.0	0.0	0.1	0.0	0.0	0.0	0.0
OTU34	*Ascomycota*	*Eurotiomycetes*	*Chaetothyriales*	*Cyphellophoraceae*	*Cyphellophora*	unassigned	0.0	0.0	0.0	0.0	0.0	0.0	0.0	0.0	0.0	0.0	0.0	0.0
OTU35	*Ascomycota*	*Eurotiomycetes*	*Chaetothyriales*	*Cyphellophoraceae*	*Cyphellophora*	unidentified	1.2	0.0	0.0	0.0	0.0	0.0	0.0	0.0	0.0	0.0	0.0	0.0
OTU36	*Ascomycota*	*Eurotiomycetes*	*Chaetothyriales*	*Herpotrichiellaceae*	*Capronia*	*Capronia coronata*	0.0	0.0	0.0	0.0	0.0	0.0	0.0	0.0	0.0	0.0	0.1	0.0
OTU37	*Ascomycota*	*Eurotiomycetes*	*Chaetothyriales*	*Herpotrichiellaceae*	*Exophiala*	unidentified	0.0	0.0	0.0	0.0	0.0	0.0	0.0	0.0	0.0	0.0	0.0	0.0
OTU38	*Ascomycota*	*Eurotiomycetes*	*Chaetothyriales*	*Herpotrichiellaceae*	*Minimelanolocus*	*Minimelanolocus aquaticus*	0.0	0.0	0.0	0.0	0.0	0.0	0.0	0.0	0.0	0.0	0.0	0.0
OTU39	*Ascomycota*	*Eurotiomycetes*	*Chaetothyriales*	*Herpotrichiellaceae*	*Minimelanolocus*	unassigned	0.0	0.0	0.0	0.0	0.0	0.0	0.0	0.0	0.0	0.0	0.0	0.0
OTU40	*Ascomycota*	*Eurotiomycetes*	*Chaetothyriales*	*Herpotrichiellaceae*	*Rhinocladiella*	unidentified	0.0	0.0	0.0	0.0	0.0	0.0	0.0	0.0	0.0	0.0	0.0	0.0
OTU41	*Ascomycota*	*Eurotiomycetes*	*Chaetothyriales*	*Herpotrichiellaceae*	unassigned	unassigned	0.0	0.0	0.0	0.0	0.0	0.0	0.1	0.0	0.0	0.0	0.1	0.0
OTU42	*Ascomycota*	*Eurotiomycetes*	*Chaetothyriales*	*Herpotrichiellaceae*	*Veronaea*	unassigned	0.0	0.0	0.0	0.0	0.0	0.0	0.0	0.0	0.0	0.0	0.1	0.0
OTU43	*Ascomycota*	*Eurotiomycetes*	*Chaetothyriales*	unassigned	unassigned	unassigned	0.0	0.0	0.0	0.0	0.0	0.0	0.2	0.1	0.0	0.0	0.0	0.0
OTU44	*Ascomycota*	*Eurotiomycetes*	*Chaetothyriales*	unidentified	unidentified	unidentified	0.0	0.0	0.0	0.0	0.0	0.0	0.0	0.3	0.0	0.0	0.0	0.0
OTU45	*Ascomycota*	*Eurotiomycetes*	*Eurotiales*	*Aspergillaceae*	*Aspergillus*	*Aspergillus flavus*	0.0	0.0	0.0	0.0	0.0	0.0	0.0	0.0	0.0	0.4	0.7	0.0
OTU46	*Ascomycota*	*Eurotiomycetes*	*Eurotiales*	*Aspergillaceae*	*Aspergillus*	*Aspergillus protuberus*	6.5	0.0	0.0	0.0	0.0	0.0	0.0	0.0	0.0	0.0	0.0	0.0
OTU47	*Ascomycota*	*Eurotiomycetes*	*Eurotiales*	*Aspergillaceae*	*Aspergillus*	*Aspergillus puniceus*	0.0	0.0	0.0	0.0	0.0	0.0	0.0	0.0	0.0	0.0	0.0	0.0
OTU48	*Ascomycota*	*Eurotiomycetes*	*Eurotiales*	*Aspergillaceae*	*Aspergillus*	unassigned	0.0	0.0	0.0	0.0	0.0	0.0	0.0	0.1	0.0	0.0	0.0	0.0
OTU49	*Ascomycota*	*Eurotiomycetes*	*Eurotiales*	*Aspergillaceae*	*Aspergillus*	unidentified	9.7	0.0	0.0	0.0	0.0	0.0	0.0	0.0	0.0	0.0	0.0	0.0
OTU50	*Ascomycota*	*Eurotiomycetes*	*Eurotiales*	*Aspergillaceae*	*Penicillium*	*Penicillium brasilianum*	0.0	0.0	0.0	0.0	0.0	0.0	0.0	0.0	0.0	0.0	0.0	0.0
OTU51	*Ascomycota*	*Eurotiomycetes*	*Eurotiales*	*Aspergillaceae*	*Penicillium*	*Penicillium brevicompactum*	0.0	0.0	0.0	0.0	0.0	0.0	0.0	0.0	0.0	0.0	0.0	0.0
OTU52	*Ascomycota*	*Eurotiomycetes*	*Eurotiales*	*Aspergillaceae*	*Penicillium*	*Penicillium camemberti*	0.0	0.0	0.0	0.0	0.0	0.0	0.0	0.0	0.0	0.0	0.0	0.0
OTU53	*Ascomycota*	*Eurotiomycetes*	*Eurotiales*	*Aspergillaceae*	*Penicillium*	*Penicillium citrinum*	0.0	0.0	0.0	0.0	3.6	0.0	0.0	0.0	0.0	0.0	0.0	0.0
OTU54	*Ascomycota*	*Eurotiomycetes*	*Eurotiales*	*Aspergillaceae*	*Penicillium*	*Penicillium expansum*	0.0	0.0	0.0	0.0	0.0	0.0	0.0	0.2	0.4	0.0	0.0	0.0
OTU55	*Ascomycota*	*Eurotiomycetes*	*Eurotiales*	*Aspergillaceae*	*Penicillium*	unassigned	0.0	0.0	0.0	0.0	0.0	0.0	0.0	0.1	0.0	0.3	0.3	0.0
OTU56	*Ascomycota*	*Eurotiomycetes*	*Eurotiales*	*Aspergillaceae*	*Penicillium*	unidentified	0.0	1.6	0.0	0.0	0.0	0.0	0.0	0.0	0.0	0.0	0.0	0.0
OTU57	*Ascomycota*	*Eurotiomycetes*	*Eurotiales*	*Aspergillaceae*	unidentified	unidentified	0.8	0.0	0.0	0.0	0.0	0.0	0.0	0.0	0.0	0.0	0.0	0.0
OTU58	*Ascomycota*	*Eurotiomycetes*	*Eurotiales*	*Trichocomaceae*	*Talaromyces*	*Talaromyces acaricola*	9.4	0.0	0.0	0.0	0.0	0.0	0.0	0.0	0.0	0.0	0.0	0.0
OTU59	*Ascomycota*	*Eurotiomycetes*	*Eurotiales*	*Trichocomaceae*	*Talaromyces*	*Talaromyces diversus*	0.0	0.0	0.0	0.0	0.0	0.0	0.0	0.0	0.0	0.0	0.0	0.0
OTU60	*Ascomycota*	*Eurotiomycetes*	*Eurotiales*	*Trichocomaceae*	*Talaromyces*	*Talaromyces minioluteus*	0.0	0.0	0.0	0.0	0.0	0.0	0.0	0.0	0.0	0.0	0.0	0.0
OTU61	*Ascomycota*	*Eurotiomycetes*	*Eurotiales*	*Trichocomaceae*	*Talaromyces*	*Talaromyces rugulosus*	0.0	0.0	0.0	0.0	0.0	0.0	0.0	1.2	0.0	0.0	0.0	0.0
OTU62	*Ascomycota*	*Eurotiomycetes*	*Eurotiales*	*Trichocomaceae*	*Talaromyces*	*Talaromyces wortmannii*	5.9	0.0	0.0	0.0	0.0	0.0	0.0	0.0	0.0	0.0	0.0	0.0
OTU63	*Ascomycota*	*Eurotiomycetes*	*Eurotiales*	*Trichocomaceae*	*Talaromyces*	unidentified	1.2	1.3	0.0	0.0	0.0	0.0	0.0	0.0	0.0	0.0	0.0	0.0

OTU64	*Ascomycota*	*Eurotiomycetes*	*Eurotiales*	*Trichocomaceae*	unidentified	unidentified	0.0	0.0	0.0	0.0	0.0	0.0	0.0	0.0	0.2	0.0	0.0	0.0
OTU65	*Ascomycota*	*Eurotiomycetes*	*Onygenales*	*Ajellomycetaceae*	*Emergomyces*	*Emergomyces europaeus*	0.0	0.0	0.0	0.0	0.0	0.0	0.0	0.0	0.0	0.0	0.0	0.0
OTU66	*Ascomycota*	*Eurotiomycetes*	*Onygenales*	*Arthrodermataceae*	*Arthroderma*	*Arthroderma multifidum*	0.0	0.0	0.0	0.0	0.0	0.0	0.0	0.0	0.1	0.0	0.0	0.0
OTU67	*Ascomycota*	*Eurotiomycetes*	*Onygenales*	*Arthrodermataceae*	*Arthroderma*	*Arthroderma redellii*	0.0	0.0	0.0	0.0	0.0	0.0	0.0	0.6	0.0	0.0	0.0	0.0
OTU68	*Ascomycota*	*Eurotiomycetes*	*Onygenales*	*Arthrodermataceae*	*Trichophyton*	*Trichophyton ajelloi*	0.0	0.0	0.0	0.0	29.4	0.0	0.0	0.0	0.0	0.0	0.0	0.0
OTU69	*Ascomycota*	*Eurotiomycetes*	*Onygenales*	*Gymnoascaceae*	*Gymnoascus*	*Gymnoascus dugwayensis*	0.0	0.0	0.0	0.0	0.0	0.0	0.0	0.0	0.0	0.0	0.0	0.0
OTU70	*Ascomycota*	*Eurotiomycetes*	*Onygenales*	*Gymnoascaceae*	*Gymnoascus*	*Gymnoascus reessii*	0.0	0.0	0.0	0.0	0.0	0.0	0.0	0.1	0.5	0.0	0.0	0.0
OTU71	*Ascomycota*	*Eurotiomycetes*	*Onygenales*	*Gymnoascaceae*	*Gymnoascus*	unidentified	0.0	0.0	0.0	0.0	0.0	0.0	0.0	0.0	0.0	0.0	0.0	0.0
OTU72	*Ascomycota*	*Eurotiomycetes*	*Onygenales*	*Gymnoascaceae*	*Leucothecium*	*Leucothecium emdenii*	0.0	0.0	0.0	0.0	0.0	0.0	0.0	0.0	1.1	0.0	0.0	0.0
OTU73	*Ascomycota*	*Eurotiomycetes*	*Onygenales*	*Gymnoascaceae*	unidentified	unidentified	0.0	0.0	0.0	0.0	0.0	0.0	0.0	0.0	0.1	0.0	0.0	0.0
OTU74	*Ascomycota*	*Eurotiomycetes*	*Onygenales*	*Onygenaceae*	*Auxarthron*	*Auxarthron ostraviense*	0.0	0.0	0.0	0.0	0.0	0.0	0.0	0.6	0.0	0.1	0.0	0.0
OTU75	*Ascomycota*	*Eurotiomycetes*	*Onygenales*	*Onygenaceae*	*Auxarthronopsis*	unidentified	0.0	0.0	0.0	0.0	0.0	0.0	0.0	0.1	0.0	0.0	0.0	0.0
OTU76	*Ascomycota*	*Eurotiomycetes*	*Onygenales*	*Onygenaceae*	*Mallochia*	*Mallochia reticulata*	0.0	0.0	0.0	0.0	0.0	0.0	0.0	1.1	0.0	0.0	0.0	0.0
OTU77	*Ascomycota*	*Eurotiomycetes*	*Onygenales*	*Onygenaceae*	*Myriodontium*	*Myriodontium keratinophilum*	0.0	0.0	0.0	0.0	0.0	0.0	0.0	0.1	0.0	0.0	0.0	0.0
OTU78	*Ascomycota*	*Eurotiomycetes*	*Onygenales*	*Onygenaceae*	*Pectinotrichum*	unidentified	0.0	0.0	0.0	0.0	0.0	0.0	0.0	0.0	0.0	0.0	0.0	0.0
OTU79	*Ascomycota*	*Eurotiomycetes*	*Onygenales*	*Onygenaceae*	unidentified	unidentified	0.0	0.0	0.0	0.0	0.0	0.0	0.0	0.2	0.3	0.0	0.0	0.0
OTU80	*Ascomycota*	*Eurotiomycetes*	*Onygenales*	*Onygenales fam Incertae sedis*	*Arthropsis*	*Arthropsis hispanica*	0.0	0.0	0.0	0.0	0.0	0.0	0.0	0.0	0.0	0.2	0.1	0.0
OTU81	*Ascomycota*	*Eurotiomycetes*	*Onygenales*	*Onygenales fam Incertae sedis*	*Chrysosporium*	*Chrysosporium pseudomerdarium*	0.0	7.4	0.0	0.0	0.0	0.0	0.0	0.1	0.8	0.0	0.0	0.0
OTU82	*Ascomycota*	*Eurotiomycetes*	*Onygenales*	*Onygenales fam Incertae sedis*	*Chrysosporium*	*Chrysosporium undulatum*	0.0	0.0	0.0	0.0	0.0	0.0	0.0	0.2	0.0	0.0	0.0	0.0
OTU83	*Ascomycota*	*Eurotiomycetes*	*Onygenales*	*Onygenales fam Incertae sedis*	*Chrysosporium*	unassigned	0.0	0.0	0.0	0.0	0.0	0.0	0.0	0.0	0.6	0.0	0.0	0.0
OTU84	*Ascomycota*	*Eurotiomycetes*	*Onygenales*	*Onygenales fam Incertae sedis*	*Malbranchea*	*Malbranchea flocciformis*	0.0	0.0	0.0	0.0	0.0	0.0	0.0	0.4	0.0	0.0	0.0	0.0
OTU85	*Ascomycota*	*Eurotiomycetes*	*Onygenales*	*Onygenales fam Incertae sedis*	*Malbranchea*	unassigned	0.0	0.0	0.0	0.0	0.0	0.0	0.0	1.5	0.0	0.0	0.0	0.0
OTU86	*Ascomycota*	*Eurotiomycetes*	*Onygenales*	unassigned	unassigned	unassigned	0.0	0.0	0.0	0.0	0.0	0.0	0.0	0.8	0.0	0.0	0.0	0.0
OTU87	*Ascomycota*	*Eurotiomycetes*	*Onygenales*	unidentified	unidentified	unidentified	0.0	0.0	0.0	0.0	0.0	0.0	11.0	0.0	0.0	0.0	0.0	0.0
OTU88	*Ascomycota*	*Eurotiomycetes*	unassigned	unassigned	unassigned	unassigned	0.0	0.0	0.0	0.0	0.0	0.0	0.0	0.1	0.0	0.0	0.0	0.0
OTU89	*Ascomycota*	*Eurotiomycetes*	unidentified	unidentified	unidentified	unidentified	0.0	0.0	0.0	0.0	0.0	0.0	3.7	0.0	0.0	0.0	0.0	0.0
OTU90	*Ascomycota*	*Geoglossomycetes*	*Geoglossales*	*Geoglossaceae*	*Geoglossum*	unidentified	0.0	0.0	0.0	0.0	0.0	0.0	1.3	0.0	0.0	0.0	0.0	0.0
OTU91	*Ascomycota*	*Geoglossomycetes*	*Geoglossales*	*Geoglossaceae*	*Trichoglossum*	*Trichoglossum hirsutum*	0.0	0.0	0.0	0.0	0.0	0.0	0.0	0.0	0.0	0.0	0.0	0.0
OTU92	*Ascomycota*	*Geoglossomycetes*	*Geoglossales*	*unidentified*	unidentified	unidentified	0.0	0.0	0.0	0.0	0.0	0.0	0.1	0.0	0.0	0.0	0.0	0.0
OTU93	*Ascomycota*	*Leotiomycetes*	*Erysiphales*	*Erysiphaceae*	unidentified	unidentified	0.0	0.0	0.0	0.5	0.0	0.0	0.0	0.0	0.0	0.0	0.0	0.0
OTU94	*Ascomycota*	*Leotiomycetes*	*Helotiales*	*Helotiaceae*	*Scytalidium*	*unassigned*	0.0	0.0	0.0	0.0	0.0	0.0	0.0	0.0	0.0	0.0	0.0	0.0
OTU95	*Ascomycota*	*Leotiomycetes*	*Helotiales*	*Helotiaceae*	*Scytalidium*	*unidentified*	0.0	0.0	0.0	0.0	0.0	0.0	0.0	0.2	0.0	0.0	0.0	0.0
OTU96	*Ascomycota*	*Leotiomycetes*	*Helotiales*	*Helotiaceae*	*Tetracladium*	*Tetracladium apiense*	0.0	0.0	0.0	0.0	0.0	0.0	0.0	0.0	0.0	0.0	0.0	0.0

OTU97	*Ascomycota*	*Leotiomycetes*	*Helotiales*	*Helotiaceae*	*Tetracladium*	*unidentified*	0.0	0.0	0.0	0.0	0.0	0.0	0.0	2.4	0.0	0.0	0.0	0.0
OTU98	*Ascomycota*	*Leotiomycetes*	*Helotiales*	*Helotiales fam Incertae sedis*	*Kendrickiella*	*Kendrickiella phycomyces*	0.0	0.0	0.0	0.0	0.0	0.0	0.0	0.0	0.0	0.0	0.0	0.0
OTU99	*Ascomycota*	*Leotiomycetes*	*Helotiales*	*Helotiales fam Incertae sedis*	*Rhexocercosporidium*	*Rhexocercosporidium panacis*	4.2	0.0	0.0	0.0	0.0	0.0	0.0	0.1	0.0	0.8	0.0	0.0
OTU100	*Ascomycota*	*Leotiomycetes*	*Helotiales*	*Hyaloscyphaceae*	*Lachnum*	*unidentified*	0.0	0.0	0.0	0.0	0.0	0.0	0.0	0.1	0.0	0.0	0.0	0.0
OTU101	*Ascomycota*	*Leotiomycetes*	*Helotiales*	*Myxotrichaceae*	*Oidiodendron*	*Oidiodendron echinulatum*	0.0	0.0	0.0	0.0	0.0	0.0	0.0	0.1	0.0	0.0	0.0	0.0
OTU102	*Ascomycota*	*Leotiomycetes*	*Helotiales*	*Myxotrichaceae*	*Oidiodendron*	*unassigned*	0.0	0.0	0.0	0.0	0.0	0.0	0.0	1.7	4.5	0.0	0.0	0.0
OTU103	*Ascomycota*	*Leotiomycetes*	*Helotiales*	*Myxotrichaceae*	*Oidiodendron*	*unidentified*	0.0	0.0	0.0	0.0	0.0	0.0	0.0	1.9	0.0	0.0	0.0	0.0
OTU104	*Ascomycota*	*Leotiomycetes*	*Helotiales*	*Pezizellaceae*	*Porodiplodia*	*Porodiplodia vitis*	7.4	0.0	0.0	0.0	0.0	0.0	0.0	0.0	0.0	0.0	0.0	0.0
OTU105	*Ascomycota*	*Leotiomycetes*	*Helotiales*	*unassigned*	unassigned	unassigned	0.0	0.0	0.0	0.0	0.0	0.0	0.1	0.6	0.0	0.0	0.0	0.0
OTU106	*Ascomycota*	*Leotiomycetes*	*Phacidiales*	*Phacidiaceae*	*Phacidium*	*Phacidium lacerum*	0.0	0.0	0.0	0.0	0.0	0.0	0.0	0.0	0.0	0.0	0.0	0.0
OTU107	*Ascomycota*	*Leotiomycetes*	*Rhytismatales*	*Rhytismataceae*	unassigned	unassigned	0.0	0.0	0.0	0.0	0.0	0.0	0.0	0.0	0.0	0.0	0.0	0.0
OTU108	*Ascomycota*	*Leotiomycetes*	*Thelebolales*	*Pseudeurotiaceae*	*Pleuroascus*	*Pleuroascus nicholsonii*	0.0	0.0	0.0	0.0	0.0	0.0	0.0	0.7	0.0	0.0	0.0	0.0
OTU109	*Ascomycota*	*Leotiomycetes*	*Thelebolales*	*Pseudeurotiaceae*	*Pseudeurotium*	*Pseudeurotium desertorum*	0.0	0.0	0.0	0.0	0.0	0.0	0.0	0.5	0.0	0.0	0.0	0.0
OTU110	*Ascomycota*	*Leotiomycetes*	*Thelebolales*	*Pseudeurotiaceae*	*Pseudeurotium*	unassigned	0.0	0.0	0.0	0.0	0.0	0.0	0.0	0.3	1.8	0.0	0.0	0.0
OTU111	*Ascomycota*	*Leotiomycetes*	*Thelebolales*	*Pseudeurotiaceae*	*Pseudogymnoascus*	unassigned	0.0	0.0	0.0	0.0	0.0	0.0	0.0	0.0	0.0	0.0	0.0	0.0
OTU112	*Ascomycota*	*Leotiomycetes*	*Thelebolales*	*Pseudeurotiaceae*	*Pseudogymnoascus*	*Pseudogymnoascus pannorum*	0.0	3.4	0.0	1.8	0.0	0.0	9.9	0.9	0.0	1.1	1.4	3.9
OTU113	*Ascomycota*	*Leotiomycetes*	*Thelebolales*	*Pseudeurotiaceae*	unassigned	unassigned	0.0	0.0	0.0	0.0	0.0	0.0	3.8	0.2	0.0	0.0	0.0	0.0
OTU114	*Ascomycota*	*Leotiomycetes*	unassigned	unassigned	unassigned	unassigned	0.0	0.0	0.0	0.0	0.0	0.0	0.0	0.0	0.0	0.0	0.0	0.0
OTU115	*Ascomycota*	*Orbiliomycetes*	*Orbiliales*	*Orbiliaceae*	*Dactylaria*	*Dactylaria mitrata*	0.0	0.0	0.0	0.0	0.0	0.0	0.0	0.0	0.0	0.0	0.0	0.0
OTU116	*Ascomycota*	*Pezizomycetes*	*Pezizales*	*Helvellaceae*	*Balsamia*	*Balsamia platyspora*	0.0	0.0	0.0	0.0	0.0	0.0	4.4	0.0	0.0	0.0	0.0	0.0
OTU117	*Ascomycota*	*Pezizomycetes*	*Pezizales*	*Pezizaceae*	*Hydnobolites*	*Hydnobolites cerebriformis*	0.0	0.0	0.0	0.0	0.0	0.0	0.0	0.0	0.0	0.0	0.0	0.0
OTU118	*Ascomycota*	*Pezizomycetes*	*Pezizales*	*Pyronemataceae*	*Pseudaleuria*	unidentified	0.0	0.0	0.0	0.0	0.0	0.0	0.0	0.1	0.0	0.0	0.0	0.0
OTU119	*Ascomycota*	*Pezizomycetes*	*Pezizales*	*Pyronemataceae*	unidentified	unidentified	0.0	0.0	0.0	0.0	0.0	0.0	0.1	0.3	0.0	0.0	0.0	0.0
OTU120	*Ascomycota*	*Pezizomycetes*	*Pezizales*	*Tuberaceae*	*Tuber*	*Tuber fulgens*	0.0	0.0	0.0	0.0	0.0	0.0	0.8	0.0	0.0	0.0	0.0	0.0
OTU121	*Ascomycota*	*Pezizomycetes*	*Pezizales*	*Tuberaceae*	*Tuber*	*Tuber scruposum*	0.0	0.0	0.0	0.0	0.0	0.0	0.6	0.0	0.0	0.0	0.0	0.0
OTU122	*Ascomycota*	*Saccharomycetes*	*Saccharomycetales*	*Debaryomycetaceae*	*Meyerozyma*	*Meyerozyma guilliermondii*	0.0	0.0	0.0	3.5	0.0	0.0	0.0	0.0	0.0	0.0	0.0	0.0
OTU123	*Ascomycota*	*Saccharomycetes*	*Saccharomycetales*	*Phaffomycetaceae*	*Barnettozyma*	unidentified	0.0	0.0	0.0	0.0	0.0	0.0	0.0	0.0	0.0	0.0	0.0	0.0
OTU124	*Ascomycota*	*Saccharomycetes*	*Saccharomycetales*	*Saccharomycetales fam Incertae sedis*	*Candida*	*Candida friedrichii*	0.0	0.0	0.0	0.0	0.0	0.0	0.0	0.1	0.0	0.0	0.0	0.0
OTU125	*Ascomycota*	*Saccharomycetes*	*Saccharomycetales*	*Saccharomycetales fam Incertae sedis*	*Candida*	*Candida parapsilosis*	0.0	9.5	0.0	0.0	0.0	50.0	0.0	0.0	0.0	0.0	0.0	0.0
OTU126	*Ascomycota*	*Sordariomycetes*	*Branch06*	unidentified	unidentified	unidentified	0.0	0.0	0.0	0.0	0.0	0.0	0.0	0.0	0.0	0.0	0.0	0.0
OTU127	*Ascomycota*	*Sordariomycetes*	*Diaporthales*	*Diaporthaceae*	*Diaporthe*	unidentified	0.0	0.0	0.0	0.0	19.0	0.0	0.0	0.0	0.0	0.0	0.0	0.0
OTU128	*Ascomycota*	*Sordariomycetes*	*Glomerellales*	*Plectosphaerellaceae*	*Chordomyces*	*Chordomyces antarcticus*	0.0	0.0	0.0	0.0	0.0	0.0	0.0	0.0	0.0	0.0	0.1	0.0
OTU129	*Ascomycota*	*Sordariomycetes*	*Glomerellales*	*Plectosphaerellaceae*	*Musicillium*	*Musicillium theobromae*	0.0	0.0	0.0	0.0	0.0	0.0	0.0	0.0	0.0	0.0	0.0	0.0

OTU130	*Ascomycota*	*Sordariomycetes*	*Glomerellales*	*Plectosphaerellaceae*	*Plectosphaerella*	*Plectosphaerella cucumerina*	0.0	0.0	0.0	0.0	0.0	0.0	0.0	1.4	0.0	0.0	0.0	0.0
OTU131	*Ascomycota*	*Sordariomycetes*	*Glomerellales*	*Plectosphaerellaceae*	*Plectosphaerella*	unidentified	0.0	0.0	0.0	0.0	0.0	0.0	0.0	0.0	0.0	0.1	0.0	0.0
OTU132	*Ascomycota*	*Sordariomycetes*	*Glomerellales*	*Plectosphaerellaceae*	unidentified	unidentified	0.0	0.0	0.0	0.4	0.0	0.0	0.0	0.0	0.0	0.0	0.0	0.0
OTU133	*Ascomycota*	*Sordariomycetes*	*Glomerellales*	*Plectosphaerellaceae*	*Verticillium*	*Verticillium leptobactrum*	0.0	0.0	0.0	0.0	0.0	0.0	0.0	0.0	0.0	0.0	0.0	0.0
OTU134	*Ascomycota*	*Sordariomycetes*	*Hypocreales*	*Bionectriaceae*	*Clonostachys*	*Clonostachys rosea*	0.3	0.0	0.0	0.0	0.0	0.0	0.0	0.0	0.5	0.0	0.0	0.0
OTU135	*Ascomycota*	*Sordariomycetes*	*Hypocreales*	*Bionectriaceae*	*Nectriopsis*	unassigned	0.0	0.0	0.0	0.0	0.0	0.0	0.0	0.0	0.0	0.0	0.0	0.0
OTU136	*Ascomycota*	*Sordariomycetes*	*Hypocreales*	*Bionectriaceae*	unidentified	unidentified	0.0	0.0	0.0	0.0	0.0	0.0	0.0	0.0	1.1	0.0	0.0	0.0
OTU137	*Ascomycota*	*Sordariomycetes*	*Hypocreales*	*Clavicipitaceae*	*Metacordyceps*	*Metacordyceps chlamydosporia*	0.7	0.0	0.0	0.0	0.0	0.0	0.0	2.2	0.0	2.8	0.7	3.2
OTU138	*Ascomycota*	*Sordariomycetes*	*Hypocreales*	*Clavicipitaceae*	*Metapochonia*	unidentified	0.0	0.0	0.0	0.0	0.0	0.0	0.0	0.0	0.0	0.0	0.0	0.0
OTU139	*Ascomycota*	*Sordariomycetes*	*Hypocreales*	*Clavicipitaceae*	*Metarhizium*	*Metarhizium anisopliae*	0.0	0.0	0.0	0.0	0.0	0.0	0.0	0.0	0.0	0.1	0.0	0.0
OTU140	*Ascomycota*	*Sordariomycetes*	*Hypocreales*	*Clavicipitaceae*	*Metarhizium*	*Metarhizium flavoviride*	0.0	0.0	0.0	0.0	0.0	0.0	0.0	0.1	0.0	0.0	0.0	0.0
OTU141	*Ascomycota*	*Sordariomycetes*	*Hypocreales*	*Clavicipitaceae*	*Metarhizium*	*Metarhizium marquandii*	0.0	0.0	0.0	0.2	0.0	0.0	0.0	0.0	0.0	0.0	0.0	0.0
OTU142	*Ascomycota*	*Sordariomycetes*	*Hypocreales*	*Clavicipitaceae*	*Metarhizium*	*Metarhizium pemphigi*	0.0	0.0	0.0	0.0	0.0	0.0	0.0	0.0	0.0	0.0	0.0	0.0
OTU143	*Ascomycota*	*Sordariomycetes*	*Hypocreales*	*Clavicipitaceae*	*Paecilomyces*	*Paecilomyces penicillatus*	0.0	0.0	0.0	0.0	0.0	0.0	0.0	0.0	0.0	0.0	0.0	0.0
OTU144	*Ascomycota*	*Sordariomycetes*	*Hypocreales*	*Cordycipitaceae*	*Beauveria*	*Beauveria felina*	0.0	0.0	0.0	0.0	0.0	0.0	0.0	0.8	0.0	0.0	0.0	0.0
OTU145	*Ascomycota*	*Sordariomycetes*	*Hypocreales*	*Cordycipitaceae*	*Beauveria*	unidentified	0.0	0.0	0.0	0.0	0.0	0.0	0.0	0.0	0.0	0.0	0.0	0.0
OTU146	*Ascomycota*	*Sordariomycetes*	*Hypocreales*	*Cordycipitaceae*	*Engyodontium*	*Engyodontium album*	1.0	0.0	0.0	0.0	0.0	0.0	0.0	0.0	0.0	0.0	0.0	0.0
OTU147	*Ascomycota*	*Sordariomycetes*	*Hypocreales*	*Cordycipitaceae*	*Lecanicillium*	*Lecanicillium antillanum*	0.0	0.0	0.0	0.0	0.0	0.0	0.0	0.2	0.0	0.0	0.0	0.0
OTU148	*Ascomycota*	*Sordariomycetes*	*Hypocreales*	*Cordycipitaceae*	*Lecanicillium*	*Lecanicillium psalliotae*	0.0	0.0	0.0	0.0	0.0	0.0	0.0	0.0	0.0	0.0	0.0	0.0
OTU149	*Ascomycota*	*Sordariomycetes*	*Hypocreales*	*Cordycipitaceae*	*Lecanicillium*	unassigned	0.0	0.0	0.0	0.0	0.0	0.0	0.0	0.0	0.0	13.3	0.0	0.0
OTU150	*Ascomycota*	*Sordariomycetes*	*Hypocreales*	*Cordycipitaceae*	*Lecanicillium*	unidentified	1.6	0.0	0.0	0.0	0.0	0.0	0.0	0.1	0.0	0.1	0.1	0.0
OTU151	*Ascomycota*	*Sordariomycetes*	*Hypocreales*	*Cordycipitaceae*	*Leptobacillium*	*Leptobacillium leptobactrum*	0.0	0.0	0.0	1.2	0.0	0.0	0.0	0.0	0.0	0.0	0.0	0.2
OTU152	*Ascomycota*	*Sordariomycetes*	*Hypocreales*	*Cordycipitaceae*	*Simplicillium*	*Simplicillium lanosoniveum*	0.0	0.0	0.0	0.0	0.0	0.0	0.0	0.0	0.0	0.0	0.0	0.0
OTU153	*Ascomycota*	*Sordariomycetes*	*Hypocreales*	*Cordycipitaceae*	*Simplicillium*	*Simplicillium minatense*	0.8	0.0	0.0	0.0	0.0	0.0	0.0	0.0	0.0	0.1	0.0	0.0
OTU154	*Ascomycota*	*Sordariomycetes*	*Hypocreales*	*Cordycipitaceae*	unassigned	unassigned	0.0	0.0	0.0	0.0	0.0	0.0	0.0	0.0	0.0	0.0	0.0	0.0
OTU155	*Ascomycota*	*Sordariomycetes*	*Hypocreales*	*Cordycipitaceae*	unidentified	unidentified	1.0	0.0	0.0	0.0	0.0	0.0	0.0	0.0	0.0	0.0	0.0	0.0
OTU156	*Ascomycota*	*Sordariomycetes*	*Hypocreales*	*Hypocreaceae*	*Monocillium*	*Monocillium griseo-ochraceum*	0.0	0.0	0.0	0.0	0.0	0.0	0.0	0.0	0.0	0.0	0.0	0.0
OTU157	*Ascomycota*	*Sordariomycetes*	*Hypocreales*	*Hypocreaceae*	*Trichoderma*	*Trichoderma viride*	0.0	0.0	0.0	0.0	0.0	0.0	0.0	0.0	0.0	0.0	0.0	0.0
OTU158	*Ascomycota*	*Sordariomycetes*	*Hypocreales*	*Hypocreales fam Incertae sedis*	*Acremonium*	*Acremonium alternatum*	0.0	0.0	0.0	0.0	0.0	0.0	0.0	0.0	0.0	0.0	0.0	0.0
OTU159	*Ascomycota*	*Sordariomycetes*	*Hypocreales*	*Hypocreales fam Incertae sedis*	*Acremonium*	*Acremonium brachypenium*	0.0	0.0	100.0	0.0	0.0	0.0	0.0	0.0	0.0	0.0	0.0	0.0
OTU160	*Ascomycota*	*Sordariomycetes*	*Hypocreales*	*Hypocreales fam Incertae sedis*	*Acremonium*	*Acremonium charticola*	0.0	0.0	0.0	0.0	0.0	0.0	0.0	0.0	0.0	0.0	0.0	0.0
OTU161	*Ascomycota*	*Sordariomycetes*	*Hypocreales*	*Hypocreales fam Incertae sedis*	*Acremonium*	*Acremonium exuviarum*	0.0	0.0	0.0	0.0	0.0	0.0	0.0	0.0	0.0	0.0	0.1	0.0
OTU162	*Ascomycota*	*Sordariomycetes*	*Hypocreales*	*Hypocreales fam Incertae sedis*	*Acremonium*	*Acremonium furcatum*	0.0	0.0	0.0	0.0	0.0	0.0	0.0	0.0	0.0	0.0	0.0	0.0

OTU163	*Ascomycota*	*Sordariomycetes*	*Hypocreales*	*Hypocreales fam Incertae sedis*	*Acremonium*	*Acremonium persicinum*	0.0	0.0	0.0	0.0	0.0	0.0	0.0	0.0	0.0	0.0	0.0	0.0
OTU164	*Ascomycota*	*Sordariomycetes*	*Hypocreales*	*Hypocreales fam Incertae sedis*	*Acremonium*	*Acremonium polychromum*	0.0	0.0	0.0	0.0	0.0	0.0	0.0	0.0	0.0	0.0	0.0	0.1
OTU165	*Ascomycota*	*Sordariomycetes*	*Hypocreales*	*Hypocreales fam Incertae sedis*	*Acremonium*	*Acremonium pteridii*	0.0	0.0	0.0	0.0	0.0	0.0	0.0	0.0	0.7	0.0	0.0	0.0
OTU166	*Ascomycota*	*Sordariomycetes*	*Hypocreales*	*Hypocreales fam Incertae sedis*	*Acremonium*	*Acremonium roseolum*	0.0	0.0	0.0	0.0	0.0	0.0	0.0	0.0	0.0	0.0	0.0	0.0
OTU167	*Ascomycota*	*Sordariomycetes*	*Hypocreales*	*Hypocreales fam Incertae sedis*	*Acremonium*	*Acremonium spinosum*	0.0	0.0	0.0	0.0	0.0	0.0	0.0	0.0	0.7	0.0	0.0	0.0
OTU168	*Ascomycota*	*Sordariomycetes*	*Hypocreales*	*Hypocreales fam Incertae sedis*	*Trichothecium*	*Trichothecium ovalisporum*	0.0	0.0	0.0	0.0	0.0	0.0	0.0	0.0	0.0	0.0	0.0	0.0
OTU169	*Ascomycota*	*Sordariomycetes*	*Hypocreales*	*Nectriaceae*	*Cosmospora*	unidentified	0.0	0.0	0.0	0.0	0.0	0.0	0.0	0.0	0.2	0.0	0.0	0.0
OTU170	*Ascomycota*	*Sordariomycetes*	*Hypocreales*	*Nectriaceae*	*Cylindrocarpon*	unidentified	0.0	0.0	0.0	0.0	0.0	0.0	0.0	0.3	0.0	0.0	0.0	0.0
OTU171	*Ascomycota*	*Sordariomycetes*	*Hypocreales*	*Nectriaceae*	*Dactylonectria*	*Dactylonectria macrodidyma*	0.0	0.0	0.0	0.0	0.0	0.0	0.1	0.1	0.0	0.0	0.0	0.0
OTU172	*Ascomycota*	*Sordariomycetes*	*Hypocreales*	*Nectriaceae*	*Fusarium*	*Fusarium neocosmosporiellum*	0.0	0.0	0.0	0.0	0.0	0.0	0.0	0.1	0.0	0.0	0.0	0.0
OTU173	*Ascomycota*	*Sordariomycetes*	*Hypocreales*	*Nectriaceae*	*Fusarium*	*Fusarium oxysporum*	0.0	4.5	0.0	0.2	0.0	0.0	0.0	0.0	0.0	0.0	3.2	10.3
OTU174	*Ascomycota*	*Sordariomycetes*	*Hypocreales*	*Nectriaceae*	*Fusarium*	*Fusarium petroliphilum*	0.0	0.0	0.0	0.0	0.0	0.0	0.0	0.3	0.0	0.0	0.0	0.0
OTU175	*Ascomycota*	*Sordariomycetes*	*Hypocreales*	*Nectriaceae*	*Fusarium*	unidentified	0.0	0.0	0.0	0.1	0.0	0.0	0.0	0.0	0.0	0.0	0.0	0.0
OTU176	*Ascomycota*	*Sordariomycetes*	*Hypocreales*	*Nectriaceae*	*Fusicolla*	*Fusicolla septimanifiniscientiae*	0.0	0.0	0.0	0.0	0.0	0.0	0.0	0.0	0.0	0.0	0.0	0.0
OTU177	*Ascomycota*	*Sordariomycetes*	*Hypocreales*	*Nectriaceae*	*Ilyonectria*	*Ilyonectria destructans*	0.0	0.0	0.0	0.0	0.0	0.0	0.0	0.7	0.6	0.0	0.0	0.0
OTU178	*Ascomycota*	*Sordariomycetes*	*Hypocreales*	*Nectriaceae*	*Ilyonectria*	unassigned	0.0	0.0	0.0	0.0	0.0	0.0	0.0	0.2	0.0	0.0	0.1	0.0
OTU179	*Ascomycota*	*Sordariomycetes*	*Hypocreales*	*Nectriaceae*	*Neocosmospora*	*Neocosmospora solani*	0.0	1.4	0.0	25.8	0.0	0.0	0.0	0.5	0.2	0.2	3.6	7.2
OTU180	*Ascomycota*	*Sordariomycetes*	*Hypocreales*	*Nectriaceae*	*Neonectria*	unidentified	0.0	0.0	0.0	0.0	0.0	0.0	0.0	0.0	0.0	0.0	0.0	0.0
OTU181	*Ascomycota*	*Sordariomycetes*	*Hypocreales*	*Nectriaceae*	*Ophionectria*	*Ophionectria trichospora*	0.5	0.0	0.0	0.0	0.0	0.0	0.0	0.0	0.0	0.0	0.0	0.0
OTU182	*Ascomycota*	*Sordariomycetes*	*Hypocreales*	*Nectriaceae*	*Paracremonium*	unidentified	0.0	0.0	0.0	0.0	0.0	0.0	0.0	0.0	2.4	0.0	0.0	0.0
OTU183	*Ascomycota*	*Sordariomycetes*	*Hypocreales*	*Nectriaceae*	unassigned	unassigned	0.0	0.0	0.0	0.0	0.0	0.0	0.0	0.0	0.0	0.0	0.0	0.0
OTU184	*Ascomycota*	*Sordariomycetes*	*Hypocreales*	*Nectriaceae*	*Volutella*	*unassigned*	0.0	0.0	0.0	0.0	0.0	0.0	0.0	0.0	0.0	0.0	0.0	0.0
OTU185	*Ascomycota*	*Sordariomycetes*	*Hypocreales*	*Nectriaceae*	*Volutella*	*Volutella ciliata*	0.0	0.0	0.0	0.0	0.0	0.0	0.0	0.1	0.0	0.0	0.0	0.0
OTU186	*Ascomycota*	*Sordariomycetes*	*Hypocreales*	*Ophiocordycipitaceae*	*Purpureocillium*	*Purpureocillium lilacinum*	0.0	0.0	0.0	0.0	0.0	0.0	0.0	0.0	0.0	0.0	0.1	0.1
OTU187	*Ascomycota*	*Sordariomycetes*	*Hypocreales*	*Ophiocordycipitaceae*	*Purpureocillium*	unidentified	0.0	0.0	0.0	21.5	0.0	0.0	0.0	0.0	0.0	0.0	0.1	0.0
OTU188	*Ascomycota*	*Sordariomycetes*	*Hypocreales*	*Ophiocordycipitaceae*	*Tolypocladium*	unidentified	0.0	0.0	0.0	0.0	0.0	0.0	0.0	0.0	0.8	0.0	0.0	0.0
OTU189	*Ascomycota*	*Sordariomycetes*	*Hypocreales*	*unassigned*	*unassigned*	unassigned	0.0	0.0	0.0	0.0	0.0	0.0	0.0	0.0	0.0	0.0	0.0	0.1
OTU190	*Ascomycota*	*Sordariomycetes*	*Microascales*	*Halosphaeriaceae*	*unidentified*	unidentified	0.0	0.0	0.0	0.0	0.0	0.0	0.0	0.0	0.0	0.0	0.0	0.0
OTU191	*Ascomycota*	*Sordariomycetes*	*Microascales*	*Microascaceae*	*Cephalotrichum*	*Cephalotrichum asperulum*	0.0	0.0	0.0	0.0	0.0	0.0	0.0	0.0	0.0	0.1	0.0	0.0
OTU192	*Ascomycota*	*Sordariomycetes*	*Microascales*	*Microascaceae*	*Cephalotrichum*	*Cephalotrichum microsporum*	0.0	33.1	0.0	17.8	0.0	0.0	36.2	15.0	0.0	69.1	47.4	67.0
OTU193	*Ascomycota*	*Sordariomycetes*	*Microascales*	*Microascaceae*	*Cephalotrichum*	*Cephalotrichum stemonitis*	0.0	0.0	0.0	0.0	0.0	0.0	0.0	0.0	0.0	0.0	0.0	0.0
OTU194	*Ascomycota*	*Sordariomycetes*	*Microascales*	*Microascaceae*	*Cephalotrichum*	unassigned	0.0	0.0	0.0	0.0	0.0	0.0	0.0	0.0	0.0	3.2	0.5	2.4
OTU195	*Ascomycota*	*Sordariomycetes*	*Microascales*	*Microascaceae*	*Cephalotrichum*	unidentified	0.0	0.0	0.0	0.5	0.0	0.0	0.0	0.1	0.0	0.0	0.0	0.0

OTU196	*Ascomycota*	*Sordariomycetes*	*Microascales*	*Microascaceae*	*Fairmania*	*Fairmania singularis*	0.0	0.0	0.0	0.0	0.0	0.0	0.0	0.0	0.1	0.0	0.0	0.0
OTU197	*Ascomycota*	*Sordariomycetes*	*Microascales*	*Microascaceae*	*Gamsia*	*Gamsia simplex*	0.0	0.0	0.0	0.4	0.0	0.0	0.0	5.7	1.0	0.0	0.0	0.0
OTU198	*Ascomycota*	*Sordariomycetes*	*Microascales*	*Microascaceae*	*Kernia*	*Kernia geniculotricha*	0.0	0.0	0.0	0.0	0.0	0.0	0.0	1.8	17.0	0.0	0.0	0.0
OTU199	*Ascomycota*	*Sordariomycetes*	*Microascales*	*Microascaceae*	*Mycochlamys*	*Mycochlamys macrospora*	0.0	0.0	0.0	0.0	0.0	0.0	0.0	0.3	2.5	0.0	0.0	0.0
OTU200	*Ascomycota*	*Sordariomycetes*	*Microascales*	*Microascaceae*	*Scopulariopsis*	*Scopulariopsis candida*	0.0	0.0	0.0	0.0	0.0	0.0	0.0	0.0	0.0	0.0	0.0	0.0
OTU201	*Ascomycota*	*Sordariomycetes*	*Microascales*	*unidentified*	*unidentified*	unidentified	0.0	0.0	0.0	0.0	0.0	0.0	0.0	0.0	0.0	0.0	0.0	0.0
OTU202	*Ascomycota*	*Sordariomycetes*	*Sordariales*	*Cephalothecaceae*	*unidentified*	unidentified	0.0	0.0	0.0	0.0	0.0	0.0	0.0	0.0	0.3	0.0	0.0	0.0
OTU203	*Ascomycota*	*Sordariomycetes*	*Sordariales*	*Chaetomiaceae*	*Botryotrichum*	*Botryotrichum spirotrichum*	0.0	0.0	0.0	0.0	0.0	0.0	0.0	1.1	0.0	0.0	0.0	0.0
OTU204	*Ascomycota*	*Sordariomycetes*	*Sordariales*	*Chaetomiaceae*	*Chaetomium*	unassigned	0.0	0.0	0.0	0.0	0.0	0.0	0.0	0.0	0.0	0.0	0.0	0.1
OTU205	*Ascomycota*	*Sordariomycetes*	*Sordariales*	*Chaetomiaceae*	*Chaetomium*	unidentified	0.8	0.0	0.0	0.1	0.0	0.0	0.0	0.1	0.0	0.0	0.0	0.0
OTU206	*Ascomycota*	*Sordariomycetes*	*Sordariales*	*Chaetomiaceae*	*Humicola*	*Humicola nigrescens*	0.0	0.0	0.0	0.0	0.0	0.0	0.0	2.6	5.1	0.0	0.0	1.0
OTU207	*Ascomycota*	*Sordariomycetes*	*Sordariales*	*Chaetomiaceae*	*Humicola*	unassigned	0.0	0.0	0.0	0.0	0.0	0.0	0.0	0.0	5.1	0.0	0.0	0.0
OTU208	*Ascomycota*	*Sordariomycetes*	*Sordariales*	*Chaetomiaceae*	*Trichocladium*	*Trichocladium opacum*	0.0	0.0	0.0	0.0	0.0	0.0	0.0	0.0	0.0	0.0	0.0	0.0
OTU209	*Ascomycota*	*Sordariomycetes*	*Sordariales*	*Chaetomiaceae*	*unassigned*	unassigned	0.0	0.0	0.0	0.0	0.0	0.0	0.0	0.9	0.8	0.0	0.0	0.0
OTU210	*Ascomycota*	*Sordariomycetes*	*Sordariales*	*Chaetomiaceae*	*unidentified*	unidentified	0.0	0.9	0.0	0.1	0.0	0.0	0.0	0.3	1.3	0.0	0.0	0.1
OTU211	*Ascomycota*	*Sordariomycetes*	*Sordariales*	*Lasiosphaeriaceae*	*Apodus*	unidentified	0.0	0.0	0.0	0.0	0.0	0.0	0.0	0.8	0.0	0.0	0.0	0.0
OTU212	*Ascomycota*	*Sordariomycetes*	*Sordariales*	*Lasiosphaeriaceae*	*Cercophora*	unidentified	0.0	0.0	0.0	0.0	0.0	0.0	0.0	0.1	1.8	0.0	0.0	0.0
OTU213	*Ascomycota*	*Sordariomycetes*	*Sordariales*	*Lasiosphaeriaceae*	*Podospora*	*Podospora intestinacea*	0.0	0.0	0.0	0.0	0.0	0.0	0.0	0.0	0.0	0.1	0.0	0.0
OTU214	*Ascomycota*	*Sordariomycetes*	*Sordariales*	*Lasiosphaeriaceae*	*Schizothecium*	*Schizothecium miniglutinans*	0.0	0.0	0.0	0.2	0.0	0.0	0.0	0.0	0.0	0.0	0.0	0.0
OTU215	*Ascomycota*	*Sordariomycetes*	*Sordariales*	*Sordariales fam Incertae sedis*	*Ramophialophora*	*Ramophialophora humicola*	0.0	0.0	0.0	0.0	0.0	0.0	0.0	0.0	1.2	0.0	0.0	0.0
OTU216	*Ascomycota*	*Sordariomycetes*	*Sordariales*	*Sordariales fam Incertae sedis*	*Staphylotrichum*	*Staphylotrichum boninense*	0.1	0.0	0.0	0.7	0.0	0.0	0.0	0.0	5.0	0.0	0.3	0.0
OTU217	*Ascomycota*	*Sordariomycetes*	*Sordariales*	*Sordariales fam Incertae sedis*	*Staphylotrichum*	unassigned	0.0	0.0	0.0	0.0	0.0	0.0	0.0	0.0	0.0	0.2	0.2	0.0
OTU218	*Ascomycota*	*Sordariomycetes*	*Sordariales*	*unassigned*	*unassigned*	unassigned	0.0	0.0	0.0	0.0	0.0	0.0	0.0	0.4	2.6	0.0	0.0	0.0
OTU219	*Ascomycota*	*Sordariomycetes*	*Sordariales*	*unidentified*	*unidentified*	unidentified	0.0	0.0	0.0	0.0	0.0	0.0	0.0	0.0	0.0	0.0	0.0	0.0
OTU220	*Ascomycota*	*Sordariomycetes*	*unassigned*	*unassigned*	*unassigned*	unassigned	0.0	0.0	0.0	0.0	0.0	0.0	0.0	0.1	0.5	0.0	0.0	0.1
OTU221	*Ascomycota*	*Sordariomycetes*	*unidentified*	*unidentified*	*unidentified*	unidentified	0.0	0.0	0.0	0.0	0.0	0.0	0.1	0.0	0.0	0.0	0.0	0.0
OTU222	*Ascomycota*	*Sordariomycetes*	*Xylariales*	*Coniocessiaceae*	*Coniocessia*	*Coniocessia maxima*	0.0	0.0	0.0	0.0	0.0	0.0	0.0	0.0	0.0	0.0	0.0	0.0
OTU223	*Ascomycota*	*Sordariomycetes*	*Xylariales*	*Microdochiaceae*	*Idriella*	*Idriella lunata*	0.0	0.0	0.0	0.0	0.0	0.0	0.0	0.0	0.0	0.0	0.0	0.0
OTU224	*Ascomycota*	*Sordariomycetes*	*Xylariales*	*Xylariaceae*	*Hypoxylon*	*Hypoxylon ticinense*	0.0	0.0	0.0	0.0	0.0	0.0	0.0	0.0	0.0	0.0	0.0	0.0
OTU225	*Ascomycota*	*Sordariomycetes*	*Xylariales*	*Xylariaceae*	*Rosellinia*	*Rosellinia mammiformis*	0.0	0.0	0.0	0.0	0.0	0.0	0.0	0.0	0.0	0.0	0.0	0.0
OTU226	*Ascomycota*	*Sordariomycetes*	*Xylariales*	*Xylariaceae*	*Virgaria*	*Virgaria nigra*	0.0	0.0	0.0	0.0	0.0	0.0	0.1	0.4	3.0	0.0	0.0	0.0
OTU227	*Ascomycota*	*Sordariomycetes*	*Xylariales*	*Xylariaceae*	*Xylaria*	unassigned	0.0	0.0	0.0	0.0	0.0	0.0	0.0	0.0	0.0	0.0	0.0	0.0
OTU228	*Ascomycota*	*Sordariomycetes*	*Xylariales*	*Xylariaceae*	*Zygosporium*	*Zygosporium pseudogibbum*	0.0	0.0	0.0	0.0	0.0	0.0	0.0	0.0	0.0	0.0	0.0	0.0

OTU229	*Ascomycota*	*Sordariomycetes*	*Xylariales*	*Xylariales fam Incertae sedis*	*Hansfordia*	*Hansfordia pulvinata*	0.0	0.0	0.0	0.0	0.0	0.0	0.0	0.0	0.0	0.0	0.0	0.0
OTU230	*Ascomycota*	unassigned	unassigned	unassigned	unassigned	unassigned	0.0	0.0	0.0	0.0	0.0	0.0	0.6	1.3	2.3	0.0	2.5	2.0
OTU231	*Ascomycota*	unidentified	unidentified	unidentified	unidentified	unidentified	0.0	0.0	0.0	0.7	0.0	0.0	0.0	0.2	0.0	0.0	0.0	0.0
OTU232	*Basidiomycota*	*Agaricomycetes*	*Agaricales*	*Agaricaceae*	*Lepiota*	*Lepiota lilacea*	0.0	0.0	0.0	0.0	0.0	0.0	0.0	0.0	0.0	0.0	0.0	0.0
OTU233	*Basidiomycota*	*Agaricomycetes*	*Agaricales*	*Agaricaceae*	*Lepiota*	*Lepiota perplexa*	0.0	0.0	0.0	0.0	0.0	0.0	0.0	0.0	0.0	0.0	0.0	0.0
OTU234	*Basidiomycota*	*Agaricomycetes*	*Agaricales*	*Agaricaceae*	*Melanophyllum*	*Melanophyllum haematospermum*	0.0	0.0	0.0	0.0	0.0	0.0	0.0	0.0	0.0	0.0	0.0	0.0
OTU235	*Basidiomycota*	*Agaricomycetes*	*Agaricales*	*Agaricaceae*	*Tulostoma*	*Tulostoma melanocyclum*	0.0	0.0	0.0	0.0	0.0	0.0	0.0	0.0	0.7	0.0	0.0	0.0
OTU236	*Basidiomycota*	*Agaricomycetes*	*Agaricales*	*Agaricales fam Incertae sedis*	*Uncobasidium*	unidentified	0.0	0.0	0.0	0.0	0.0	0.0	0.0	0.0	0.0	0.0	0.0	0.0
OTU237	*Basidiomycota*	*Agaricomycetes*	*Agaricales*	*Bolbitiaceae*	*Descomyces*	unassigned	0.0	0.0	0.0	0.0	0.0	0.0	0.0	0.0	0.0	0.0	0.0	0.0
OTU238	*Basidiomycota*	*Agaricomycetes*	*Agaricales*	*Clavariaceae*	*Clavaria*	*Clavaria californica*	0.0	0.0	0.0	0.0	0.0	0.0	0.0	0.0	0.0	0.0	0.0	0.0
OTU239	*Basidiomycota*	*Agaricomycetes*	*Agaricales*	*Clavariaceae*	*Clavulinopsis*	*Clavulinopsis hisingeri*	0.0	0.0	0.0	0.0	0.0	0.0	0.0	0.0	0.0	0.0	0.0	0.0
OTU240	*Basidiomycota*	*Agaricomycetes*	*Agaricales*	*Clavariaceae*	unassigned	unassigned	0.0	0.0	0.0	0.0	0.0	0.0	0.0	0.0	0.0	0.0	0.0	0.0
OTU241	*Basidiomycota*	*Agaricomycetes*	*Agaricales*	*Clavariaceae*	unidentified	unidentified	0.0	0.0	0.0	0.0	0.0	0.0	0.5	0.0	0.0	0.0	0.0	0.0
OTU242	*Basidiomycota*	*Agaricomycetes*	*Agaricales*	*Crepidotaceae*	*Crepidotus*	*Crepidotus crocophyllus*	0.0	0.0	0.0	0.0	0.0	0.0	0.0	0.0	0.0	0.0	0.0	0.0
OTU243	*Basidiomycota*	*Agaricomycetes*	*Agaricales*	*Cystostereaceae*	*Crustomyces*	*Crustomyces subabruptus*	0.0	0.0	0.0	0.0	0.0	0.0	0.0	0.1	0.0	0.0	0.0	0.0
OTU244	*Basidiomycota*	*Agaricomycetes*	*Agaricales*	*Entolomataceae*	*Clitopilus*	unidentified	0.0	0.0	0.0	0.0	0.0	0.0	0.0	0.1	0.0	0.0	0.0	0.0
OTU245	*Basidiomycota*	*Agaricomycetes*	*Agaricales*	*Entolomataceae*	*Entoloma*	unassigned	0.0	0.0	0.0	0.0	0.0	0.0	0.0	0.0	0.0	0.0	0.0	0.0
OTU246	*Basidiomycota*	*Agaricomycetes*	*Agaricales*	*Hebelomataceae*	unidentified	unidentified	0.0	0.0	0.0	0.0	0.0	0.0	0.1	0.0	0.0	0.0	0.0	0.0
OTU247	*Basidiomycota*	*Agaricomycetes*	*Agaricales*	*Hymenogastraceae*	*Hymenogaster*	*Hymenogaster griseus*	0.0	0.0	0.0	0.0	0.0	0.0	0.0	0.0	0.0	0.0	0.0	0.0
OTU248	*Basidiomycota*	*Agaricomycetes*	*Agaricales*	*Hymenogastraceae*	*Hymenogaster*	*Hymenogaster olivaceus*	0.0	0.0	0.0	0.0	0.0	0.0	0.0	0.1	0.0	0.0	0.0	0.0
OTU249	*Basidiomycota*	*Agaricomycetes*	*Agaricales*	*Inocybaceae*	*Inocybe*	*Inocybe griseovelata*	0.0	0.0	0.0	0.0	0.0	0.0	0.2	0.0	0.0	0.0	0.0	0.0
OTU250	*Basidiomycota*	*Agaricomycetes*	*Agaricales*	*Inocybaceae*	*Inocybe*	*Inocybe pusio*	0.0	0.0	0.0	0.0	0.0	0.0	1.5	0.0	0.0	0.0	0.0	0.0
OTU251	*Basidiomycota*	*Agaricomycetes*	*Agaricales*	*Lycoperdaceae*	*Lycoperdon*	unassigned	0.0	0.0	0.0	0.0	0.0	0.0	0.0	0.0	0.0	0.0	0.0	0.0
OTU252	*Basidiomycota*	*Agaricomycetes*	*Agaricales*	*Lycoperdaceae*	*unassigned*	unassigned	0.0	0.0	0.0	0.0	0.0	0.0	0.0	0.0	0.9	0.0	0.0	0.0
OTU253	*Basidiomycota*	*Agaricomycetes*	*Agaricales*	*Marasmiaceae*	*Chaetocalathus*	unassigned	0.0	0.0	0.0	0.0	0.0	0.0	0.0	0.0	0.0	0.0	0.0	0.0
OTU254	*Basidiomycota*	*Agaricomycetes*	*Agaricales*	*Omphalotaceae*	*Marasmiellus*	*Marasmiellus candidus*	0.0	0.0	0.0	0.0	0.0	0.0	0.0	0.0	0.0	0.0	0.0	0.0
OTU255	*Basidiomycota*	*Agaricomycetes*	*Agaricales*	*Omphalotaceae*	*Omphalotus*	*Omphalotus olearius*	0.0	18.2	0.0	0.0	0.0	0.0	0.0	0.0	0.0	0.0	0.0	0.0
OTU256	*Basidiomycota*	*Agaricomycetes*	*Agaricales*	*Pluteaceae*	*Pluteus*	*Pluteus diettrichii*	0.0	0.0	0.0	0.0	0.0	0.0	0.0	0.0	0.0	0.0	0.0	0.0
OTU257	*Basidiomycota*	*Agaricomycetes*	*Agaricales*	*Pluteaceae*	*Pluteus*	unidentified	0.0	0.0	0.0	0.0	0.0	0.0	0.2	0.0	0.0	0.0	0.0	0.0
OTU258	*Basidiomycota*	*Agaricomycetes*	*Agaricales*	*Psathyrellaceae*	*Coprinellus*	*Coprinellus disseminatus*	0.0	0.0	0.0	0.0	0.0	0.0	0.0	0.0	0.0	0.0	0.0	0.0
OTU259	*Basidiomycota*	*Agaricomycetes*	*Agaricales*	*Psathyrellaceae*	*Coprinellus*	*Coprinellus micaceus*	0.0	0.0	0.0	0.0	0.0	0.0	0.1	0.5	0.0	0.0	0.0	0.0
OTU260	*Basidiomycota*	*Agaricomycetes*	*Agaricales*	*Pterulaceae*	*Aphanobasidium*	*Aphanobasidium pseudotsugae*	0.0	0.0	0.0	0.0	0.0	0.0	0.0	0.0	0.0	0.0	0.0	0.0
OTU261	*Basidiomycota*	*Agaricomycetes*	*Agaricales*	*Stephanosporaceae*	*Stephanospora*	*Stephanospora caroticolor*	0.0	0.0	0.0	0.0	0.0	0.0	0.0	0.0	0.0	0.0	0.0	0.0

OTU262	*Basidiomycota*	*Agaricomycetes*	*Agaricales*	*Strophariaceae*	*Hypholoma*	*Hypholoma fasciculare*	0.0	0.0	0.0	0.0	0.0	0.0	0.0	0.0	0.0	0.0	0.0	0.0
OTU263	*Basidiomycota*	*Agaricomycetes*	*Agaricales*	*Strophariaceae*	*Tubaria*	*Tubaria conspersa*	0.0	0.0	0.0	0.0	0.0	0.0	0.0	0.0	0.0	0.0	0.0	0.0
OTU264	*Basidiomycota*	*Agaricomycetes*	*Agaricales*	*Tricholomataceae*	*Clitocybe*	*Clitocybe nebularis*	0.0	0.0	0.0	0.0	0.0	0.0	0.0	0.0	0.0	0.0	0.0	0.0
OTU265	*Basidiomycota*	*Agaricomycetes*	*Agaricales*	*Tricholomataceae*	*Dermoloma*	*Dermoloma cuneifolium*	0.0	0.0	0.0	0.0	0.0	0.0	0.6	0.0	0.0	0.0	0.0	0.0
OTU266	*Basidiomycota*	*Agaricomycetes*	*Agaricales*	*Tricholomataceae*	*Dermoloma*	unidentified	0.0	0.0	0.0	0.0	0.0	0.0	0.0	0.0	0.0	0.0	0.0	0.0
OTU267	*Basidiomycota*	*Agaricomycetes*	*Agaricales*	*Tricholomataceae*	*Hemimycena*	*Hemimycena pseudolactea*	0.0	0.0	0.0	0.0	0.0	0.0	0.0	0.0	0.0	0.0	0.0	0.0
OTU268	*Basidiomycota*	*Agaricomycetes*	*Agaricales*	*Tricholomataceae*	*Hemimycena*	*unassigned*	0.0	0.0	0.0	0.0	0.0	0.0	0.0	0.0	0.0	0.0	0.0	0.0
OTU269	*Basidiomycota*	*Agaricomycetes*	*Agaricales*	*Tricholomataceae*	*Paralepista*	*Paralepista flaccida*	0.0	0.0	0.0	0.0	0.0	0.0	0.0	0.0	0.0	0.0	0.0	0.0
OTU270	*Basidiomycota*	*Agaricomycetes*	*Agaricales*	*Typhulaceae*	*Tygervalleyomyces*	unassigned	0.0	0.0	0.0	0.0	0.0	0.0	0.0	0.0	0.0	0.0	0.0	0.0
OTU271	*Basidiomycota*	*Agaricomycetes*	*Agaricales*	*Typhulaceae*	*Typhula*	*Typhula spathulata*	0.0	0.0	0.0	0.0	0.0	0.0	0.0	0.0	0.0	0.0	0.0	0.0
OTU272	*Basidiomycota*	*Agaricomycetes*	*Agaricales*	unassigned	unassigned	unassigned	0.0	0.0	0.0	0.0	0.0	0.0	0.2	0.5	0.0	0.0	0.0	0.0
OTU273	*Basidiomycota*	*Agaricomycetes*	*Agaricales*	unidentified	unidentified	unidentified	0.0	0.0	0.0	0.0	0.0	0.0	1.4	0.0	0.0	0.0	0.0	0.0
OTU274	*Basidiomycota*	*Agaricomycetes*	*Agaricomycetes ord Incertae sedis*	*Agaricomycetes fam Incertae sedis*	*Xenasmatella*	unassigned	0.0	0.0	0.0	0.0	0.0	0.0	0.0	0.3	0.0	0.0	0.0	0.0
OTU275	*Basidiomycota*	*Agaricomycetes*	*Agaricomycetes ord Incertae sedis*	*Agaricomycetes fam Incertae sedis*	*Xenasmatella*	unidentified	0.0	0.0	0.0	0.0	0.0	0.0	0.0	0.0	0.0	0.0	0.0	0.0
OTU276	*Basidiomycota*	*Agaricomycetes*	*Agaricomycetes ord Incertae sedis*	*Agaricomycetes fam Incertae sedis*	*Xenasmatella*	*Xenasmatella ardosiaca*	0.0	0.0	0.0	0.0	0.0	0.0	0.0	0.2	0.0	0.0	0.0	0.0
OTU277	*Basidiomycota*	*Agaricomycetes*	*Atheliales*	*Atheliaceae*	*Athelia*	unidentified	0.0	0.0	0.0	0.0	0.0	0.0	0.0	0.0	0.0	0.0	0.0	0.0
OTU278	*Basidiomycota*	*Agaricomycetes*	*Auriculariales*	*Auriculariaceae*	*Auricularia*	unidentified	0.0	0.0	0.0	0.0	0.0	0.0	0.0	0.0	0.0	0.0	0.0	0.0
OTU279	*Basidiomycota*	*Agaricomycetes*	*Auriculariales*	*Exidiaceae*	*Exidia*	*Exidia japonica*	0.0	0.0	0.0	0.0	0.0	0.0	0.0	0.0	0.0	0.0	0.0	0.0
OTU280	*Basidiomycota*	*Agaricomycetes*	*Auriculariales*	*Hyaloriaceae*	*Myxarium*	unassigned	0.0	0.0	0.0	0.0	0.0	0.0	0.0	0.0	0.0	0.0	0.0	0.0
OTU281	*Basidiomycota*	*Agaricomycetes*	*Auriculariales*	*Hyaloriaceae*	*Myxarium*	unidentified	0.0	0.0	0.0	0.0	0.0	0.0	0.0	0.0	0.0	0.0	0.0	0.0
OTU282	*Basidiomycota*	*Agaricomycetes*	*Auriculariales*	*Hyaloriaceae*	*Protomerulius*	unidentified	0.0	0.0	0.0	0.0	0.0	0.0	0.0	0.0	0.0	0.0	0.0	0.0
OTU283	*Basidiomycota*	*Agaricomycetes*	*Auriculariales*	*Hyaloriaceae*	*Stypella*	*Stypella grilletii*	0.0	0.0	0.0	0.0	0.0	0.0	0.0	0.0	0.0	0.0	0.0	0.0
OTU284	*Basidiomycota*	*Agaricomycetes*	*Auriculariales*	unassigned	unassigned	unassigned	0.0	0.0	0.0	0.0	0.0	0.0	0.0	0.0	0.0	0.0	0.0	0.0
OTU285	*Basidiomycota*	*Agaricomycetes*	*Auriculariales*	unidentified	unidentified	unidentified	0.0	0.0	0.0	0.0	0.0	0.0	0.0	0.0	0.0	0.0	0.0	0.0
OTU286	*Basidiomycota*	*Agaricomycetes*	*Boletales*	*Boletaceae*	*Suillellus*	*Suillellus queletii*	0.0	0.0	0.0	0.0	0.0	0.0	0.0	0.0	0.0	0.0	0.0	0.0
OTU287	*Basidiomycota*	*Agaricomycetes*	*Boletales*	*Melanogastraceae*	*Melanogaster*	unassigned	0.0	0.0	0.0	0.0	0.0	0.0	0.0	0.0	0.0	0.0	0.0	0.0
OTU288	*Basidiomycota*	*Agaricomycetes*	*Boletales*	*Suillaceae*	*Suillus*	*Suillus pseudobrevipes*	0.0	0.0	0.0	0.0	0.0	0.0	0.0	0.0	0.0	0.0	0.0	0.0
OTU289	*Basidiomycota*	*Agaricomycetes*	*Cantharellales*	*Cantharellales fam Incertae sedis*	*Burgoa*	*Burgoa verzuoliana*	0.0	0.0	0.0	0.0	0.0	0.0	0.0	0.0	0.0	0.0	0.0	0.0
OTU290	*Basidiomycota*	*Agaricomycetes*	*Cantharellales*	*Cantharellales fam Incertae sedis*	*Sistotrema*	*Sistotrema brinkmannii*	0.0	0.0	0.0	0.0	0.0	0.0	0.0	0.0	0.0	0.0	0.0	0.0
OTU291	*Basidiomycota*	*Agaricomycetes*	*Cantharellales*	*Cantharellales fam Incertae sedis*	*Sistotrema*	*Sistotrema oblongisporum*	0.0	0.0	0.0	0.0	27.3	0.0	0.0	0.0	0.0	0.0	0.0	0.0
OTU292	*Basidiomycota*	*Agaricomycetes*	*Cantharellales*	*Cantharellales fam Incertae sedis*	*Sistotrema*	unidentified	0.0	0.0	0.0	0.0	0.0	0.0	0.0	0.2	0.0	0.0	0.0	0.0
OTU293	*Basidiomycota*	*Agaricomycetes*	*Cantharellales*	*Cantharellales fam Incertae sedis*	unidentified	unidentified	0.0	0.0	0.0	0.0	0.0	0.0	0.0	0.1	0.0	0.0	0.0	0.0
OTU294	*Basidiomycota*	*Agaricomycetes*	*Cantharellales*	*Ceratobasidiaceae*	*Ceratobasidium*	unidentified	0.0	0.0	0.0	0.0	0.0	0.0	0.1	0.0	0.0	0.0	0.0	0.0

OTU295	*Basidiomycota*	*Agaricomycetes*	*Cantharellales*	*Ceratobasidiaceae*	unassigned	unassigned	0.0	0.0	0.0	0.0	0.0	0.0	0.0	0.0	0.0	0.0	0.0	0.0
OTU296	*Basidiomycota*	*Agaricomycetes*	*Cantharellales*	*Tulasnellaceae*	unidentified	unidentified	0.0	0.0	0.0	0.0	0.0	0.0	0.0	0.0	0.0	0.0	0.0	0.0
OTU297	*Basidiomycota*	*Agaricomycetes*	*Corticiales*	*Corticiaceae*	*Corticium*	unidentified	0.0	0.0	0.0	0.0	0.0	0.0	0.0	3.2	0.0	0.0	0.0	0.0
OTU298	*Basidiomycota*	*Agaricomycetes*	*Corticiales*	unidentified	unidentified	unidentified	0.0	0.0	0.0	0.0	0.0	0.0	0.0	0.0	0.0	0.0	0.0	0.0
OTU299	*Basidiomycota*	*Agaricomycetes*	*Gomphales*	*Gomphaceae*	*Ramaria*	*Ramaria pinicola*	0.0	0.0	0.0	0.0	0.0	0.0	0.0	0.0	0.0	0.0	0.0	0.0
OTU300	*Basidiomycota*	*Agaricomycetes*	*Hymenochaetales*	*Hymenochaetaceae*	*Fomitiporella*	unidentified	6.6	0.0	0.0	0.0	0.0	0.0	0.0	0.0	0.0	0.0	0.0	0.0
OTU301	*Basidiomycota*	*Agaricomycetes*	*Hymenochaetales*	*Hymenochaetaceae*	*Fuscoporia*	*Fuscoporia torulosa*	0.0	0.0	0.0	0.0	0.0	0.0	0.0	0.0	0.0	0.0	0.0	0.0
OTU302	*Basidiomycota*	*Agaricomycetes*	*Hymenochaetales*	*Hymenochaetaceae*	*Phylloporia*	unidentified	0.0	0.0	0.0	0.0	0.0	0.0	0.0	0.3	0.0	0.0	0.0	0.0
OTU303	*Basidiomycota*	*Agaricomycetes*	*Hymenochaetales*	*Hymenochaetaceae*	unassigned	unassigned	0.0	0.0	0.0	0.0	0.0	0.0	0.0	0.0	0.0	0.1	0.0	0.0
OTU304	*Basidiomycota*	*Agaricomycetes*	*Hymenochaetales*	*Hymenochaetales fam Incertae sedis*	*Peniophorella*	*Peniophorella praetermissa*	0.0	0.0	0.0	0.0	0.0	0.0	0.0	0.0	0.0	0.0	0.0	0.0
OTU305	*Basidiomycota*	*Agaricomycetes*	*Hymenochaetales*	*Hymenochaetales fam Incertae sedis*	*Peniophorella*	*Peniophorella pubera*	0.0	0.0	0.0	0.0	0.0	0.0	0.0	0.0	0.0	0.0	0.0	0.0
OTU306	*Basidiomycota*	*Agaricomycetes*	*Hymenochaetales*	*Hymenochaetales fam Incertae sedis*	*Resinicium*	*Resinicium bicolor*	0.0	0.0	0.0	0.0	0.0	0.0	0.0	0.0	0.0	0.0	0.0	0.0
OTU307	*Basidiomycota*	*Agaricomycetes*	*Hymenochaetales*	*Hymenochaetales fam Incertae sedis*	*Resinicium*	*Resinicium friabile*	0.0	0.0	0.0	0.0	0.0	0.0	0.0	0.6	0.0	0.0	0.0	0.0
OTU308	*Basidiomycota*	*Agaricomycetes*	*Hymenochaetales*	*Rickenellaceae*	*Sidera*	unidentified	0.0	0.0	0.0	0.0	0.0	0.0	0.0	0.0	0.0	0.0	0.0	0.0
OTU309	*Basidiomycota*	*Agaricomycetes*	*Hymenochaetales*	*Schizoporaceae*	*Hyphodontia*	unidentified	0.0	0.0	0.0	0.0	0.0	0.0	0.0	0.7	0.0	0.0	0.0	0.0
OTU310	*Basidiomycota*	*Agaricomycetes*	*Hymenochaetales*	*Schizoporaceae*	*Lyomyces*	*Lyomyces mascarensis*	0.0	0.0	0.0	0.0	0.0	0.0	0.0	0.0	0.0	0.0	0.0	0.0
OTU311	*Basidiomycota*	*Agaricomycetes*	*Hymenochaetales*	*Schizoporaceae*	*Lyomyces*	unidentified	0.0	0.0	0.0	0.0	0.0	0.0	0.0	1.3	0.0	0.0	0.0	0.0
OTU312	*Basidiomycota*	*Agaricomycetes*	*Hymenochaetales*	*Schizoporaceae*	unassigned	unassigned	0.0	0.0	0.0	0.0	0.0	0.0	0.0	0.3	0.0	0.0	0.0	0.0
OTU313	*Basidiomycota*	*Agaricomycetes*	*Hymenochaetales*	*Schizoporaceae*	*Xylodon*	*Xylodon flaviporus*	0.0	0.0	0.0	0.0	0.0	0.0	0.0	0.1	0.0	0.0	0.0	0.0
OTU314	*Basidiomycota*	*Agaricomycetes*	*Hymenochaetales*	*Schizoporaceae*	*Xylodon*	*Xylodon raduloides*	0.0	0.0	0.0	0.0	0.0	0.0	0.0	0.0	0.0	0.0	0.0	0.0
OTU315	*Basidiomycota*	*Agaricomycetes*	*Hymenochaetales*	*Schizoporaceae*	*Xylodon*	*Xylodon sambuci*	0.0	0.0	0.0	0.0	0.0	0.0	0.0	0.0	0.0	0.0	0.0	0.0
OTU316	*Basidiomycota*	*Agaricomycetes*	*Phallales*	*Phallaceae*	*Phallus*	*Phallus impudicus*	0.0	0.0	0.0	0.0	0.0	0.0	0.0	0.0	0.0	0.0	0.0	0.0
OTU317	*Basidiomycota*	*Agaricomycetes*	*Polyporales*	*Fomitopsidaceae*	*Auriporia*	*Auriporia aurea*	0.0	0.0	0.0	0.0	0.0	0.0	0.0	0.0	0.0	0.0	0.0	0.0
OTU318	*Basidiomycota*	*Agaricomycetes*	*Polyporales*	*Fomitopsidaceae*	*Postia*	*Postia hibernica*	0.0	0.0	0.0	0.0	0.0	0.0	0.0	0.0	0.0	0.0	0.0	0.0
OTU319	*Basidiomycota*	*Agaricomycetes*	*Polyporales*	*Fomitopsidaceae*	*Skeletocutis*	*Skeletocutis nivea*	0.0	0.0	0.0	0.0	0.0	0.0	0.0	0.3	0.0	0.0	0.0	0.0
OTU320	*Basidiomycota*	*Agaricomycetes*	*Polyporales*	*Ganodermataceae*	*Ganoderma*	*Ganoderma adspersum*	0.0	0.0	0.0	0.0	0.0	0.0	0.0	0.2	0.0	0.0	0.0	0.0
OTU321	*Basidiomycota*	*Agaricomycetes*	*Polyporales*	*Irpicaceae*	*Byssomerulius*	*Byssomerulius corium*	0.0	0.0	0.0	0.0	0.0	0.0	0.0	0.0	0.0	0.0	0.0	0.0
OTU322	*Basidiomycota*	*Agaricomycetes*	*Polyporales*	*Irpicaceae*	*Ceriporia*	*Ceriporia humilis*	0.0	0.0	0.0	0.0	0.0	0.0	0.0	0.0	0.0	0.0	0.0	0.0
OTU323	*Basidiomycota*	*Agaricomycetes*	*Polyporales*	*Irpicaceae*	*Ceriporia*	*Ceriporia lacerata*	0.0	0.0	0.0	0.0	0.0	0.0	0.0	0.0	0.0	0.0	0.0	0.0
OTU324	*Basidiomycota*	*Agaricomycetes*	*Polyporales*	*Irpicaceae*	*Ceriporia*	*Ceriporia viridans*	0.0	0.0	0.0	0.0	0.0	0.0	0.0	0.0	0.0	0.0	0.0	0.0
OTU325	*Basidiomycota*	*Agaricomycetes*	*Polyporales*	*Meruliaceae*	*Mycoacia*	*Mycoacia aurea*	0.0	0.0	0.0	0.0	0.0	0.0	0.0	0.0	0.0	0.0	0.0	0.0
OTU326	*Basidiomycota*	*Agaricomycetes*	*Polyporales*	*Meruliaceae*	*Mycoacia*	*Mycoacia fuscoatra*	0.0	0.0	0.0	0.0	0.0	0.0	0.0	0.0	0.0	0.0	0.0	0.0
OTU327	*Basidiomycota*	*Agaricomycetes*	*Polyporales*	*Meruliaceae*	*Mycoacia*	*Mycoacia uda*	0.0	0.0	0.0	0.0	0.0	0.0	0.0	0.0	0.0	0.0	0.0	0.0

OTU328	*Basidiomycota*	*Agaricomycetes*	*Polyporales*	*Meruliaceae*	*Mycoacia*	unidentified	0.0	0.0	0.0	0.0	0.0	0.0	0.0	0.0	0.0	0.0	0.0	0.0
OTU329	*Basidiomycota*	*Agaricomycetes*	*Polyporales*	*Meruliaceae*	*Phlebia*	*Phlebia rufa*	0.0	0.0	0.0	0.0	0.0	0.0	0.0	0.1	0.0	0.0	0.0	0.0
OTU330	*Basidiomycota*	*Agaricomycetes*	*Polyporales*	*Meruliaceae*	*Phlebia*	*Phlebia tuberculata*	0.0	0.0	0.0	0.0	0.0	0.0	0.0	0.0	0.0	0.0	0.0	0.0
OTU331	*Basidiomycota*	*Agaricomycetes*	*Polyporales*	*Meruliaceae*	*Scopuloides*	*Scopuloides hydnoides*	0.0	0.0	0.0	0.0	0.0	0.0	0.0	0.0	0.0	0.0	0.0	0.0
OTU332	*Basidiomycota*	*Agaricomycetes*	*Polyporales*	*Meruliaceae*	*Scopuloides*	unassigned	0.0	0.0	0.0	0.0	0.0	0.0	0.0	0.0	0.0	0.0	0.0	0.0
OTU333	*Basidiomycota*	*Agaricomycetes*	*Polyporales*	*Phanerochaetaceae*	*Bjerkandera*	*Bjerkandera adusta*	0.0	0.0	0.0	0.0	0.0	0.0	0.0	0.0	0.0	0.0	0.0	0.0
OTU334	*Basidiomycota*	*Agaricomycetes*	*Polyporales*	*Phanerochaetaceae*	*Hyphodermella*	*Hyphodermella corrugata*	0.0	0.0	0.0	0.0	0.0	0.0	0.0	0.1	0.0	0.0	0.0	0.0
OTU335	*Basidiomycota*	*Agaricomycetes*	*Polyporales*	*Phanerochaetaceae*	*Hyphodermella*	unidentified	0.0	0.0	0.0	0.0	0.0	0.0	0.0	0.0	0.0	0.0	0.0	0.0
OTU336	*Basidiomycota*	*Agaricomycetes*	*Polyporales*	*Phanerochaetaceae*	*Phaeophlebiopsis*	*Phaeophlebiopsis ignerii*	0.0	0.0	0.0	0.0	0.0	0.0	0.0	0.3	0.0	0.0	0.0	0.0
OTU337	*Basidiomycota*	*Agaricomycetes*	*Polyporales*	*Phanerochaetaceae*	*Phanerochaete*	*Phanerochaete sanguineocarnosa*	0.0	0.0	0.0	0.0	0.0	0.0	0.0	0.0	0.0	0.0	0.0	0.0
OTU338	*Basidiomycota*	*Agaricomycetes*	*Polyporales*	*Phanerochaetaceae*	*Phanerochaete*	unassigned	0.0	0.0	0.0	0.0	0.0	0.0	0.0	0.0	0.0	0.0	0.0	0.0
OTU339	*Basidiomycota*	*Agaricomycetes*	*Polyporales*	*Phanerochaetaceae*	*Terana*	*Terana coerulea*	0.0	0.0	0.0	0.0	0.0	0.0	0.0	0.0	0.0	0.0	0.0	0.0
OTU340	*Basidiomycota*	*Agaricomycetes*	*Polyporales*	*Podoscyphaceae*	*Abortiporus*	*Abortiporus biennis*	0.0	0.0	0.0	0.0	0.0	0.0	0.0	0.0	0.0	0.0	0.0	0.0
OTU341	*Basidiomycota*	*Agaricomycetes*	*Polyporales*	*Polyporaceae*	*Daedaleopsis*	unassigned	0.0	0.0	0.0	0.0	0.0	0.0	0.0	0.0	0.0	0.0	0.0	0.0
OTU342	*Basidiomycota*	*Agaricomycetes*	*Polyporales*	*Polyporaceae*	*Trametes*	*Trametes versicolor*	0.0	0.0	0.0	0.0	0.0	0.0	0.0	1.5	0.0	0.0	0.0	0.0
OTU343	*Basidiomycota*	*Agaricomycetes*	*Polyporales*	*Steccherinaceae*	*Odonticium*	*Odonticium septocystidia*	0.0	0.0	0.0	0.0	0.0	0.0	0.0	0.0	0.0	0.0	0.0	0.0
OTU344	*Basidiomycota*	*Agaricomycetes*	*Polyporales*	*Steccherinaceae*	*Steccherinum*	*Steccherinum fimbriatum*	0.0	0.0	0.0	0.0	0.0	0.0	0.0	0.0	0.0	0.0	0.0	0.0
OTU345	*Basidiomycota*	*Agaricomycetes*	*Polyporales*	*Steccherinaceae*	*Steccherinum*	*Steccherinum ochraceum*	0.0	0.0	0.0	0.0	0.0	0.0	0.0	0.0	0.0	0.0	0.0	0.0
OTU346	*Basidiomycota*	*Agaricomycetes*	*Polyporales*	unassigned	unassigned	unassigned	0.0	0.0	0.0	0.0	0.0	0.0	0.0	0.0	0.0	0.0	0.0	0.0
OTU347	*Basidiomycota*	*Agaricomycetes*	*Polyporales*	*Xenasmataceae*	*Xenasma*	*Xenasma rimicola*	0.0	0.0	0.0	0.0	0.0	0.0	0.0	0.2	0.0	0.0	0.0	0.0
OTU348	*Basidiomycota*	*Agaricomycetes*	*Russulales*	*Bondarzewiaceae*	*Heterobasidion*	unassigned	0.0	0.0	0.0	0.0	0.0	0.0	0.0	0.0	0.0	0.0	0.0	0.0
OTU349	*Basidiomycota*	*Agaricomycetes*	*Russulales*	*Lachnocladiaceae*	*Gloiothele*	unidentified	0.0	0.0	0.0	0.0	0.0	0.0	0.0	0.0	0.0	0.0	0.0	0.0
OTU350	*Basidiomycota*	*Agaricomycetes*	*Russulales*	*Lachnocladiaceae*	*Vararia*	*Vararia gallica*	0.0	0.0	0.0	0.0	0.0	0.0	0.0	0.0	0.0	0.0	0.0	0.0
OTU351	*Basidiomycota*	*Agaricomycetes*	*Russulales*	*Peniophoraceae*	*Peniophora*	*Peniophora cinerea*	0.0	0.0	0.0	0.0	0.0	0.0	0.0	0.5	0.0	0.0	0.0	0.0
OTU352	*Basidiomycota*	*Agaricomycetes*	*Russulales*	*Peniophoraceae*	*Peniophora*	*Peniophora limitata*	0.0	0.0	0.0	0.0	0.0	0.0	0.0	0.1	0.0	0.0	0.0	0.0
OTU353	*Basidiomycota*	*Agaricomycetes*	*Russulales*	*Peniophoraceae*	*Peniophora*	*Peniophora quercina*	0.0	0.0	0.0	0.0	0.0	0.0	0.0	0.0	0.0	0.0	0.0	0.0
OTU354	*Basidiomycota*	*Agaricomycetes*	*Russulales*	*Peniophoraceae*	*Peniophora*	*Peniophora rufomarginata*	0.0	0.0	0.0	0.0	0.0	0.0	0.0	0.1	0.0	0.0	0.0	0.0
OTU355	*Basidiomycota*	*Agaricomycetes*	*Russulales*	*Peniophoraceae*	*Peniophora*	*Peniophora versicolor*	0.0	0.0	0.0	0.0	0.0	0.0	0.0	0.6	0.0	0.0	0.0	0.0
OTU356	*Basidiomycota*	*Agaricomycetes*	*Russulales*	*Stereaceae*	*Stereum*	*Stereum hirsutum*	0.0	0.0	0.0	0.0	0.0	0.0	0.0	0.1	0.0	0.0	0.0	0.0
OTU357	*Basidiomycota*	*Agaricomycetes*	*Russulales*	*Stereaceae*	unassigned	unassigned	0.0	0.0	0.0	0.0	0.0	0.0	0.0	0.0	0.0	0.0	0.0	0.0
OTU358	*Basidiomycota*	*Agaricomycetes*	*Sebacinales*	*Sebacinaceae*	*Chaetospermum*	unidentified	0.0	0.0	0.0	0.0	0.0	0.0	0.0	0.0	0.0	0.0	0.0	0.0
OTU359	*Basidiomycota*	*Agaricomycetes*	*Sebacinales*	*Sebacinaceae*	*Sebacina*	*Sebacina epigaea*	0.0	0.0	0.0	0.0	0.0	0.0	0.0	0.0	0.0	0.0	0.0	0.0
OTU360	*Basidiomycota*	*Agaricomycetes*	*Sebacinales*	*Sebacinaceae*	*Sebacina*	unidentified	0.0	0.0	0.0	0.0	0.0	0.0	0.0	0.0	0.0	0.0	0.0	0.0

OTU361	*Basidiomycota*	*Agaricomycetes*	*Sebacinales*	*Serendipitaceae*	*Serendipita*	unidentified	0.0	0.0	0.0	0.0	0.0	0.0	0.0	0.3	0.0	0.0	0.0	0.0
OTU362	*Basidiomycota*	*Agaricomycetes*	*Sebacinales*	unassigned	unassigned	unassigned	0.0	0.0	0.0	0.0	0.0	0.0	0.0	0.0	0.0	0.0	0.0	0.0
OTU363	*Basidiomycota*	*Agaricomycetes*	*Sebacinales*	unidentified	unidentified	unidentified	0.0	0.0	0.0	0.0	0.0	0.0	0.0	0.0	0.0	0.0	0.0	0.0
OTU364	*Basidiomycota*	*Agaricomycetes*	*Thelephorales*	*Thelephoraceae*	*Pseudotomentella*	unidentified	0.0	0.0	0.0	0.0	0.0	0.0	0.0	0.0	0.0	0.0	0.0	0.0
OTU365	*Basidiomycota*	*Agaricomycetes*	*Thelephorales*	*Thelephoraceae*	*Tomentella*	*Tomentella badia*	0.0	0.0	0.0	0.0	0.0	0.0	0.1	0.0	0.0	0.0	0.0	0.0
OTU366	*Basidiomycota*	*Agaricomycetes*	*Thelephorales*	*Thelephoraceae*	*Tomentella*	*Tomentella galzinii*	0.0	0.0	0.0	0.0	0.0	0.0	0.0	0.1	0.0	0.0	0.0	0.0
OTU367	*Basidiomycota*	*Agaricomycetes*	*Thelephorales*	*Thelephoraceae*	*Tomentella*	unassigned	0.0	0.0	0.0	0.0	0.0	0.0	0.0	0.0	0.0	0.0	0.0	0.0
OTU368	*Basidiomycota*	*Agaricomycetes*	*Thelephorales*	*Thelephoraceae*	unassigned	unassigned	0.0	0.0	0.0	0.0	0.0	0.0	1.7	0.8	0.0	0.0	0.0	0.0
OTU369	*Basidiomycota*	*Agaricomycetes*	*Trechisporales*	*Hydnodontaceae*	*Brevicellicium*	*Brevicellicium olivascens*	0.0	0.0	0.0	0.0	0.0	0.0	0.0	0.0	0.0	0.0	0.0	0.0
OTU370	*Basidiomycota*	*Agaricomycetes*	*Trechisporales*	*Hydnodontaceae*	*Trechispora*	*Trechispora cohaerens*	0.0	0.0	0.0	0.0	0.0	0.0	0.0	0.0	0.0	0.0	0.0	0.0
OTU371	*Basidiomycota*	*Agaricomycetes*	*Trechisporales*	*Hydnodontaceae*	*Trechispora*	*Trechispora invisitata*	0.0	0.0	0.0	0.0	0.0	0.0	0.0	0.0	0.0	0.0	0.0	0.0
OTU372	*Basidiomycota*	*Agaricomycetes*	*Trechisporales*	*Hydnodontaceae*	*Trechispora*	unassigned	0.0	0.0	0.0	0.0	0.0	0.0	0.0	0.0	0.0	0.0	0.0	0.0
OTU373	*Basidiomycota*	*Agaricomycetes*	*Trechisporales*	*Hydnodontaceae*	*Trechispora*	unidentified	0.0	0.0	0.0	0.0	0.0	0.0	0.0	0.1	0.0	0.0	0.0	0.0
OTU374	*Basidiomycota*	*Agaricomycetes*	*Trechisporales*	*Trechisporales fam Incertae sedis*	*Sistotremastrum*	*Sistotremastrum guttuliferum*	0.0	0.0	0.0	0.0	0.0	0.0	0.0	0.1	0.0	0.0	0.0	0.0
OTU375	*Basidiomycota*	*Agaricomycetes*	unassigned	unassigned	unassigned	unassigned	0.0	0.0	0.0	0.0	0.0	0.0	9.2	0.2	0.0	0.0	0.0	0.0
OTU376	*Basidiomycota*	*Agaricomycetes*	unidentified	unidentified	unidentified	unidentified	5.5	0.0	0.0	0.0	0.0	0.0	0.0	0.3	2.1	0.0	0.0	0.0
OTU377	*Basidiomycota*	*Agaricostilbomycetes*	*Agaricostilbales*	*Chionosphaeraceae*	*Kurtzmanomyces*	*Kurtzmanomyces tardus*	0.0	0.0	0.0	0.0	0.0	0.0	0.0	0.0	0.2	0.0	0.0	0.0
OTU378	*Basidiomycota*	*Agaricostilbomycetes*	*Agaricostilbales*	*Kondoaceae*	*Bensingtonia*	unassigned	0.0	0.0	0.0	0.0	0.0	0.0	0.0	0.0	0.0	0.0	0.0	0.0
OTU379	*Basidiomycota*	*Atractiellomycetes*	*Atractiellales*	*Atractiellales fam Incertae sedis*	*Helicogloea*	*Helicogloea sebacea*	0.0	0.0	0.0	0.0	0.0	0.0	0.0	0.0	0.0	0.0	0.0	0.0
OTU380	*Basidiomycota*	*Cystobasidiomycetes*	*Cystobasidiomycetes ord Incertae sedis*	*Buckleyzymaceae*	*Buckleyzyma*	unidentified	0.0	0.0	0.0	0.0	0.0	50.0	0.0	0.0	0.0	0.0	0.0	0.0
OTU381	*Basidiomycota*	*Cystobasidiomycetes*	*Erythrobasidiales*	*Erythrobasidiales fam Incertae sedis*	*Sakaguchia*	unidentified	0.0	0.0	0.0	0.0	0.0	0.0	0.0	0.0	3.3	0.0	0.0	0.0
OTU382	*Basidiomycota*	*Exobasidiomycetes*	*Entylomatales*	unidentified	unidentified	unidentified	0.0	0.0	0.0	0.0	0.0	0.0	0.0	0.0	0.0	0.0	0.0	0.0
OTU383	*Basidiomycota*	*Exobasidiomycetes*	*Golubeviales*	*Golubeviaceae*	*Golubevia*	*Golubevia pallescens*	0.0	0.0	0.0	0.0	0.0	0.0	0.0	0.0	0.0	0.0	0.0	0.0
OTU384	*Basidiomycota*	*Geminibasidiomycetes*	*Geminibasidiales*	*Geminibasidiaceae*	*Basidioascus*	*Basidioascus persicus*	0.0	0.0	0.0	0.8	0.0	0.0	0.0	0.0	0.0	0.0	0.0	0.0
OTU385	*Basidiomycota*	*Geminibasidiomycetes*	*Geminibasidiales*	*Geminibasidiaceae*	*Geminibasidium*	unidentified	12.8	0.0	0.0	0.0	0.0	0.0	0.0	0.0	2.0	0.1	0.0	0.0
OTU386	*Basidiomycota*	*Geminibasidiomycetes*	*Geminibasidiales*	unassigned	unassigned	unassigned	0.0	0.0	0.0	0.0	0.0	0.0	0.0	0.0	0.0	0.0	0.0	0.0
OTU387	*Basidiomycota*	*Malasseziomycetes*	*Malasseziales*	*Malasseziaceae*	*Malassezia*	*Malassezia globosa*	0.9	0.0	0.0	0.0	20.6	0.0	0.0	0.0	0.0	0.0	0.0	0.0
OTU388	*Basidiomycota*	*Malasseziomycetes*	*Malasseziales*	*Malasseziaceae*	*Malassezia*	*Malassezia restricta*	3.2	0.0	0.0	0.0	0.0	0.0	0.0	0.0	0.0	0.0	0.0	0.0
OTU389	*Basidiomycota*	*Microbotryomycetes*	*Sporidiobolales*	*Sporidiobolaceae*	*Rhodosporidiobolus*	*Rhodosporidiobolus odoratus*	0.0	0.0	0.0	0.0	0.0	0.0	0.0	0.0	0.0	0.0	0.0	0.0
OTU390	*Basidiomycota*	*Microbotryomycetes*	*Sporidiobolales*	*Sporidiobolaceae*	*Rhodotorula*	*Rhodotorula mucilaginosa*	0.0	0.0	0.0	0.0	0.0	0.0	0.0	0.0	0.0	0.0	0.0	0.1
OTU391	*Basidiomycota*	*Microbotryomycetes*	*Sporidiobolales*	*Sporidiobolaceae*	*Sporobolomyces*	*Sporobolomyces roseus*	0.0	0.0	0.0	0.0	0.0	0.0	0.0	0.0	0.0	0.0	0.0	0.0
OTU392	*Basidiomycota*	*Microbotryomycetes*	*Sporidiobolales*	*Sporidiobolaceae*	*Sporobolomyces*	*Sporobolomyces ruberrimus*	0.0	0.0	0.0	0.0	0.0	0.0	0.0	0.0	0.0	0.0	0.0	0.0
OTU393	*Basidiomycota*	*Microbotryomycetes*	*Sporidiobolales*	unidentified	unidentified	unidentified	0.0	0.0	0.0	0.0	0.0	0.0	0.0	0.0	0.0	0.0	0.0	0.0

OTU394	*Basidiomycota*	*Microbotryomycetes*	unassigned	unassigned	unassigned	unassigned	0.0	0.0	0.0	0.0	0.0	0.0	0.0	0.0	0.0	0.0	0.0	0.0
OTU395	*Basidiomycota*	*Pucciniomycetes*	*Platygloeales*	unassigned	unassigned	unassigned	0.0	0.0	0.0	0.0	0.0	0.0	0.0	0.0	0.0	0.0	0.0	0.0
OTU396	*Basidiomycota*	*Tremellomycetes*	*Cystofilobasidiales*	*Mrakiaceae*	*Tausonia*	*Tausonia pullulans*	0.0	0.0	0.0	0.0	0.0	0.0	0.0	0.0	3.7	0.0	0.0	0.0
OTU397	*Basidiomycota*	*Tremellomycetes*	*Cystofilobasidiales*	*Mrakiaceae*	*Udeniomyces*	*Udeniomyces pyricola*	0.0	0.0	0.0	0.0	0.0	0.0	0.0	0.0	0.0	0.0	0.0	0.0
OTU398	*Basidiomycota*	*Tremellomycetes*	*Filobasidiales*	*Filobasidiaceae*	*Filobasidium*	*Filobasidium globisporum*	0.0	0.0	0.0	0.0	0.0	0.0	0.0	0.0	0.0	0.0	0.0	0.0
OTU399	*Basidiomycota*	*Tremellomycetes*	*Filobasidiales*	*Piskurozymaceae*	*Solicoccozyma*	*Solicoccozyma aeria*	0.0	0.0	0.0	0.0	0.0	0.0	0.0	0.0	0.0	0.0	0.0	0.0
OTU400	*Basidiomycota*	*Tremellomycetes*	*Tremellales*	*Bulleribasidiaceae*	*Vishniacozyma*	*Vishniacozyma victoriae*	0.0	0.0	0.0	0.0	0.0	0.0	0.0	0.0	0.0	0.0	0.0	0.0
OTU401	*Basidiomycota*	*Tremellomycetes*	*Tremellales*	*Cuniculitremaceae*	*Kockovaella*	*Kockovaella schimae*	0.0	0.0	0.0	0.0	0.0	0.0	0.0	0.0	0.0	0.0	0.0	0.0
OTU402	*Basidiomycota*	*Tremellomycetes*	*Tremellales*	*Cuniculitremaceae*	unassigned	unassigned	0.0	0.0	0.0	0.0	0.0	0.0	0.0	0.3	0.0	0.0	0.0	0.0
OTU403	*Basidiomycota*	*Tremellomycetes*	*Tremellales*	*Rhynchogastremataceae*	*Papiliotrema*	unassigned	0.0	0.0	0.0	0.0	0.0	0.0	0.0	0.0	0.0	0.0	0.0	0.0
OTU404	*Basidiomycota*	*Tremellomycetes*	*Tremellales*	unassigned	unassigned	unassigned	0.0	0.0	0.0	0.0	0.0	0.0	0.0	0.0	0.0	0.0	0.0	0.0
OTU405	*Basidiomycota*	*Tremellomycetes*	*Trichosporonales*	*Trichosporonaceae*	*Apiotrichum*	*Apiotrichum gracile*	0.0	0.0	0.0	0.0	0.0	0.0	0.0	0.3	0.0	0.0	0.0	0.0
OTU406	*Basidiomycota*	*Tremellomycetes*	*Trichosporonales*	*Trichosporonaceae*	*Apiotrichum*	*Apiotrichum laibachii*	0.0	0.0	0.0	0.0	0.0	0.0	0.0	17.8	0.0	0.0	16.7	0.0
OTU407	*Basidiomycota*	*Tremellomycetes*	*Trichosporonales*	*Trichosporonaceae*	*Apiotrichum*	unassigned	0.0	0.0	0.0	0.0	0.0	0.0	0.0	0.0	0.0	0.0	0.0	0.0
OTU408	*Basidiomycota*	*Tremellomycetes*	*Trichosporonales*	*Trichosporonaceae*	*Cutaneotrichosporon*	*Cutaneotrichosporon moniliiforme*	0.0	0.0	0.0	0.0	0.0	0.0	0.0	0.1	0.0	0.0	0.0	0.2
OTU409	*Basidiomycota*	*Tritirachiomycetes*	*Tritirachiales*	*Tritirachiaceae*	*Tritirachium*	*Tritirachium dependens*	4.2	0.0	0.0	0.0	0.0	0.0	0.0	0.0	0.0	0.0	0.0	0.0
OTU410	*Basidiomycota*	unassigned	unassigned	unassigned	unassigned	unassigned	0.0	0.0	0.0	0.0	0.0	0.0	0.1	0.0	0.0	0.0	0.0	0.0
OTU411	*Chytridiomycota*	*Chytridiomycetes*	*Chytridiales*	*Chytridiaceae*	*Phlyctochytrium*	*Phlyctochytrium africanum*	0.0	0.0	0.0	0.0	0.0	0.0	0.0	0.4	0.0	0.0	0.0	0.0
OTU412	*Chytridiomycota*	*unidentified*	*unidentified*	*unidentified*	unidentified	unidentified	0.0	0.0	0.0	0.0	0.0	0.0	0.0	0.0	0.0	0.0	0.0	0.0
OTU413	*Glomeromycota*	*Glomeromycetes*	*Diversisporales*	*Diversisporaceae*	unassigned	unassigned	0.0	0.0	0.0	0.0	0.0	0.0	0.0	0.0	0.0	0.0	0.0	0.0
OTU414	*Glomeromycota*	*Glomeromycetes*	*Glomerales*	*Glomeraceae*	*Glomus*	unidentified	0.0	0.0	0.0	0.0	0.0	0.0	0.0	0.0	0.0	0.0	0.0	0.0
OTU415	*Glomeromycota*	*Glomeromycetes*	*Glomerales*	*Glomeraceae*	unassigned	unassigned	0.0	0.0	0.0	0.0	0.0	0.0	0.1	0.0	0.0	0.0	0.0	0.0
OTU416	*Glomeromycota*	*Glomeromycetes*	*Glomerales*	*Glomeraceae*	unidentified	unidentified	0.0	0.0	0.0	0.0	0.0	0.0	0.1	0.0	0.0	0.0	0.0	0.0
OTU417	*Kickxellomycota*	GS19	GS19	unidentified	unidentified	unidentified	0.0	0.0	0.0	0.0	0.0	0.0	0.0	0.1	0.0	0.0	0.0	0.0
OTU418	*Kickxellomycota*	*Kickxellomycetes*	*Kickxellales*	*Kickxellaceae*	*Ramicandelaber*	unidentified	0.0	0.0	0.0	0.0	0.0	0.0	0.0	0.0	0.0	0.9	0.0	0.0
OTU419	*Mortierellomycota*	*Mortierellomycetes*	*Mortierellales*	*Mortierellaceae*	*Mortierella*	*Mortierella alpina*	0.0	0.0	0.0	16.2	0.0	0.0	0.0	0.2	0.0	5.3	0.0	0.0
OTU420	*Mortierellomycota*	*Mortierellomycetes*	*Mortierellales*	*Mortierellaceae*	*Mortierella*	*Mortierella beljakovae*	0.0	0.0	0.0	0.0	0.0	0.0	0.0	0.0	0.0	0.0	0.0	0.0
OTU421	*Mortierellomycota*	*Mortierellomycetes*	*Mortierellales*	*Mortierellaceae*	*Mortierella*	*Mortierella chienii*	0.0	0.0	0.0	0.0	0.0	0.0	0.0	0.0	0.0	0.2	0.0	0.0
OTU422	*Mortierellomycota*	*Mortierellomycetes*	*Mortierellales*	*Mortierellaceae*	*Mortierella*	*Mortierella elongata*	0.0	0.0	0.0	0.0	0.0	0.0	0.0	0.0	0.0	0.1	0.0	0.0
OTU423	*Mortierellomycota*	*Mortierellomycetes*	*Mortierellales*	*Mortierellaceae*	*Mortierella*	*Mortierella gamsii*	0.0	0.0	0.0	0.0	0.0	0.0	0.0	0.0	0.0	0.0	0.0	0.0
OTU424	*Mortierellomycota*	*Mortierellomycetes*	*Mortierellales*	*Mortierellaceae*	*Mortierella*	*Mortierella humilis*	0.0	0.0	0.0	0.0	0.0	0.0	0.0	0.0	0.0	0.0	0.0	0.4
OTU425	*Mortierellomycota*	*Mortierellomycetes*	*Mortierellales*	*Mortierellaceae*	*Mortierella*	*Mortierella minutissima*	0.0	0.0	0.0	0.0	0.0	0.0	0.0	0.0	0.0	0.0	0.6	0.6
OTU426	*Mortierellomycota*	*Mortierellomycetes*	*Mortierellales*	*Mortierellaceae*	*Mortierella*	*Mortierella selenospora*	0.0	0.0	0.0	0.0	0.0	0.0	0.0	0.0	0.0	0.4	0.0	0.0

OTU427	*Mortierellomycota*	*Mortierellomycetes*	*Mortierellales*	*Mortierellaceae*	*Mortierella*	unassigned	0.0	0.0	0.0	0.0	0.0	0.0	0.0	1.5	0.2	0.0	0.0	0.0
OTU428	*Mortierellomycota*	*Mortierellomycetes*	*Mortierellales*	*Mortierellaceae*	*Mortierella*	unidentified	0.0	0.0	0.0	5.1	0.0	0.0	0.0	0.1	0.3	0.0	0.0	0.0
OTU429	*Mucoromycota*	*Mucoromycetes*	*Mucorales*	*Chaetocladiaceae*	*Chaetocladium*	*Chaetocladium brefeldii*	0.0	0.0	0.0	0.0	0.0	0.0	0.0	0.0	0.0	0.0	0.0	0.0
OTU430	*Mucoromycota*	*Mucoromycetes*	*Mucorales*	*Mucoraceae*	*Mucor*	*Mucor circinelloides*	0.0	0.0	0.0	0.0	0.0	0.0	0.0	0.4	0.0	0.1	0.8	0.0
OTU431	*Mucoromycota*	*Mucoromycetes*	*Mucorales*	*Mucoraceae*	*Mucor*	*Mucor fragilis*	0.0	0.0	0.0	0.0	0.0	0.0	0.0	0.0	0.0	0.0	0.0	0.0
OTU432	*Mucoromycota*	*Mucoromycetes*	*Mucorales*	*Mucoraceae*	*Mucor*	*Mucor plasmaticus*	0.0	0.0	0.0	0.0	0.0	0.0	0.0	0.0	0.0	0.0	0.0	0.0
OTU433	*Mucoromycota*	*Mucoromycetes*	*Mucorales*	*Mucoraceae*	*Mucor*	*Mucor racemosus*	0.0	0.0	0.0	0.0	0.0	0.0	0.0	0.0	0.0	0.0	1.2	0.0
OTU434	*Mucoromycota*	*Mucoromycetes*	*Mucorales*	*Mucoraceae*	*Parasitella*	*Parasitella parasitica*	0.0	0.0	0.0	0.0	0.0	0.0	0.0	0.0	0.0	0.0	0.0	0.0
OTU435	*Mucoromycota*	*Mucoromycotina cls Incertae sedis*	*Mucoromycotina ord Incertae sedis*	*Mucoromycotina fam Incertae sedis*	*Bifiguratus*	unidentified	0.0	0.0	0.0	0.0	0.0	0.0	0.0	0.0	0.0	0.0	0.0	0.0
OTU436	unassigned	unassigned	unassigned	unassigned	unassigned	unassigned	0.0	0.0	0.0	0.0	0.0	0.0	0.2	2.7	14.8	0.2	1.2	0.0
OTU437	*Zoopagomycota*	*Zoopagomycetes*	*Zoopagales*	*Piptocephalidaceae*	*Syncephalis*	unidentified	0.0	0.0	0.0	0.0	0.0	0.0	0.0	0.0	0.0	0.0	0.0	0.0

Table S5. Fungal annotation based on FUNGuild database of isolates obtained by culture techniques from the sediments of Castañar Cave in 2008, 2009 and 2021.

Species	Number of isolates				FUNGuild taxon	trophicMode	guild
	Jurado et al. 2010 Year of sampling 2008	Jurado et al. 2010 Year of sampling 2009	This study Year of sampling 2009	This study Year of sampling 2021			
Alternaria citrimacularis	0	0	0	1	*Pleosporaceae*	Pathotroph-Saprotroph	Endophyte-Lichen Parasite-Plant Pathogen-Undefined Saprotroph
Cladosporium cladosporioides	0	0	3	5	*Cladosporium cladosporioides*	Pathotroph-Symbiotroph	Endophyte-Epiphyte-Plant Pathogen
Cladosporium delicatulum	0	0	0	2	unassigned	unassigned	unassigned
Cladosporium herbarum	0	0	1	0	*Cladosporium herbarum*	Pathotroph-Saprotroph	Plant Pathogen-Wood Saprotroph
Cladosporium sphaerospermum	0	0	1	0	*Cladosporium sphaerospermum*	Symbiotroph	Endophyte
Cladosporium sp.	0	1	0	0	unassigned	unassigned	unassigned
Epicoccum nigrum	0	0	0	2	*Didymellaceae*	Pathotroph-Saprotroph	Animal Pathogen-Plant Pathogen-Undefined Saprotroph
Phoma sp.	0	0	1	0	*Didymellaceae*	Pathotroph-Saprotroph	Animal Pathogen-Plant Pathogen-Undefined Saprotroph
Arthroderma terrestre	0	0	1	0	*Arthrodermataceae*	Pathotroph-Saprotroph	Animal Pathogen-Undefined Saprotroph
Aspergillus allahabadii	0	0	0	1	unassigned	unassigned	unassigned
Aspergillus aureolatus	0	0	1	0	unassigned	unassigned	unassigned
Aspergillus calidoustus	0	0	2	0	*Aspergillus calidoustus*	Pathotroph	Animal Pathogen
Aspergillus flavus	0	0	0	2	*unassigned*	unassigned	unassigned
Aspergillus fumigatus	0	0	2	0	*Aspergillus fumigatus*	Pathotroph-Saprotroph	Animal Pathogen-Undefined Saprotroph
Aspergillus sp.	0	0	1	0	unassigned	unassigned	unassigned
Aspergillus spelunceus	0	0	2	0	unassigned	unassigned	unassigned
Aspergillus versicolor	0	0	0	1	*Sterigmatocystis*	Saprotroph	Undefined Saprotroph
Aspergillus ustus	2	1	0	0	*Aspergillus ustus*	Saprotroph	Wood Saprotroph
Cladophialophora sp.	0	0	1	0	*Herpotrichiellaceae*	Pathotroph-Saprotroph	Animal Pathogen-Fungal Parasite-Undefined Saprotroph
Penicillium sp.	2	0	0	6	*Penicillium*	Saprotroph	Dung Saprotroph-Undefined Saprotroph-Wood Saprotroph
Penicillium brevicompactum	0	0	0	2	*Penicillium brevicompactum*	Symbiotroph	Endophyte
Penicillium chrysogenum	0	0	0	5	*Penicillium chrysogenum*	Pathotroph	Plant Pathogen
Penicillium cyclopium	0	0	1	0	*Penicillium cyclopium*	Pathotroph	Plant Pathogen
Penicillium decumbens	0	0	3	0	*Penicillium decumbens*	Pathotroph	Animal Pathogen
Penicillium expansum	0	0	1	0	*Penicillium expansum*	Pathotroph	Plant Pathogen
Penicillium glabrum	0	0	1	0	*Penicillium glabrum*	Pathotroph	Plant Pathogen
Penicillium goetzii	0	0	0	5	*Penicillium*	Saprotroph	Dung Saprotroph-Undefined Saprotroph-Wood Saprotroph
Penicillium laevigatum	0	0	0	4	*Penicillium*	Saprotroph	Dung Saprotroph-Undefined Saprotroph-Wood Saprotroph
Penicillium rubens	0	0	0	1	*Penicillium*	Saprotroph	Dung Saprotroph-Undefined Saprotroph-Wood Saprotroph
Penicillium sumatrense	0	0	0	1	*Penicillium*	Saprotroph	Dung Saprotroph-Undefined Saprotroph-Wood Saprotroph
Talaromyces rogersiae	0	0	0	1	*Talaromyces*	Saprotroph	Undefined Saprotroph
Trichophyton sp.	0	0	2	0	*Arthrodermataceae*	Pathotroph-Saprotroph	Animal Pathogen-Undefined Saprotroph

Hormiactis fimicola	0	0	1	0	*Hormiactis*	Pathotroph	Fungal Parasite
Albifimbria verrucaria	0	0	0	1	*Albifimbria verrucaria*	Pathotroph-Saprotroph	Fungal Parasite-Soil Saprotroph-Undefined Saprotroph
Amphichorda felina	0	0	2	0	unassigned	unassigned	unassigned
Aphanocladium album	0	0	4	0	*Nectriaceae*	Pathotroph-Saprotroph-Symbiotroph	Animal Pathogen-Endophyte-Fungal Parasite-Lichen Parasite-Plant Pathogen-Wood Saprotroph
Botryotrichum piluliferum	0	0	2	0	*Botryotrichum piluliferum*	Saprotroph	Wood Saprotroph
Cadophora fastigiata	0	0	1	0	*Cadophora*	Symbiotroph	Endophyte
Cephalotrichum asperulum	0	0	1	0	*Microascaceae*	Pathotroph-Saprotroph-Symbiotroph	Animal Pathogen-Endophyte-Plant Pathogen-Undefined Saprotroph
Cephalotrichum microsporum	0	0	4	6	*Microascaceae*	Pathotroph-Saprotroph-Symbiotroph	Animal Pathogen-Endophyte-Plant Pathogen-Undefined Saprotroph
Cephalotrichum nanum	0	0	0	3	*Microascaceae*	Pathotroph-Saprotroph-Symbiotroph	Animal Pathogen-Endophyte-Plant Pathogen-Undefined Saprotroph
Cephalotrichum sp	0	3	0	0	*Microascaceae*	Pathotroph-Saprotroph-Symbiotroph	Animal Pathogen-Endophyte-Plant Pathogen-Undefined Saprotroph
Cephalotrichum stemonitis	0	0	4	0	*Microascaceae*	Pathotroph-Saprotroph-Symbiotroph	Animal Pathogen-Endophyte-Plant Pathogen-Undefined Saprotroph
Chaetomium globosum	1	1	0	0	*Chaetomium globosum*	Pathotroph-Saprotroph-Symbiotroph	Dung Saprotroph-Endophyte-Epiphyte-Wood Saprotroph
Chaetomium sp.	0	0	1	0	unassigned	unassigned	unassigned
Chrysosporium sp.	0	0	2	0	unassigned	unassigned	unassigned
Clonostachys rosea	0	0	0	1	*Bionectriaceae*	Pathotroph-Saprotroph	Algal Parasite-Bryophyte Parasite-Fungal Parasite-Undefined Saprotroph
Cylindrocarpon sp.	0	0	2	0	*Nectriaceae*	Pathotroph-Saprotroph-Symbiotroph	Animal Pathogen-Endophyte-Fungal Parasite-Lichen Parasite-Plant Pathogen-Wood Saprotroph
Fusarium brachygibbosum	0	0	0	1	*Nectriaceae*	Pathotroph-Saprotroph-Symbiotroph	Animal Pathogen-Endophyte-Fungal Parasite-Lichen Parasite-Plant Pathogen-Wood Saprotroph
Fusarium oxysporum	2	0	0	4	*Nectriaceae*	Pathotroph-Saprotroph-Symbiotroph	Animal Pathogen-Endophyte-Fungal Parasite-Lichen Parasite-Plant Pathogen-Wood Saprotroph
Fusarium sp.	0	4	0	1	*Nectriaceae*	Pathotroph-Saprotroph-Symbiotroph	Animal Pathogen-Endophyte-Fungal Parasite-Lichen Parasite-Plant Pathogen-Wood Saprotroph
Fusarium tricinctum	1	0	0	0	*Nectriaceae*	Pathotroph-Saprotroph-Symbiotroph	Animal Pathogen-Endophyte-Fungal Parasite-Lichen Parasite-Plant Pathogen-Wood Saprotroph
Fusarium avenaceum	0	3	0	0	*Nectriaceae*	Pathotroph-Saprotroph-Symbiotroph	Animal Pathogen-Endophyte-Fungal Parasite-Lichen Parasite-Plant Pathogen-Wood Saprotroph
Fusarium verticillioides	0	0	0	3	*Nectriaceae*	Pathotroph-Saprotroph-Symbiotroph	Animal Pathogen-Endophyte-Fungal Parasite-Lichen Parasite-Plant Pathogen-Wood Saprotroph
Humicola sp.	0	0	2	0	*unassigned*	unassigned	unassigned
Metacordyceps chlamydosporia	0	4	0	0	*Clavicipitaceae*	Pathotroph-Symbiotroph	Endophyte-Epiphyte-Fungal Parasite-Insect Parasite
Neocosmospora solani	4	2	2	6	*Nectriaceae*	Pathotroph-Saprotroph-Symbiotroph	Animal Pathogen-Endophyte-Fungal Parasite-Lichen Parasite-Plant Pathogen-Wood Saprotroph
Volutella consors	1	0	0	0	*Nectriaceae*	Pathotroph-Saprotroph-Symbiotroph	Animal Pathogen-Endophyte-Fungal Parasite-Lichen Parasite-Plant Pathogen-Wood Saprotroph
Purpureocillium lilacinum	1	0	2	0	*Purpureocillium*	Pathotroph	Fungal Parasite
Staphylotrichum coccosporum	0	0	2	0	*Staphylotrichum*	Saprotroph	Undefined Saprotroph
Trichocladium uniseriatum	0	0	0	1	*Trichocladium*	Saprotroph	Undefined Saprotroph
Trichoderma harzianum	0	0	0	1	*Trichoderma harzianum*	Pathotroph	Endophyte-Fungal Parasite-Plant Pathogen
Trichoderma virens	0	0	0	1	*Trichoderma virens*	Symbiotroph	Endophyte
Trichoderma lixii	1	0	0	0	*Trichoderma*	Pathotroph-Saprotroph-Symbiotroph	Animal Pathogen-Endophyte-Epiphyte-Fungal Parasite-Plant Pathogen-Wood Saprotroph
Wardomyces inflatus	0	0	2	0	*Microascaceae*	Pathotroph-Saprotroph-Symbiotroph	Animal Pathogen-Endophyte-Plant Pathogen-Undefined Saprotroph
Heterobasidion parviporum	0	0	1	0	*Bondarzewiaceae*	Pathotroph-Saprotroph	Plant Pathogen-Wood Saprotroph
Linnemannia elongata	0	0	0	5	*Mortierellaceae*	Saprotroph-Symbiotroph	Endophyte-Litter Saprotroph-Soil Saprotroph-Undefined Saprotroph

Mortierella alpina	1	0	0	1	*Mortierellaceae*	Saprotroph-Symbiotroph	Endophyte-Litter Saprotroph-Soil Saprotroph-Undefined Saprotroph
Mortierella sp.	0	0	0	2	*Mortierellaceae*	Saprotroph-Symbiotroph	Endophyte-Litter Saprotroph-Soil Saprotroph-Undefined Saprotroph
Absidia repens	1	0	0	0	*Absidia*	Saprotroph	Undefined Saprotroph
Mucor plumbeus	0	0	0	2	unassigned	unassigned	unassigned
Mucor hiemalis	0	0	1	1	*Mucor hiemalis*	Pathotroph	Plant Pathogen
Mucor racemosus	0	1	0	4	*Mucor racemosus*	Pathotroph-Symbiotroph	Endophyte-Plant Pathogen
Mucor circinelloides	11	1	0	0	*Mucor circinelloides*	Pathotroph	Animal Pathogen-Plant Pathogen

Table S6. Fungal annotation based on FUNGuild database of isolates obtained by NGS techniques from the sediments of Castañar Cave in 2020 and 2021.

**OTU**	**FUNGuild taxon**	**trophicMode**	**guild**	**growthForm**
OTU1	unassigned	unassigned	unassigned	unassigned
OTU2	unassigned	unassigned	unassigned	unassigned
OTU3	*Cladosporium cladosporioides*	Pathotroph-Symbiotroph	Endophyte-Epiphyte-Plant Pathogen	Microfungus
OTU4	*Cladosporium sphaerospermum*	Symbiotroph	Endophyte	Microfungus
OTU5	*Mycosphaerellaceae*	Pathotroph-Saprotroph	Plant Pathogen-Undefined Saprotroph	Microfungus
OTU6	*Aureobasidium pullulans*	Pathotroph-Symbiotroph	Animal Pathogen-Endophyte-Epiphyte-Plant Pathogen	unassigned
OTU7	*Kabatiella*	Pathotroph-Saprotroph-Symbiotroph	Endophyte-Plant Pathogen-Undefined Saprotroph	Microfungus
OTU8	*Hormonema*	Saprotroph	Undefined Saprotroph	Facultative Yeast
OTU9	unassigned	unassigned	unassigned	unassigned
OTU10	unassigned	unassigned	unassigned	unassigned
OTU11	*Cucurbitariaceae*	Pathotroph-Saprotroph	Plant Pathogen-Wood Saprotroph	Microfungus
OTU12	*Didymellaceae*	Pathotroph-Saprotroph	Animal Pathogen-Plant Pathogen-Undefined Saprotroph	Microfungus
OTU13	*Didymellaceae*	Pathotroph-Saprotroph	Animal Pathogen-Plant Pathogen-Undefined Saprotroph	Microfungus
OTU14	*Didymosphaeriaceae*	Pathotroph-Saprotroph-Symbiotroph	Endophyte-Lichen Parasite-Plant Pathogen-Undefined Saprotroph	Microfungus
OTU15	*Didymosphaeriaceae*	Pathotroph-Saprotroph-Symbiotroph	Endophyte-Lichen Parasite-Plant Pathogen-Undefined Saprotroph	Microfungus
OTU16	*Didymosphaeriaceae*	Pathotroph-Saprotroph-Symbiotroph	Endophyte-Lichen Parasite-Plant Pathogen-Undefined Saprotroph	Microfungus
OTU17	*Massariunassignedceae*	Pathotroph-Saprotroph	Plant Pathogen-Undefined Saprotroph	Microfungus
OTU18	*Acrocalymma*	Saprotroph	Undefined Saprotroph	unassigned
OTU19	*Phaeosphaeriaceae*	Pathotroph-Saprotroph	Fungal Parasite-Plant Pathogen-Plant Saprotroph	Microfungus
OTU20	*Pleosporaceae*	Pathotroph-Saprotroph	Endophyte-Lichen Parasite-Plant Pathogen-Undefined Saprotroph	Microfungus
OTU21	*Pleosporaceae*	Pathotroph-Saprotroph	Endophyte-Lichen Parasite-Plant Pathogen-Undefined Saprotroph	Microfungus
OTU22	*Pleosporaceae*	Pathotroph-Saprotroph	Endophyte-Lichen Parasite-Plant Pathogen-Undefined Saprotroph	Microfungus
OTU23	*Pleosporaceae*	Pathotroph-Saprotroph	Endophyte-Lichen Parasite-Plant Pathogen-Undefined Saprotroph	Microfungus
OTU24	*Pleosporaceae*	Pathotroph-Saprotroph	Endophyte-Lichen Parasite-Plant Pathogen-Undefined Saprotroph	Microfungus
OTU25	*Sporormiaceae*	Saprotroph	Dung Saprotroph-Plant Saprotroph	Microfungus
OTU26	*Sporormiaceae*	Saprotroph	Dung Saprotroph-Plant Saprotroph	Microfungus
OTU27	*Teichosporaceae*	Saprotroph	Undefined Saprotroph	Microfungus
OTU28	unassigned	unassigned	unassigned	unassigned
OTU29	unassigned	unassigned	unassigned	unassigned
OTU30	unassigned	unassigned	unassigned	unassigned
OTU31	*Ochroconis*	Saprotroph	Undefined Saprotroph	unassigned
OTU32	*Ochroconis tshawytschae*	Pathotroph	Animal Pathogen	unassigned
OTU33	*Cyphellophoraceae*	Pathotroph-Saprotroph	Animal Pathogen-Undefined Saprotroph	Facultative Yeast-Microfungus
OTU34	*Cyphellophoraceae*	Pathotroph-Saprotroph	Animal Pathogen-Undefined Saprotroph	Facultative Yeast-Microfungus
OTU35	*Cyphellophoraceae*	Pathotroph-Saprotroph	Animal Pathogen-Undefined Saprotroph	Facultative Yeast-Microfungus
OTU36	*Herpotrichiellaceae*	Pathotroph-Saprotroph	Animal Pathogen-Fungal Parasite-Undefined Saprotroph	Facultative Yeast-Microfungus
OTU37	*Herpotrichiellaceae*	Pathotroph-Saprotroph	Animal Pathogen-Fungal Parasite-Undefined Saprotroph	Facultative Yeast-Microfungus

OTU38	*Herpotrichiellaceae*	Pathotroph-Saprotroph	Animal Pathogen-Fungal Parasite-Undefined Saprotroph	Facultative Yeast-Microfungus
OTU39	*Herpotrichiellaceae*	Pathotroph-Saprotroph	Animal Pathogen-Fungal Parasite-Undefined Saprotroph	Facultative Yeast-Microfungus
OTU40	*Herpotrichiellaceae*	Pathotroph-Saprotroph	Animal Pathogen-Fungal Parasite-Undefined Saprotroph	Facultative Yeast-Microfungus
OTU41	*Herpotrichiellaceae*	Pathotroph-Saprotroph	Animal Pathogen-Fungal Parasite-Undefined Saprotroph	Facultative Yeast-Microfungus
OTU42	*Herpotrichiellaceae*	Pathotroph-Saprotroph	Animal Pathogen-Fungal Parasite-Undefined Saprotroph	Facultative Yeast-Microfungus
OTU43	unassigned	unassigned	unassigned	unassigned
OTU44	unassigned	unassigned	unassigned	unassigned
OTU45	*Aspergillus protuberus*	Pathotroph	Animal Pathogen	Microfungus
OTU46	unassigned	unassigned	unassigned	unassigned
OTU47	unassigned	unassigned	unassigned	unassigned
OTU48	unassigned	unassigned	unassigned	unassigned
OTU49	unassigned	unassigned	unassigned	unassigned
OTU50	*Penicillium brasilianum*	Pathotroph	Animal Pathogen	Microfungus
OTU51	*Penicillium brevicompactum*	Symbiotroph	Endophyte	Microfungus
OTU52	*Penicillium*	Saprotroph	Dung Saprotroph-Undefined Saprotroph-Wood Saprotroph	Microfungus
OTU53	*Penicillium*	Saprotroph	Dung Saprotroph-Undefined Saprotroph-Wood Saprotroph	Microfungus
OTU54	*Penicillium expansum*	Pathotroph	Plant Pathogen	Microfungus
OTU55	*Penicillium*	Saprotroph	Dung Saprotroph-Undefined Saprotroph-Wood Saprotroph	Microfungus
OTU56	*Penicillium*	Saprotroph	Dung Saprotroph-Undefined Saprotroph-Wood Saprotroph	Microfungus
OTU57	unassigned	unassigned	unassigned	unassigned
OTU58	*Talaromyces*	Saprotroph	Undefined Saprotroph	unassigned
OTU59	*Talaromyces diversus*	Pathotroph	Animal Pathogen	unassigned
OTU60	*Talaromyces*	Saprotroph	Undefined Saprotroph	unassigned
OTU61	*Talaromyces rugulosus*	Pathotroph	Animal Pathogen	unassigned
OTU62	*Talaromyces*	Saprotroph	Undefined Saprotroph	unassigned
OTU63	*Talaromyces*	Saprotroph	Undefined Saprotroph	unassigned
OTU64	unassigned	unassigned	unassigned	unassigned
OTU65	*Ajellomycetaceae*	Pathotroph-Saprotroph	Animal Pathogen-Undefined Saprotroph	unassigned
OTU66	*Arthrodermataceae*	Pathotroph-Saprotroph	Animal Pathogen-Undefined Saprotroph	unassigned
OTU67	*Arthrodermataceae*	Pathotroph-Saprotroph	Animal Pathogen-Undefined Saprotroph	unassigned
OTU68	*Arthrodermataceae*	Pathotroph-Saprotroph	Animal Pathogen-Undefined Saprotroph	unassigned
OTU69	*Gymnoascaceae*	Saprotroph	Dung Saprotroph-Plant Saprotroph	Microfungus
OTU70	*Gymnoascaceae*	Saprotroph	Dung Saprotroph-Plant Saprotroph	Microfungus
OTU71	*Gymnoascaceae*	Saprotroph	Dung Saprotroph-Plant Saprotroph	Microfungus
OTU72	*Gymnoascaceae*	Saprotroph	Dung Saprotroph-Plant Saprotroph	Microfungus
OTU73	*Gymnoascaceae*	Saprotroph	Dung Saprotroph-Plant Saprotroph	Microfungus
OTU74	*Onygeunassignedceae*	Saprotroph	Dung Saprotroph-Soil Saprotroph	Microfungus
OTU75	*Onygeunassignedceae*	Saprotroph	Dung Saprotroph-Soil Saprotroph	Microfungus
OTU76	*Onygeunassignedceae*	Saprotroph	Dung Saprotroph-Soil Saprotroph	Microfungus
OTU77	*Onygeunassignedceae*	Saprotroph	Dung Saprotroph-Soil Saprotroph	Microfungus
OTU78	*Onygeunassignedceae*	Saprotroph	Dung Saprotroph-Soil Saprotroph	Microfungus
OTU79	*Onygeunassignedceae*	Saprotroph	Dung Saprotroph-Soil Saprotroph	Microfungus
OTU80	*Arthropsis hispanica*	Pathotroph	Animal Pathogen	unassigned
OTU81	unassigned	unassigned	unassigned	unassigned
OTU82	unassigned	unassigned	unassigned	unassigned
OTU83	unassigned	unassigned	unassigned	unassigned
OTU84	*Malbranchea*	Saprotroph	Undefined Saprotroph	unassigned
OTU85	*Malbranchea*	Saprotroph	Undefined Saprotroph	unassigned
OTU86	unassigned	unassigned	unassigned	unassigned
OTU87	unassigned	unassigned	unassigned	unassigned
OTU88	unassigned	unassigned	unassigned	unassigned

OTU89	unassigned	unassigned	unassigned	unassigned
OTU90	*Geoglossaceae*	Saprotroph	Undefined Saprotroph	unassigned
OTU91	*Geoglossaceae*	Saprotroph	Undefined Saprotroph	unassigned
OTU92	unassigned	unassigned	unassigned	unassigned
OTU93	*Erysiphaceae*	Pathotroph	Plant Pathogen	Microfungus
OTU94	*Helotiaceae*	Saprotroph-Symbiotroph	Ectomycorrhizal-Fungal Parasite-Plant Pathogen-Wood Saprotroph	Microfungus
OTU95	*Helotiaceae*	Saprotroph-Symbiotroph	Ectomycorrhizal-Fungal Parasite-Plant Pathogen-Wood Saprotroph	Microfungus
OTU96	*Helotiaceae*	Saprotroph-Symbiotroph	Ectomycorrhizal-Fungal Parasite-Plant Pathogen-Wood Saprotroph	Microfungus
OTU97	*Helotiaceae*	Saprotroph-Symbiotroph	Ectomycorrhizal-Fungal Parasite-Plant Pathogen-Wood Saprotroph	Microfungus
OTU98	unassigned	unassigned	unassigned	unassigned
OTU99	*Rhexocercosporidium*	Pathotroph	Plant Pathogen	unassigned
OTU100	*Hyaloscyphaceae*	Saprotroph	Plant Saprotroph-Wood Saprotroph	Microfungus
OTU101	*Myxotrichaceae*	Saprotroph-Symbiotroph	Ectomycorrhizal-Undefined Saprotroph	Microfungus
OTU102	*Myxotrichaceae*	Saprotroph-Symbiotroph	Ectomycorrhizal-Undefined Saprotroph	Microfungus
OTU103	*Myxotrichaceae*	Saprotroph-Symbiotroph	Ectomycorrhizal-Undefined Saprotroph	Microfungus
OTU104	unassigned	unassigned	unassigned	unassigned
OTU105	unassigned	unassigned	unassigned	unassigned
OTU106	*Phacidium*	Pathotroph	Plant Pathogen	unassigned
OTU107	*Rhytismataceae*	Pathotroph-Saprotroph-Symbiotroph	Endophyte-Leaf Saprotroph-Plant Pathogen	Microfungus
OTU108	*Pseudeurotiaceae*	Saprotroph	Plant Saprotroph-Wood Saprotroph	Microfungus
OTU109	*Pseudeurotiaceae*	Saprotroph	Plant Saprotroph-Wood Saprotroph	Microfungus
OTU110	*Pseudeurotiaceae*	Saprotroph	Plant Saprotroph-Wood Saprotroph	Microfungus
OTU111	*Pseudeurotiaceae*	Saprotroph	Plant Saprotroph-Wood Saprotroph	Microfungus
OTU112	*Pseudeurotiaceae*	Saprotroph	Plant Saprotroph-Wood Saprotroph	Microfungus
OTU113	*Pseudeurotiaceae*	Saprotroph	Plant Saprotroph-Wood Saprotroph	Microfungus
OTU114	unassigned	unassigned	unassigned	unassigned
OTU115	*Orbiliaceae*	Saprotroph	Wood Saprotroph	Microfungus
OTU116	*Balsamia*	Symbiotroph	Ectomycorrhizal	Gasteroid
OTU117	*Pezizaceae*	Saprotroph-Symbiotroph	Dung Saprotroph-Ectomycorrhizal-Litter Saprotroph-Undefined Saprotroph	Pezizoid
OTU118	*Pyronemataceae*	Saprotroph-Symbiotroph	Dung Saprotroph-Ectomycorrhizal-Soil Saprotroph-Wood Saprotroph	Gasteroid-Pezizoid
OTU119	*Pyronemataceae*	Saprotroph-Symbiotroph	Dung Saprotroph-Ectomycorrhizal-Soil Saprotroph-Wood Saprotroph	Gasteroid-Pezizoid
OTU120	*Tuberaceae*	Symbiotroph	Ectomycorrhizal	Gasteroid
OTU121	*Tuberaceae*	Symbiotroph	Ectomycorrhizal	Gasteroid
OTU122	*Meyerozyma guilliermondii*	Pathotroph	Animal Pathogen	unassigned
OTU123	*Barnettozyma*	Saprotroph	Undefined Saprotroph	Yeast
OTU124	*Candida*	Pathotroph-Saprotroph-Symbiotroph	Animal Pathogen-Endophyte-Endosymbiont-Epiphyte-Soil Saprotroph-Undefined Saprotroph	Dimorphic Yeast
OTU125	*Candida parapsilosis*	Pathotroph	Animal Pathogen	Dimorphic Yeast
OTU126	unassigned	unassigned	unassigned	unassigned
OTU127	*Diaporthaceae*	Pathotroph-Saprotroph-Symbiotroph	Endophyte-Plant Pathogen-Plant Saprotroph	Microfungus
OTU128	*Plectosphaerellaceae*	Pathotroph-Saprotroph-Symbiotroph	Animal Pathogen-Endophyte-Fungal Parasite-Plant Pathogen-Wood Saprotroph	Microfungus
OTU129	*Musicillium*	Pathotroph	Plant Pathogen	unassigned
OTU130	*Plectosphaerella cucumeriunassigned*	Pathotroph-Symbiotroph	Endophyte-Plant Pathogen	Microfungus
OTU131	*Plectosphaerella*	Pathotroph-Symbiotroph	Endophyte-Plant Pathogen	Microfungus
OTU132	*Plectosphaerellaceae*	Pathotroph-Saprotroph-Symbiotroph	Animal Pathogen-Endophyte-Fungal Parasite-Plant Pathogen-Wood Saprotroph	Microfungus
OTU133	*Plectosphaerellaceae*	Pathotroph-Saprotroph-Symbiotroph	Animal Pathogen-Endophyte-Fungal Parasite-Plant Pathogen-Wood Saprotroph	Microfungus
OTU134	*Bionectriaceae*	Pathotroph-Saprotroph	Algal Parasite-Bryophyte Parasite-Fungal Parasite-Undefined Saprotroph	Microfungus
OTU135	*Bionectriaceae*	Pathotroph-Saprotroph	Algal Parasite-Bryophyte Parasite-Fungal Parasite-Undefined Saprotroph	Microfungus
OTU136	*Bionectriaceae*	Pathotroph-Saprotroph	Algal Parasite-Bryophyte Parasite-Fungal Parasite-Undefined Saprotroph	Microfungus
OTU137	*Clavicipitaceae*	Pathotroph-Symbiotroph	Endophyte-Epiphyte-Fungal Parasite-Insect Parasite	Microfungus
OTU138	*Clavicipitaceae*	Pathotroph-Symbiotroph	Endophyte-Epiphyte-Fungal Parasite-Insect Parasite	Microfungus
OTU139	*Clavicipitaceae*	Pathotroph-Symbiotroph	Endophyte-Epiphyte-Fungal Parasite-Insect Parasite	Microfungus

OTU140	*Clavicipitaceae*	Pathotroph-Symbiotroph	Endophyte-Epiphyte-Fungal Parasite-Insect Parasite	Microfungus
OTU141	*Clavicipitaceae*	Pathotroph-Symbiotroph	Endophyte-Epiphyte-Fungal Parasite-Insect Parasite	Microfungus
OTU142	*Clavicipitaceae*	Pathotroph-Symbiotroph	Endophyte-Epiphyte-Fungal Parasite-Insect Parasite	Microfungus
OTU143	*Clavicipitaceae*	Pathotroph-Symbiotroph	Endophyte-Epiphyte-Fungal Parasite-Insect Parasite	Microfungus
OTU144	*Beauveria*	Pathotroph	Animal Pathogen	unassigned
OTU145	*Beauveria*	Pathotroph	Animal Pathogen	unassigned
OTU146	*Engyodontium album*	Pathotroph	Animal Pathogen	unassigned
OTU147	*Lecanicillium*	Pathotroph	Animal Pathogen	unassigned
OTU148	*Lecanicillium*	Pathotroph	Animal Pathogen	unassigned
OTU149	*Lecanicillium*	Pathotroph	Animal Pathogen	unassigned
OTU150	*Lecanicillium*	Pathotroph	Animal Pathogen	unassigned
OTU151	unassigned	unassigned	unassigned	unassigned
OTU152	*Simplicillium*	Pathotroph	Animal Pathogen	unassigned
OTU153	*Simplicillium*	Pathotroph	Animal Pathogen	unassigned
OTU154	unassigned	unassigned	unassigned	unassigned
OTU155	unassigned	unassigned	unassigned	unassigned
OTU156	*Monocillium*	Saprotroph	Undefined Saprotroph	unassigned
OTU157	*Trichoderma viride*	Pathotroph	Animal Pathogen	Microfungus
OTU158	*Acremonium alterunassignedtum*	Saprotroph	Wood Saprotroph	Microfungus
OTU159	*Acremonium*	Pathotroph-Saprotroph-Symbiotroph	Animal Pathogen-Endophyte-Fungal Parasite-Plant Pathogen-Wood Saprotroph	Microfungus
OTU160	*Acremonium charticola*	Pathotroph	Animal Pathogen	Microfungus
OTU161	*Acremonium*	Pathotroph-Saprotroph-Symbiotroph	Animal Pathogen-Endophyte-Fungal Parasite-Plant Pathogen-Wood Saprotroph	Microfungus
OTU162	*Acremonium furcatum*	Saprotroph	Wood Saprotroph	Microfungus
OTU163	*Acremonium persicinum*	Pathotroph	Animal Pathogen	Microfungus
OTU164	*Acremonium*	Pathotroph-Saprotroph-Symbiotroph	Animal Pathogen-Endophyte-Fungal Parasite-Plant Pathogen-Wood Saprotroph	Microfungus
OTU165	*Acremonium pteridii*	Symbiotroph	Endophyte	Microfungus
OTU166	*Acremonium*	Pathotroph-Saprotroph-Symbiotroph	Animal Pathogen-Endophyte-Fungal Parasite-Plant Pathogen-Wood Saprotroph	Microfungus
OTU167	*Acremonium*	Pathotroph-Saprotroph-Symbiotroph	Animal Pathogen-Endophyte-Fungal Parasite-Plant Pathogen-Wood Saprotroph	Microfungus
OTU168	*Trichothecium*	Pathotroph	Plant Pathogen	unassigned
OTU169	*Nectriaceae*	Pathotroph-Saprotroph-Symbiotroph	Animal Pathogen-Endophyte-Fungal Parasite-Lichen Parasite-Plant Pathogen-Wood Saprotroph	Microfungus
OTU170	*Nectriaceae*	Pathotroph-Saprotroph-Symbiotroph	Animal Pathogen-Endophyte-Fungal Parasite-Lichen Parasite-Plant Pathogen-Wood Saprotroph	Microfungus
OTU171	*Nectriaceae*	Pathotroph-Saprotroph-Symbiotroph	Animal Pathogen-Endophyte-Fungal Parasite-Lichen Parasite-Plant Pathogen-Wood Saprotroph	Microfungus
OTU172	*Nectriaceae*	Pathotroph-Saprotroph-Symbiotroph	Animal Pathogen-Endophyte-Fungal Parasite-Lichen Parasite-Plant Pathogen-Wood Saprotroph	Microfungus
OTU173	*Nectriaceae*	Pathotroph-Saprotroph-Symbiotroph	Animal Pathogen-Endophyte-Fungal Parasite-Lichen Parasite-Plant Pathogen-Wood Saprotroph	Microfungus
OTU174	*Nectriaceae*	Pathotroph-Saprotroph-Symbiotroph	Animal Pathogen-Endophyte-Fungal Parasite-Lichen Parasite-Plant Pathogen-Wood Saprotroph	Microfungus
OTU175	*Nectriaceae*	Pathotroph-Saprotroph-Symbiotroph	Animal Pathogen-Endophyte-Fungal Parasite-Lichen Parasite-Plant Pathogen-Wood Saprotroph	Microfungus
OTU176	*Nectriaceae*	Pathotroph-Saprotroph-Symbiotroph	Animal Pathogen-Endophyte-Fungal Parasite-Lichen Parasite-Plant Pathogen-Wood Saprotroph	Microfungus
OTU177	*Nectriaceae*	Pathotroph-Saprotroph-Symbiotroph	Animal Pathogen-Endophyte-Fungal Parasite-Lichen Parasite-Plant Pathogen-Wood Saprotroph	Microfungus
OTU178	*Nectriaceae*	Pathotroph-Saprotroph-Symbiotroph	Animal Pathogen-Endophyte-Fungal Parasite-Lichen Parasite-Plant Pathogen-Wood Saprotroph	Microfungus
OTU179	*Nectriaceae*	Pathotroph-Saprotroph-Symbiotroph	Animal Pathogen-Endophyte-Fungal Parasite-Lichen Parasite-Plant Pathogen-Wood Saprotroph	Microfungus
OTU180	*Nectriaceae*	Pathotroph-Saprotroph-Symbiotroph	Animal Pathogen-Endophyte-Fungal Parasite-Lichen Parasite-Plant Pathogen-Wood Saprotroph	Microfungus
OTU181	*Nectriaceae*	Pathotroph-Saprotroph-Symbiotroph	Animal Pathogen-Endophyte-Fungal Parasite-Lichen Parasite-Plant Pathogen-Wood Saprotroph	Microfungus
OTU182	*Nectriaceae*	Pathotroph-Saprotroph-Symbiotroph	Animal Pathogen-Endophyte-Fungal Parasite-Lichen Parasite-Plant Pathogen-Wood Saprotroph	Microfungus
OTU183	*Nectriaceae*	Pathotroph-Saprotroph-Symbiotroph	Animal Pathogen-Endophyte-Fungal Parasite-Lichen Parasite-Plant Pathogen-Wood Saprotroph	Microfungus
OTU184	*Nectriaceae*	Pathotroph-Saprotroph-Symbiotroph	Animal Pathogen-Endophyte-Fungal Parasite-Lichen Parasite-Plant Pathogen-Wood Saprotroph	Microfungus
OTU185	*Nectriaceae*	Pathotroph-Saprotroph-Symbiotroph	Animal Pathogen-Endophyte-Fungal Parasite-Lichen Parasite-Plant Pathogen-Wood Saprotroph	Microfungus
OTU186	*Purpureocillium*	Pathotroph	Fungal Parasite	unassigned
OTU187	*Purpureocillium*	Pathotroph	Fungal Parasite	unassigned
OTU188	*Tolypocladium*	Pathotroph-Symbiotroph	Animal Pathogen-Clavicipitaceous Endophyte-Fungal Parasite	unassigned
OTU189	unassigned	unassigned	unassigned	unassigned
OTU190	*Halosphaeriaceae*	Saprotroph	Undefined Saprotroph-Wood Saprotroph	Microfungus

OTU191	*Microascaceae*	Pathotroph-Saprotroph-Symbiotroph	Animal Pathogen-Endophyte-Plant Pathogen-Undefined Saprotroph	Microfungus
OTU192	*Microascaceae*	Pathotroph-Saprotroph-Symbiotroph	Animal Pathogen-Endophyte-Plant Pathogen-Undefined Saprotroph	Microfungus
OTU193	*Microascaceae*	Pathotroph-Saprotroph-Symbiotroph	Animal Pathogen-Endophyte-Plant Pathogen-Undefined Saprotroph	Microfungus
OTU194	*Microascaceae*	Pathotroph-Saprotroph-Symbiotroph	Animal Pathogen-Endophyte-Plant Pathogen-Undefined Saprotroph	Microfungus
OTU195	*Microascaceae*	Pathotroph-Saprotroph-Symbiotroph	Animal Pathogen-Endophyte-Plant Pathogen-Undefined Saprotroph	Microfungus
OTU196	*Microascaceae*	Pathotroph-Saprotroph-Symbiotroph	Animal Pathogen-Endophyte-Plant Pathogen-Undefined Saprotroph	Microfungus
OTU197	*Microascaceae*	Pathotroph-Saprotroph-Symbiotroph	Animal Pathogen-Endophyte-Plant Pathogen-Undefined Saprotroph	Microfungus
OTU198	*Microascaceae*	Pathotroph-Saprotroph-Symbiotroph	Animal Pathogen-Endophyte-Plant Pathogen-Undefined Saprotroph	Microfungus
OTU199	*Microascaceae*	Pathotroph-Saprotroph-Symbiotroph	Animal Pathogen-Endophyte-Plant Pathogen-Undefined Saprotroph	Microfungus
OTU200	*Microascaceae*	Pathotroph-Saprotroph-Symbiotroph	Animal Pathogen-Endophyte-Plant Pathogen-Undefined Saprotroph	Microfungus
OTU201	unassigned	unassigned	unassigned	unassigned
OTU202	*Cephalothecaceae*	Saprotroph	Fungal Parasite-Wood Saprotroph	Microfungus
OTU203	*Botryotrichum*	Saprotroph	Undefined Saprotroph	unassigned
OTU204	unassigned	unassigned	unassigned	unassigned
OTU205	unassigned	unassigned	unassigned	unassigned
OTU206	unassigned	unassigned	unassigned	unassigned
OTU207	unassigned	unassigned	unassigned	unassigned
OTU208	*Trichocladium*	Saprotroph	Undefined Saprotroph	unassigned
OTU209	unassigned	unassigned	unassigned	unassigned
OTU210	unassigned	unassigned	unassigned	unassigned
OTU211	*Lasiosphaeriaceae*	Saprotroph	Undefined Saprotroph	Microfungus
OTU212	*Lasiosphaeriaceae*	Saprotroph	Undefined Saprotroph	Microfungus
OTU213	*Lasiosphaeriaceae*	Saprotroph	Undefined Saprotroph	Microfungus
OTU214	*Lasiosphaeriaceae*	Saprotroph	Undefined Saprotroph	Microfungus
OTU215	unassigned	unassigned	unassigned	unassigned
OTU216	*Staphylotrichum*	Saprotroph	Undefined Saprotroph	unassigned
OTU217	*Staphylotrichum*	Saprotroph	Undefined Saprotroph	unassigned
OTU218	unassigned	unassigned	unassigned	unassigned
OTU219	unassigned	unassigned	unassigned	unassigned
OTU220	unassigned	unassigned	unassigned	unassigned
OTU221	unassigned	unassigned	unassigned	unassigned
OTU222	*Coniocessia*	Saprotroph	Undefined Saprotroph	unassigned
OTU223	*Idriella luunassignedta*	Pathotroph	Plant Pathogen	unassigned
OTU224	*Xylariaceae*	Pathotroph-Saprotroph-Symbiotroph	Dung Saprotroph-Endophyte-Plant Pathogen-Undefined Saprotroph	Xylarioid
OTU225	*Xylariaceae*	Pathotroph-Saprotroph-Symbiotroph	Dung Saprotroph-Endophyte-Plant Pathogen-Undefined Saprotroph	Xylarioid
OTU226	*Xylariaceae*	Pathotroph-Saprotroph-Symbiotroph	Dung Saprotroph-Endophyte-Plant Pathogen-Undefined Saprotroph	Xylarioid
OTU227	*Xylariaceae*	Pathotroph-Saprotroph-Symbiotroph	Dung Saprotroph-Endophyte-Plant Pathogen-Undefined Saprotroph	Xylarioid
OTU228	*Xylariaceae*	Pathotroph-Saprotroph-Symbiotroph	Dung Saprotroph-Endophyte-Plant Pathogen-Undefined Saprotroph	Xylarioid
OTU229	*Hansfordia*	Saprotroph	Undefined Saprotroph	unassigned
OTU230	unassigned	unassigned	unassigned	unassigned
OTU231	unassigned	unassigned	unassigned	unassigned
OTU232	*Agaricaceae*	Saprotroph	Undefined Saprotroph	Agaricoid-Gasteroid-Secotioid
OTU233	*Agaricaceae*	Saprotroph	Undefined Saprotroph	Agaricoid-Gasteroid-Secotioid
OTU234	*Agaricaceae*	Saprotroph	Undefined Saprotroph	Agaricoid-Gasteroid-Secotioid
OTU235	*Agaricaceae*	Saprotroph	Undefined Saprotroph	Agaricoid-Gasteroid-Secotioid
OTU236	*Agaricales*	Pathotroph-Saprotroph-Symbiotroph	Bryophyte Parasite-Dung Saprotroph-Ectomycorrhizal-Fungal Parasite-Leaf Saprotroph-Plant Parasite-Undefined Saprotroph-Wood Saprotroph	Agaricoid-Gasteroid-Microfungus-Secotioid-Yeast
OTU237	*Bolbitiaceae*	Saprotroph	Dung Saprotroph-Plant Saprotroph-Soil Saprotroph	Agaricoid
OTU238	*Clavariaceae*	Saprotroph-Symbiotroph	Lichenized-Undefined Saprotroph	Clavarioid
OTU239	*Clavariaceae*	Saprotroph-Symbiotroph	Lichenized-Undefined Saprotroph	Clavarioid
OTU240	*Clavariaceae*	Saprotroph-Symbiotroph	Lichenized-Undefined Saprotroph	Clavarioid
OTU241	*Clavariaceae*	Saprotroph-Symbiotroph	Lichenized-Undefined Saprotroph	Clavarioid

OTU242	*Crepidotaceae*	Saprotroph	Wood Saprotroph	Agaricoid
OTU243	*Cystostereaceae*	Saprotroph	Wood Saprotroph	Corticioid
OTU244	*Entolomataceae*	Pathotroph-Saprotroph-Symbiotroph	Ectomycorrhizal-Fungal Parasite-Soil Saprotroph-Undefined Saprotroph	Agaricoid
OTU245	*Entolomataceae*	Pathotroph-Saprotroph-Symbiotroph	Ectomycorrhizal-Fungal Parasite-Soil Saprotroph-Undefined Saprotroph	Agaricoid
OTU246	*Agaricales*	Pathotroph-Saprotroph-Symbiotroph	Bryophyte Parasite-Dung Saprotroph-Ectomycorrhizal-Fungal Parasite-Leaf Saprotroph-Plant Parasite-Undefined Saprotroph-Wood Saprotroph	Agaricoid-Gasteroid-Microfungus-Secotioid-Yeast
OTU247	*Agaricales*	Pathotroph-Saprotroph-Symbiotroph	Bryophyte Parasite-Dung Saprotroph-Ectomycorrhizal-Fungal Parasite-Leaf Saprotroph-Plant Parasite-Undefined Saprotroph-Wood Saprotroph	Agaricoid-Gasteroid-Microfungus-Secotioid-Yeast
OTU248	*Agaricales*	Pathotroph-Saprotroph-Symbiotroph	Bryophyte Parasite-Dung Saprotroph-Ectomycorrhizal-Fungal Parasite-Leaf Saprotroph-Plant Parasite-Undefined Saprotroph-Wood Saprotroph	Agaricoid-Gasteroid-Microfungus-Secotioid-Yeast
OTU249	*Inocybaceae*	Symbiotroph	Ectomycorrhizal	Agaricoid
OTU250	*Inocybaceae*	Symbiotroph	Ectomycorrhizal	Agaricoid
OTU251	*Agaricales*	Pathotroph-Saprotroph-Symbiotroph	Bryophyte Parasite-Dung Saprotroph-Ectomycorrhizal-Fungal Parasite-Leaf Saprotroph-Plant Parasite-Undefined Saprotroph-Wood Saprotroph	Agaricoid-Gasteroid-Microfungus-Secotioid-Yeast
OTU252	*Agaricales*	Pathotroph-Saprotroph-Symbiotroph	Bryophyte Parasite-Dung Saprotroph-Ectomycorrhizal-Fungal Parasite-Leaf Saprotroph-Plant Parasite-Undefined Saprotroph-Wood Saprotroph	Agaricoid-Gasteroid-Microfungus-Secotioid-Yeast
OTU253	*Marasmiaceae*	Pathotroph-Saprotroph-Symbiotroph	Endophyte-Plant Pathogen-Undefined Saprotroph	Agaricoid
OTU254	*Omphalotaceae*	Saprotroph	Wood Saprotroph	Agaricoid
OTU255	*Omphalotaceae*	Saprotroph	Wood Saprotroph	Agaricoid
OTU256	*Pluteaceae*	Pathotroph-Saprotroph	Bryophyte Parasite-Ectomycorrhizal-Litter Saprotroph-Wood Saprotroph	Agaricoid
OTU257	*Pluteaceae*	Pathotroph-Saprotroph	Bryophyte Parasite-Ectomycorrhizal-Litter Saprotroph-Wood Saprotroph	Agaricoid
OTU258	*Psathyrellaceae*	Saprotroph	Dung Saprotroph-Plant Saprotroph-Wood Saprotroph	Agaricoid
OTU259	*Psathyrellaceae*	Saprotroph	Dung Saprotroph-Plant Saprotroph-Wood Saprotroph	Agaricoid
OTU260	*Pterulaceae*	Saprotroph	Wood Saprotroph	Clavarioid
OTU261	*Stephanosporaceae*	Saprotroph	Plant Saprotroph-Wood Saprotroph	Corticioid-Gasteroid
OTU262	*Strophariaceae*	Pathotroph-Saprotroph	Dung Saprotroph-Plant Parasite-Soil Saprotroph-Undefined Saprotroph-Wood Saprotroph	Agaricoid
OTU263	*Strophariaceae*	Pathotroph-Saprotroph	Dung Saprotroph-Plant Parasite-Soil Saprotroph-Undefined Saprotroph-Wood Saprotroph	Agaricoid
OTU264	*Tricholomataceae*	Pathotroph-Symbiotroph	Ectomycorrhizal-Fungal Parasite	Agaricoid
OTU265	*Tricholomataceae*	Pathotroph-Symbiotroph	Ectomycorrhizal-Fungal Parasite	Agaricoid
OTU266	*Tricholomataceae*	Pathotroph-Symbiotroph	Ectomycorrhizal-Fungal Parasite	Agaricoid
OTU267	*Tricholomataceae*	Pathotroph-Symbiotroph	Ectomycorrhizal-Fungal Parasite	Agaricoid
OTU268	*Tricholomataceae*	Pathotroph-Symbiotroph	Ectomycorrhizal-Fungal Parasite	Agaricoid
OTU269	*Tricholomataceae*	Pathotroph-Symbiotroph	Ectomycorrhizal-Fungal Parasite	Agaricoid
OTU270	*Agaricales*	Pathotroph-Saprotroph-Symbiotroph	Bryophyte Parasite-Dung Saprotroph-Ectomycorrhizal-Fungal Parasite-Leaf Saprotroph-Plant Parasite-Undefined Saprotroph-Wood Saprotroph	Agaricoid-Gasteroid-Microfungus-Secotioid-Yeast
OTU271	*Agaricales*	Pathotroph-Saprotroph-Symbiotroph	Bryophyte Parasite-Dung Saprotroph-Ectomycorrhizal-Fungal Parasite-Leaf Saprotroph-Plant Parasite-Undefined Saprotroph-Wood Saprotroph	Agaricoid-Gasteroid-Microfungus-Secotioid-Yeast
OTU272	*Agaricales*	Pathotroph-Saprotroph-Symbiotroph	Bryophyte Parasite-Dung Saprotroph-Ectomycorrhizal-Fungal Parasite-Leaf Saprotroph-Plant Parasite-Undefined Saprotroph-Wood Saprotroph	Agaricoid-Gasteroid-Microfungus-Secotioid-Yeast
OTU273	*Agaricales*	Pathotroph-Saprotroph-Symbiotroph	Bryophyte Parasite-Dung Saprotroph-Ectomycorrhizal-Fungal Parasite-Leaf Saprotroph-Plant Parasite-Undefined Saprotroph-Wood Saprotroph	Agaricoid-Gasteroid-Microfungus-Secotioid-Yeast
OTU274	*Xeunassignedsmatella*	Saprotroph	Undefined Saprotroph	unassigned
OTU275	*Xeunassignedsmatella*	Saprotroph	Undefined Saprotroph	unassigned
OTU276	*Xeunassignedsmatella*	Saprotroph	Undefined Saprotroph	unassigned
OTU277	*Atheliaceae*	Pathotroph-Saprotroph-Symbiotroph	Ectomycorrhizal-Lichen Parasite-Lichenized-Plant Pathogen	Corticioid
OTU278	*Auriculariaceae*	Pathotroph-Saprotroph	Plant Parasite-Wood Saprotroph	Auriculariod
OTU279	*Exidia*	Saprotroph-Symbiotroph	Endophyte-Undefined Saprotroph	Tremelloid
OTU280	*Myxarium*	Saprotroph	Undefined Saprotroph	unassigned
OTU281	*Myxarium*	Saprotroph	Undefined Saprotroph	unassigned
OTU282	*Protomerulius*	Saprotroph	Undefined Saprotroph	Corticioid
OTU283	*Stypella*	Saprotroph	Undefined Saprotroph	unassigned
OTU284	unassigned	unassigned	unassigned	unassigned
OTU285	unassigned	unassigned	unassigned	unassigned
OTU286	*Boletaceae*	Pathotroph-Saprotroph-Symbiotroph	Ectomycorrhizal-Fungal Parasite-Plant Saprotroph-Wood Saprotroph	Boletoid
OTU287	*Melanogaster*	Symbiotroph	Ectomycorrhizal	Gasteroid
OTU288	*Suillus*	Symbiotroph	Ectomycorrhizal	Boletoid
OTU289	unassigned	unassigned	unassigned	unassigned
OTU290	*Sistotrema*	Saprotroph-Symbiotroph	Ectomycorrhizal-Wood Saprotroph	Corticioid
OTU291	*Sistotrema*	Saprotroph-Symbiotroph	Ectomycorrhizal-Wood Saprotroph	Corticioid
OTU292	*Sistotrema*	Saprotroph-Symbiotroph	Ectomycorrhizal-Wood Saprotroph	Corticioid

OTU293	unassigned	unassigned	unassigned	unassigned
OTU294	*Ceratobasidiaceae*	Pathotroph-Saprotroph-Symbiotroph	Endomycorrhizal-Plant Pathogen-Undefined Saprotroph	Microfungus
OTU295	*Ceratobasidiaceae*	Pathotroph-Saprotroph-Symbiotroph	Endomycorrhizal-Plant Pathogen-Undefined Saprotroph	Microfungus
OTU296	*Tulasnellaceae*	Saprotroph-Symbiotroph	Ectomycorrhizal-Undefined Saprotroph	Corticioid
OTU297	*Corticiaceae*	Pathotroph-Saprotroph	Lichen Parasite-Plant Pathogen-Wood Saprotroph	Corticioid-Microfungus
OTU298	unassigned	unassigned	unassigned	unassigned
OTU299	*Gomphaceae*	Saprotroph-Symbiotroph	Ectomycorrhizal-Undefined Saprotroph-Wood Saprotroph	Clavarioid-Gomphoid
OTU300	*Hymenochaetaceae*	Saprotroph-Symbiotroph	Ectomycorrhizal-Wood Saprotroph	Corticioid-Polyporoid
OTU301	*Hymenochaetaceae*	Saprotroph-Symbiotroph	Ectomycorrhizal-Wood Saprotroph	Corticioid-Polyporoid
OTU302	*Hymenochaetaceae*	Saprotroph-Symbiotroph	Ectomycorrhizal-Wood Saprotroph	Corticioid-Polyporoid
OTU303	*Hymenochaetaceae*	Saprotroph-Symbiotroph	Ectomycorrhizal-Wood Saprotroph	Corticioid-Polyporoid
OTU304	*Peniophorella*	Saprotroph	Undefined Saprotroph	Corticioid
OTU305	*Peniophorella*	Saprotroph	Undefined Saprotroph	Corticioid
OTU306	*Resinicium bicolor*	Pathotroph	Plant Pathogen	Corticioid
OTU307	*Resinicium*	Saprotroph	Undefined Saprotroph	Corticioid
OTU308	*Sidera*	Saprotroph	Undefined Saprotroph	unassigned
OTU309	*Schizoporaceae*	Saprotroph	Wood Saprotroph	Corticioid
OTU310	*Schizoporaceae*	Saprotroph	Wood Saprotroph	Corticioid
OTU311	*Schizoporaceae*	Saprotroph	Wood Saprotroph	Corticioid
OTU312	*Schizoporaceae*	Saprotroph	Wood Saprotroph	Corticioid
OTU313	*Schizoporaceae*	Saprotroph	Wood Saprotroph	Corticioid
OTU314	*Schizoporaceae*	Saprotroph	Wood Saprotroph	Corticioid
OTU315	*Schizoporaceae*	Saprotroph	Wood Saprotroph	Corticioid
OTU316	*Phallaceae*	Saprotroph	Litter Saprotroph-Soil Saprotroph-Wood Saprotroph	Phalloid
OTU317	*Fomitopsidaceae*	Pathotroph-Saprotroph	Plant Pathogen-Wood Saprotroph	Polyporoid
OTU318	*Fomitopsidaceae*	Pathotroph-Saprotroph	Plant Pathogen-Wood Saprotroph	Polyporoid
OTU319	*Fomitopsidaceae*	Pathotroph-Saprotroph	Plant Pathogen-Wood Saprotroph	Polyporoid
OTU320	*Ganodermataceae*	Pathotroph-Saprotroph	Plant Pathogen-Wood Saprotroph	Polyporoid
OTU321	*Byssomerulius*	Saprotroph	Undefined Saprotroph	unassigned
OTU322	*Ceriporia*	Saprotroph	Wood Saprotroph	Corticioid
OTU323	*Ceriporia*	Saprotroph	Wood Saprotroph	Corticioid
OTU324	*Ceriporia*	Saprotroph	Wood Saprotroph	Corticioid
OTU325	*Meruliaceae*	Pathotroph-Saprotroph	Plant Pathogen-Undefined Saprotroph	Corticioid-Polyporoid
OTU326	*Meruliaceae*	Pathotroph-Saprotroph	Plant Pathogen-Undefined Saprotroph	Corticioid-Polyporoid
OTU327	*Meruliaceae*	Pathotroph-Saprotroph	Plant Pathogen-Undefined Saprotroph	Corticioid-Polyporoid
OTU328	*Meruliaceae*	Pathotroph-Saprotroph	Plant Pathogen-Undefined Saprotroph	Corticioid-Polyporoid
OTU329	*Meruliaceae*	Pathotroph-Saprotroph	Plant Pathogen-Undefined Saprotroph	Corticioid-Polyporoid
OTU330	*Meruliaceae*	Pathotroph-Saprotroph	Plant Pathogen-Undefined Saprotroph	Corticioid-Polyporoid
OTU331	*Meruliaceae*	Pathotroph-Saprotroph	Plant Pathogen-Undefined Saprotroph	Corticioid-Polyporoid
OTU332	*Meruliaceae*	Pathotroph-Saprotroph	Plant Pathogen-Undefined Saprotroph	Corticioid-Polyporoid
OTU333	*Bjerkandera*	Saprotroph	Wood Saprotroph	Polyporoid
OTU334	*Hyphodermella corrugata*	Pathotroph	Plant Pathogen	unassigned
OTU335	*Hyphodermella*	Saprotroph	Undefined Saprotroph	unassigned
OTU336	unassigned	unassigned	unassigned	unassigned
OTU337	*Phanerochaete*	Saprotroph	Wood Saprotroph	Corticioid
OTU338	*Phanerochaete*	Saprotroph	Wood Saprotroph	Corticioid
OTU339	*Teraunassigned*	Saprotroph	Undefined Saprotroph	unassigned
OTU340	*Abortiporus*	Saprotroph	Wood Saprotroph	Polyporoid
OTU341	*Polyporaceae*	Saprotroph	Wood Saprotroph	Polyporoid
OTU342	*Polyporaceae*	Saprotroph	Wood Saprotroph	Polyporoid
OTU343	*Steccheriunassignedceae*	Saprotroph	Wood Saprotroph	Corticioid-Polyporoid

OTU344	*Steccheriunassignedceae*	Saprotroph	Wood Saprotroph	Corticioid-Polyporoid
OTU345	*Steccheriunassignedceae*	Saprotroph	Wood Saprotroph	Corticioid-Polyporoid
OTU346	unassigned	unassigned	unassigned	unassigned
OTU347	*Xeunassignedsma*	Saprotroph	Undefined Saprotroph	unassigned
OTU348	*Bondarzewiaceae*	Pathotroph-Saprotroph	Plant Pathogen-Wood Saprotroph	Polyporoid
OTU349	*Gloiothele*	Saprotroph	Undefined Saprotroph	Corticioid
OTU350	unassigned	unassigned	unassigned	unassigned
OTU351	*Peniophoraceae*	Pathotroph-Saprotroph	Plant Pathogen-Wood Saprotroph	Corticioid
OTU352	*Peniophoraceae*	Pathotroph-Saprotroph	Plant Pathogen-Wood Saprotroph	Corticioid
OTU353	*Peniophoraceae*	Pathotroph-Saprotroph	Plant Pathogen-Wood Saprotroph	Corticioid
OTU354	*Peniophoraceae*	Pathotroph-Saprotroph	Plant Pathogen-Wood Saprotroph	Corticioid
OTU355	*Peniophoraceae*	Pathotroph-Saprotroph	Plant Pathogen-Wood Saprotroph	Corticioid
OTU356	*Stereaceae*	Saprotroph	Undefined Saprotroph-Wood Saprotroph	Corticioid-Polyporoid
OTU357	*Stereaceae*	Saprotroph	Undefined Saprotroph-Wood Saprotroph	Corticioid-Polyporoid
OTU358	*Sebaciunassignedceae*	Symbiotroph	Ectomycorrhizal	Coralloid
OTU359	*Sebaciunassignedceae*	Symbiotroph	Ectomycorrhizal	Coralloid
OTU360	*Sebaciunassignedceae*	Symbiotroph	Ectomycorrhizal	Coralloid
OTU361	*Serendipita*	Symbiotroph	Orchid Mycorrhizal	unassigned
OTU362	unassigned	unassigned	unassigned	unassigned
OTU363	unassigned	unassigned	unassigned	unassigned
OTU364	*Thelephoraceae*	Saprotroph-Symbiotroph	Ectomycorrhizal-Undefined Saprotroph	Clavarioid
OTU365	*Thelephoraceae*	Saprotroph-Symbiotroph	Ectomycorrhizal-Undefined Saprotroph	Clavarioid
OTU366	*Thelephoraceae*	Saprotroph-Symbiotroph	Ectomycorrhizal-Undefined Saprotroph	Clavarioid
OTU367	*Thelephoraceae*	Saprotroph-Symbiotroph	Ectomycorrhizal-Undefined Saprotroph	Clavarioid
OTU368	*Thelephoraceae*	Saprotroph-Symbiotroph	Ectomycorrhizal-Undefined Saprotroph	Clavarioid
OTU369	*Brevicellicium*	Saprotroph	Undefined Saprotroph	unassigned
OTU370	*Trechispora*	Saprotroph	Wood Saprotroph	Corticioid
OTU371	*Trechispora*	Saprotroph	Wood Saprotroph	Corticioid
OTU372	*Trechispora*	Saprotroph	Wood Saprotroph	Corticioid
OTU373	*Trechispora*	Saprotroph	Wood Saprotroph	Corticioid
OTU374	*Sistotremastrum*	Saprotroph	Undefined Saprotroph	unassigned
OTU375	unassigned	unassigned	unassigned	unassigned
OTU376	unassigned	unassigned	unassigned	unassigned
OTU377	*Chionosphaeraceae*	Pathotroph-Saprotroph	Fungal Parasite-Undefined Saprotroph	Microfungus
OTU378	*Bensingtonia*	Saprotroph	Undefined Saprotroph	Facultative Yeast
OTU379	*Helicogloea*	Saprotroph	Undefined Saprotroph	unassigned
OTU380	*Buckleyzyma*	Saprotroph	Undefined Saprotroph	Yeast
OTU381	*Sakaguchia*	Saprotroph	Undefined Saprotroph	Yeast
OTU382	unassigned	unassigned	unassigned	unassigned
OTU383	unassigned	unassigned	unassigned	unassigned
OTU384	*Basidioascus*	Saprotroph	Undefined Saprotroph	Microfungus
OTU385	*Geminibasidium*	Saprotroph	Undefined Saprotroph	unassigned
OTU386	unassigned	unassigned	unassigned	unassigned
OTU387	*Malassezia globosa*	Pathotroph	Animal Pathogen	Facultative Yeast
OTU388	*Malassezia restricta*	Pathotroph	Animal Pathogen	unassigned
OTU389	*Sporidiobolaceae*	Pathotroph-Saprotroph	Animal Pathogen-Undefined Saprotroph	Microfungus
OTU390	*Sporidiobolaceae*	Pathotroph-Saprotroph	Animal Pathogen-Undefined Saprotroph	Microfungus
OTU391	*Sporidiobolaceae*	Pathotroph-Saprotroph	Animal Pathogen-Undefined Saprotroph	Microfungus
OTU392	*Sporidiobolaceae*	Pathotroph-Saprotroph	Animal Pathogen-Undefined Saprotroph	Microfungus
OTU393	unassigned	unassigned	unassigned	unassigned
OTU394	unassigned	unassigned	unassigned	unassigned

OTU395	unassigned	unassigned	unassigned	unassigned
OTU396	*Tausonia pullulans*	Saprotroph	Undefined Saprotroph	Yeast
OTU397	unassigned	unassigned	unassigned	unassigned
OTU398	*Filobasidium*	Saprotroph	Undefined Saprotroph	Facultative Yeast
OTU399	unassigned	unassigned	unassigned	unassigned
OTU400	unassigned	unassigned	unassigned	unassigned
OTU401	*Kockovaella*	Symbiotroph	Epiphyte	Yeast
OTU402	*unassigned*	unassigned	unassigned	unassigned
OTU403	*unassigned*	unassigned	unassigned	unassigned
OTU404	*unassigned*	unassigned	unassigned	unassigned
OTU405	*Apiotrichum*	Saprotroph	Soil Saprotroph	Microfungus
OTU406	*Apiotrichum*	Saprotroph	Soil Saprotroph	Microfungus
OTU407	*Apiotrichum*	Saprotroph	Soil Saprotroph	Microfungus
OTU408	*Cutaneotrichosporon*	Pathotroph	Animal Pathogen	Yeast
OTU409	*Tritirachium*	Saprotroph	Undefined Saprotroph	unassigned
OTU410	*unassigned*	unassigned	unassigned	unassigned
OTU411	*Chytridiaceae*	Pathotroph-Saprotroph	Algal Parasite-Fungal Parasite-Undefined Saprotroph	Microfungus
OTU412	*unassigned*	unassigned	unassigned	unassigned
OTU413	*Diversisporaceae*	Symbiotroph	Arbuscular Mycorrhizal	unassigned
OTU414	*Glomeraceae*	Symbiotroph	Arbuscular Mycorrhizal	Microfungus
OTU415	*Glomeraceae*	Symbiotroph	Arbuscular Mycorrhizal	Microfungus
OTU416	*Glomeraceae*	Symbiotroph	Arbuscular Mycorrhizal	Microfungus
OTU417	*unassigned*	unassigned	unassigned	unassigned
OTU418	*Ramicandelaber*	Saprotroph	Undefined Saprotroph	unassigned
OTU419	*Mortierellaceae*	Saprotroph-Symbiotroph	Endophyte-Litter Saprotroph-Soil Saprotroph-Undefined Saprotroph	Microfungus
OTU420	*Mortierellaceae*	Saprotroph-Symbiotroph	Endophyte-Litter Saprotroph-Soil Saprotroph-Undefined Saprotroph	Microfungus
OTU421	*Mortierellaceae*	Saprotroph-Symbiotroph	Endophyte-Litter Saprotroph-Soil Saprotroph-Undefined Saprotroph	Microfungus
OTU422	*Mortierellaceae*	Saprotroph-Symbiotroph	Endophyte-Litter Saprotroph-Soil Saprotroph-Undefined Saprotroph	Microfungus
OTU423	*Mortierellaceae*	Saprotroph-Symbiotroph	Endophyte-Litter Saprotroph-Soil Saprotroph-Undefined Saprotroph	Microfungus
OTU424	*Mortierellaceae*	Saprotroph-Symbiotroph	Endophyte-Litter Saprotroph-Soil Saprotroph-Undefined Saprotroph	Microfungus
OTU425	*Mortierellaceae*	Saprotroph-Symbiotroph	Endophyte-Litter Saprotroph-Soil Saprotroph-Undefined Saprotroph	Microfungus
OTU426	*Mortierellaceae*	Saprotroph-Symbiotroph	Endophyte-Litter Saprotroph-Soil Saprotroph-Undefined Saprotroph	Microfungus
OTU427	*Mortierellaceae*	Saprotroph-Symbiotroph	Endophyte-Litter Saprotroph-Soil Saprotroph-Undefined Saprotroph	Microfungus
OTU428	*Mortierellaceae*	Saprotroph-Symbiotroph	Endophyte-Litter Saprotroph-Soil Saprotroph-Undefined Saprotroph	Microfungus
OTU429	*Chaetocladium*	Saprotroph	Undefined Saprotroph	unassigned
OTU430	*Mucor circinelloides*	Pathotroph	Animal Pathogen-Plant Pathogen	Facultative Yeast
OTU431	*Mucor fragilis*	Saprotroph	Dung Saprotroph	unassigned
OTU432	*unassigned*	unassigned	unassigned	unassigned
OTU433	*Mucor racemosus*	Pathotroph-Symbiotroph	Endophyte-Plant Pathogen	unassigned
OTU434	*Parasitella*	Saprotroph	Undefined Saprotroph	unassigned
OTU435	*unassigned*	unassigned	unassigned	unassigned
OTU436	*unassigned*	unassigned	unassigned	unassigned
OTU437	*Syncephalis*	Pathotroph	Fungal Parasite	unassigned

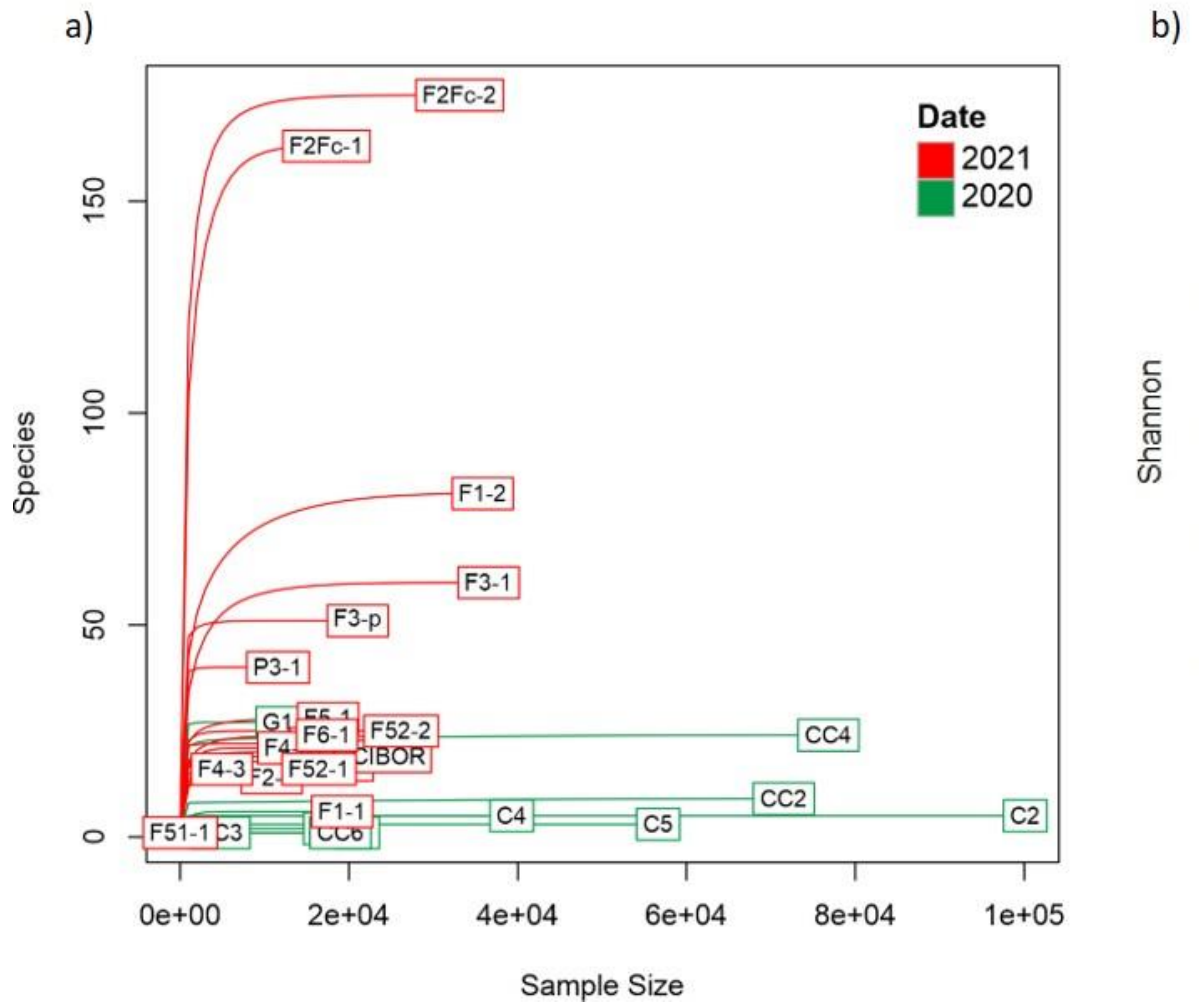

Figure S1. Alpha diversity analysis. a) Rarefaction curves and b) Boxplor showing Shannon indices
